# Reduction of Carboxylic Acids to Alcohols via Manganese(I) Catalyzed Hydrosilylation

**DOI:** 10.1021/jacsau.1c00140

**Published:** 2021-05-11

**Authors:** Emanuele Antico, Peter Schlichter, Christophe Werlé, Walter Leitner

**Affiliations:** † Max Planck Institute for Chemical Energy Conversion, Stiftstr. 34−36, 45470 Mülheim an der Ruhr, Germany; ‡ Institut für Technische und Makromolekulare Chemie (ITMC), RWTH Aachen University, Worringer Weg 2, 52074 Aachen, Germany; § Ruhr University Bochum, Universitätsstr. 150, 44801 Bochum, Germany

**Keywords:** Carboxylic Acids, Alcohols, Manganese, Homogeneous, Catalysis, Reduction Reactions, Hydrosilylation

## Abstract

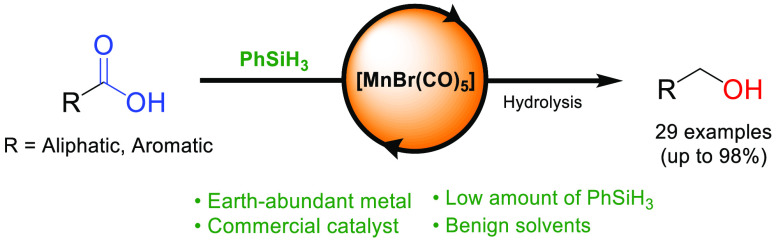

The reduction of carboxylic acids to the respective alcohols, in mild conditions, was achieved using [MnBr(CO)_5_] as the catalyst and bench stable PhSiH_3_ as the reducing agent. It was shown that the reaction with the earth-abundant metal catalyst could be performed either with a catalyst loading as low as 0.5 mol %, rare with the use of [MnBr(CO)_5_], or on a gram scale employing only 1.5 equiv of PhSiH_3_, the lowest amount of silane reported to date for this transformation. Kinetic data and control experiments have provided initial insight into the mechanism of the catalytic process, suggesting that it proceeds via the formation of silyl ester intermediates and ligand dissociation to generate a coordinatively unsaturated Mn(I) complex as the active species.

## Introduction

The direct reduction of carboxylic acids to alcohols is a more challenging transformation compared to the respective reduction of other carbonyl or carboxyl derivatives. It is traditionally performed using stoichiometric or even excess amounts of strong reducing reagents such as diborane, LiAlH_4_, or DIBAL-H. Also, excesses of milder reductants such as NaBH_4_ can be used in combination with stoichiometric activating agents (e.g., I_2_,^1^ catechol, or trifluoroacetic acid^2^). Since alcohols are widely employed in many chemical industries,^3^ the development of catalytic methods to reduce carboxylic acids to alcohols continues to be a highly active research field. While the use of hydrogen offers a highly attractive reduction path, there are few reported examples in the literature for catalytic hydrogenation of carboxylic acids to alcohols,^4−11^ usually requiring rather high H_2_ pressures. The catalysts are typically based on expensive noble metals and specialized ligands with only one example using an earth-abundant metal catalyst.^12^ This scarcity results inter alia from the Brønsted acidity and coordinative ability of carboxylic acids, which can deactivate the catalysts by interfering with the metal, the ligand, and possible metal–ligand cooperative pathways.^13−15^ An alternative route to the use of traditional stoichiometric metal hydrides is offered by easy-to-handle and safe silane reagents.^16^ There are many examples reported for the hydrosilylation of esters^17^ and recently also for amides.^18−21^ However, only very few examples exist for free carboxylic acids: they require a large excess of the silane reagent; make use of noble metal catalysts like Ru,^22,23^ Rh,^24^ and Ir;^25^ are performed in halogenated solvents like chloroform;^26^ or require a specific experimental apparatus.^27,28^ Therefore, the development of a direct catalytic reduction from free carboxylic acids to their corresponding alcohols still seems highly desirable.^29,30^

Until recently, only very few earth-abundant base-metal catalysts were known for carboxylic acid hydrosilylation. Lemaire and co-workers reported that Cu(OTf)_2_, used in high catalyst loading, was able to reduce a selection of carboxylic acids into their respective alcohols.^31^ Two more examples were presented by the groups of Sortais and Darcel, who utilized metal carbonyl complexes activated under photochemical irradiation: the complex [Fe(COD)(CO)_3_], which afforded alcohols from aliphatic carboxylic acids in high yields (including only a couple of aromatic substrates in moderate yields);^27^ and the complex [Mn_2_(CO)_10_], which was thermally inactive and used under UV light, leading to aldehydes as the products.^28^ More recently, our group reported the use of a manganese(I) pincer complex in the hydrosilylative reduction of a range of carbonyl functional groups, including a few carboxylic acid examples.^32^ Manganese(I) complexes have been employed in hydrosilylation reactions to reduce carbonyl groups since the work of Cutler and co-workers in 1995^33^ with multiple recent works focusing on similar hydrosilylative transformations catalyzed by Mn(I) complexes as a result of manganese’s high natural abundance, low toxicity, and low price.^35−41^ Since the direct reduction of carboxylic acids to alcohols still remains an underexplored field, it was decided to further explore the development of an easy to handle and well-performing Mn(I) catalyst for the hydrosilylation of carboxylic acids to alcohols.

## Results and Discussion

**Scheme 1:**
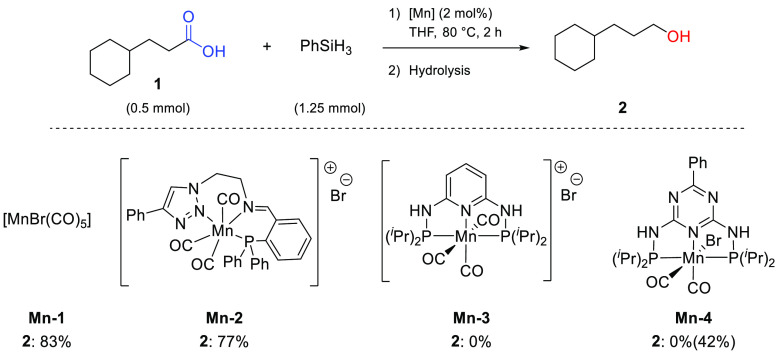
Screening of Manganese(I) Complexes for the Hydrosilylation of Cyclohexane Propanoic Acid Yields were determined after hydrolysis by NMR relative to the internal standard added before extraction. The yield obtained after base activation for **Mn-4** is given in the brackets (6 mol % KO^*t*^Bu).

Initially, we chose to study the catalytic performances of several manganese complexes for this transformation (Scheme 1): the commercial carbonyl complex [MnBr(CO)_5_] **Mn-1**, previously shown to be active for other hydrosilylation and hydrogenation reactions;^38,37,42^ the triazole PNN manganese complex **Mn-2**, recently reported in hydrosilylation reactions by our group;^32^ the complex **Mn-3**, whose ligand framework has been recently reported by Kirchner and co-workers for the hydrosilylation of carbon dioxide;^43^ and **Mn-4**, a manganese-triazine pincer complex active in hydrogenation reactions.^44^ The complexes were screened under identical conditions at a catalyst loading of 2 mol % with 2.5 equiv of PhSiH_3_ in THF at 80 °C; yields were determined by ^1^H NMR spectroscopy relative to an internal standard after hydrolysis (for details, see the Supporting Information). Among the pincer complexes, only the manganese triazole complex **Mn-2** showed activity for the hydrosilylation of carboxylic acid **1**, yielding 77% of the alcohol **2**. The PNP ligand frameworks induced no activity in either the cationic or neutral Mn(I) complexes. Only after activation with excess base did **Mn-4** convert the starting acid into 42% alcohol. Notably, the commercially available and bench-stable metal carbonyl complex [MnBr(CO)_5_] (**Mn-1**), bearing no additional controlling ligand, showed excellent performances, providing a high yield of 83% of alcohol **2** after hydrolysis.

Control experiments confirmed that no reaction took place under the screening conditions in the absence of a manganese complex. Even though there are reports of manganese(II) pincer complexes showing good activity in a range of other hydrosilylation reactions,^45−47^ manganese(II) and manganese(III) salts like MnCl_2_, MnBr_2_, Mn(OAc)_2_·(H_2_O)_4_, and Mn(OAc)_3_·(H_2_O)_2_ did not afford any product. The reaction could be carried out with good to excellent yields in a broad range of solvents, including even neat conditions (Table 1). In the perspective of balancing the solvent’s performance and environmental impact, 2-methyltetrahydrofuran (2-MTHF) was considered the preferred solvent of choice.^48^ While significant activity was also observed at lower temperatures, 80 °C was found to be appropriate, yielding 96% alcohol within the 2 h reaction time.

**Table 1:**
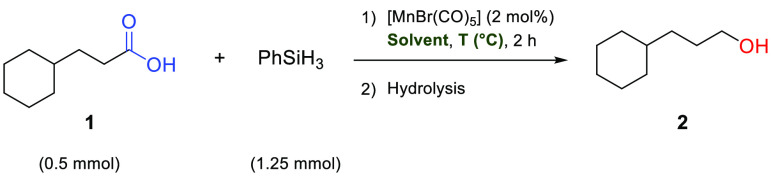
Solvent Selection and Temperature Variation for the Hydrosilylation of Cyclohexane Propanoic Acid (**1**) Using [MnBr(CO)_5_] (**Mn-1**) as the Catalyst

entry	solvent	temperature (°C)	yield (%)^a^
1	THF	80	83
2	neat	80	79
3	neat	100	87
4	toluene	80	92
5	heptane	80	92
6	cyclohexane	80	95
7	chloroform	80	>99
**8**	**2-MTHF**	**80**	**96**
9	2-MTHF	60	74
10	2-MTHF	40	38
11	2-MTHF	RT	3

a Yield was determined by NMR relative to ferrocene as the internal standard, added after hydrolysis.

Next, different silanes were tested in different stoichiometric amounts, and the best results for each silane are reported in Table 2. The sequential reduction of the number of hydrides and hydricity strength from the primary silane to the most sterically congested triphenyl silane, Ph_3_SiH, reduced the overall performance of the reaction. The oxygen-containing silanes, tetramethyldisiloxane (TMDS), and polymethylhydrosiloxane (PMHS) also performed poorly, making PhSiH_3_ the preferred choice.

**Table 2:**
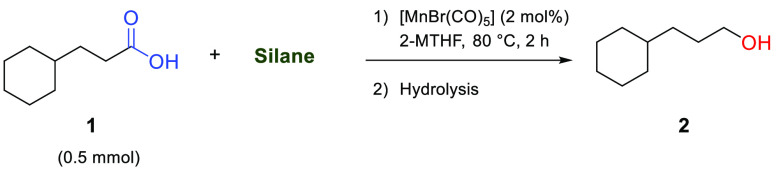
Silane Screening for the Hydrosilylation of Cyclohexane Propanoic Acid

entry	silane	quantity (mmol)	yield (%)^a^
1	PhSiH_3_	1.25	96
2	Ph_2_SiH_2_	3.0	33
3	Ph_3_SiH	1.25	0
4	TMDS	2.0	9
5	PMHS	5.0^b^	31

a Yields are the highest obtained for each silane, varying the silane equivalent (all reactions are listed in the Supporting Information).

b 10 mmol of monomeric units (MeOSiH) was used.

**Scheme 2:**
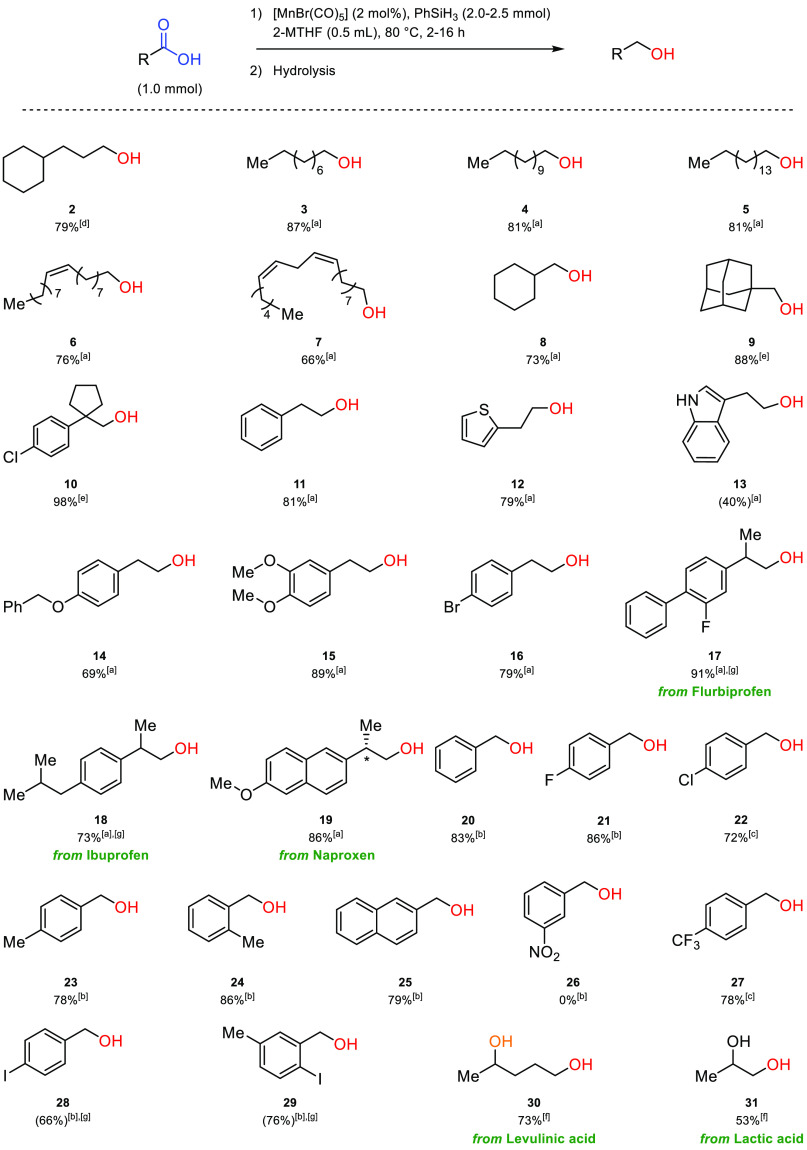
Scope of the Hydrosilylation Reaction of Aliphatic and Aromatic Carboxylic Acids to Their Respective Alcohols Isolated yields are given below each respective alcohol (NMR yield is given in the parentheses where applicable). [a] Standard conditions for the hydrosilylation of carboxylic acids: [MnBr(CO)_5_] (2 mol %), PhSiH_3_ (2.5 mmol), *T* = 80 °C, carboxylic acid (1 mmol), 2-MTHF (0.5 mL), time = 4 h. [b] Conditions for the hydrosilylation of most aromatic carboxylic acids: [MnBr(CO)_5_] (2 mol %), PhSiH_3_ (2.0 mmol), *T* = 80 °C, carboxylic acid (1 mmol), cyclohexane (0.5 mL), time = 2 h. [c] Same conditions as [b] using 2-MTHF (0.5 mL) as the solvent. [d] Time = 2 h; [e] time = 16 h. [f] Same conditions as [a] using PhSiH_3_ (3.0 mmol). [g] Half scale reaction.

The **Mn-1** catalyzed hydrosilylation with PhSiH_3_ in 2-MTHF was then applied to a scope of different carboxylic acids (Scheme 2). From the initial experiments, a silane to substrate ratio of 2.5:1 and a reaction time of 4 h was adopted for the aliphatic acids. The respective alcohols were obtained consistently in high yields across a wide range of substrates. Steric properties of the substrates seem to have little influence on the reaction performances: long-chain acids performed well under the reaction conditions yielding **3**–**5**, and the bulky adamantane carboxylic acid and the more complex substrate 1-(4-chlorophenyl)-1-cyclopentanecarboxylic acid gave **9** and **10** in very good yields at somewhat elongated reaction times. Interestingly, while [MnBr(CO)_5_] has been reported to be an active catalyst for the hydrosilylation of alkenes,^38^ the reaction proved highly selective for the carboxylic acid moiety, leaving the double bonds of oleic acid and linoleic acid intact to afford oleyl alcohol (**6**) and linoleyl alcohol (**7**) in good yields. For phenylacetic acid derivatives, the electronic nature and position of the ring substituents could be varied widely, and products **11** and **14**–**16** were all obtained in high yields. The reaction also tolerated some heterocycles: 2-thiopheneacetic acid was reduced to the corresponding alcohol **12** in high yields, while 3-indoleacetic acid was more troublesome, affording alcohol **13** in moderate yields. The reduction also performed well on some biologically active aryl propionic acids used as nonsteroidal anti-inflammatory drugs, such as flurbiprofen, ibuprofen, and naproxen, commercialized as the single enantiomer (*S*)-2-(6-methoxynaphthalen-2-yl)propanoic acid, which retained the stereochemistry at the chiral center.

For aromatic acids, excellent yields were also obtained, whereby some modifications of the conditions proved beneficial in individual cases (see the Supporting Information for details). Using a reaction time of 2 h and only 2 equiv of phenylsilane, 4-chlorobenzoic acid and electron-deficient 4-trifluoromethylbenzoic acid could be converted into their respective alcohols (**22**, **27**) in almost quantitative yields in 2-MTHF as the solvent. For other substrates, the less polar solvent cyclohexane proved superior, and a range of aromatic carboxylic acids, rarely reported in earth-abundant metal-catalyzed hydrosilylation reactions, could be reduced very effectively (Scheme 2). The alcohols **20**, **21**, **23**, and **24** were all obtained readily from benzoic acid, 4-fluorobenzoic acid, 4-methyl benzoic acid, and 2-methyl benzoic acid, respectively. Iodobenzoic acids such as 4-iodobenzoic acid and 2-iodo-5-methylbenzoic acid afforded alcohols **28** and **29** in good yields, for which notably no dehalogenation was observed. Likewise, 4-fluorobenzoic acid, 4-chlorobenzoic acid, and 4-bromo phenylacetic acid did not afford dehalogenated products.

In addition, the bioderived keto-acid, levulinic acid, was also selectively reduced to the respective diol **30** in good yield by the addition of three equivalents of phenylsilane without the formation of γ-valerolactone, the common alternative product obtained via hydrogenation of the acid.^49^ Finally, even the α-hydroxy-substituted lactic acid could be reduced to propylene glycol (**31**) in moderate yield in the same conditions. Unfortunately, 3-nitrobenzoic acid (bearing the nitro group, notoriously difficult in hydrosilylation reactions^28^) did not afford the desired product (**26**); 4-cyanobenzoic acid also did not afford any alcohol product, and the α,β-unsaturated trans-2-hexenoic acid led to a mixture of differently reduced and homocoupled products with no trace of the unsaturated alcohol. In addition, the *boc* protected *N*-(^*t*^butoxycarbonyl)-l-proline did not appear to be stable in the reaction conditions and did not allow for the recovery or the detection of either the *boc* group or the alcohol (more details in the Supporting Information).

**Figure 1:**
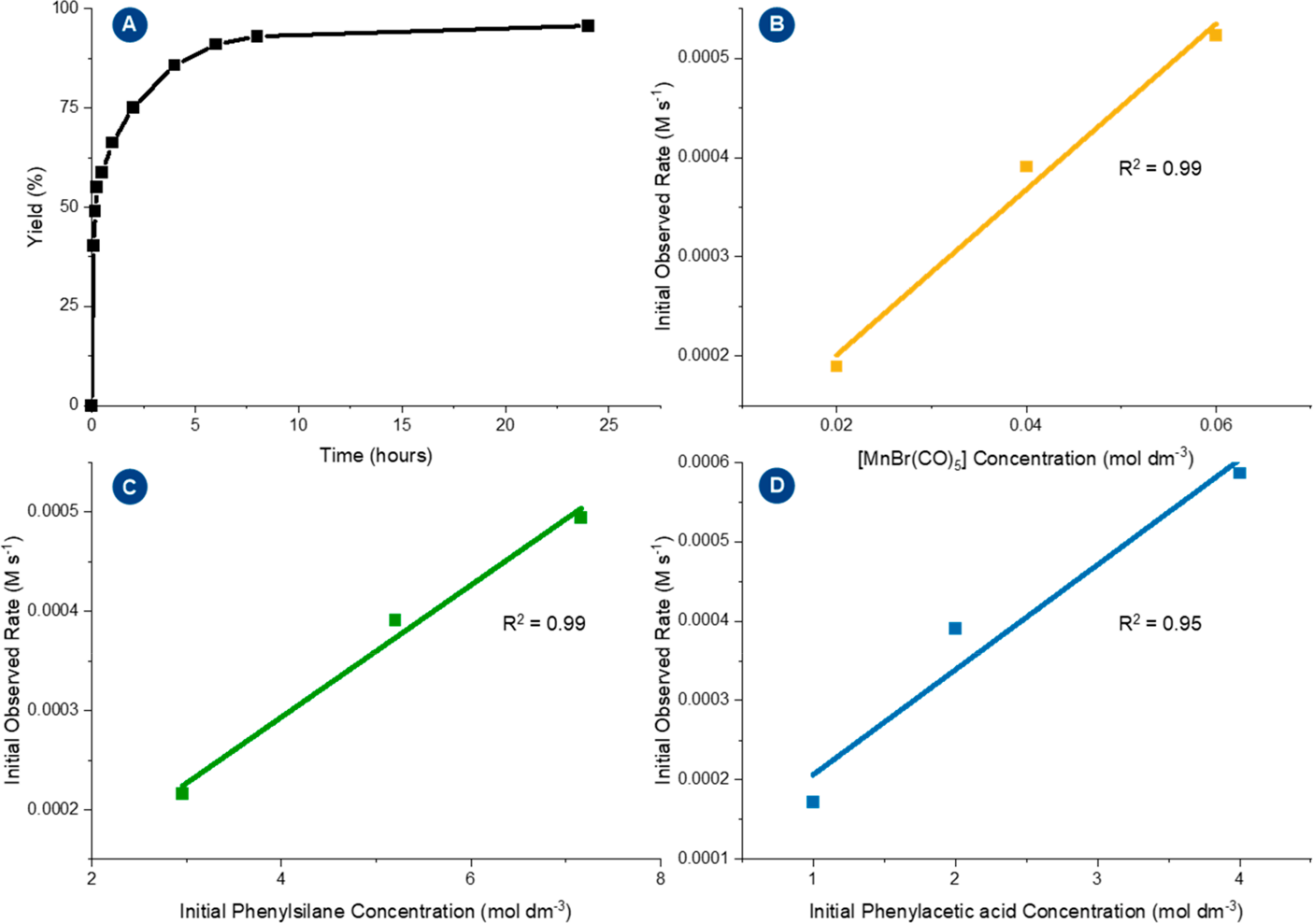
Panel A: yield–time profile performed on phenylacetic acid in conditions of the gram-scale reaction. Panel B: initial rates as a function of the concentration of catalyst [MnBr(CO)_5_]. Panel C: initial rates as a function of initial concentration of phenylsilane. Panel D: initial rates as a function of the concentration of phenylacetic acid. The initial rates were determined from the linear regression of four data points within the initial stages of the reaction (<25% yield; the full experimental procedure for the kinetic protocol is given in the Supporting Information).

To demonstrate the practical utility of the method, the reaction was performed on a preparative scale. Phenylacetic acid was used as a substrate for this reaction since 2-phenylethanol (**11**) is found in many naturally-based essential oils and is a common ingredient in the flavor and fragrance industry.^50^ We aimed to reduce the amount of silane as much as possible and rather compromise reaction time. Small scale hydrosilylation reactions with 1.0 and 0.7 equiv of silane showed no further conversion beyond 24 h, resulting in yields of only 55% and 20% of alcohol **11** even after 72 h. However, 1.5 equiv of PhSiH_3_ was found to be sufficient to reach the quantitative conversion of the acid within a reaction time of 24 h, as determined by monitoring the progression of the reaction in a yield/time profile (Figure 1, panel A). Under these improved reaction conditions ([MnBr(CO)_5_] (2 mol %), PhSiH_3_ (1.5 equiv), 6 mL of 2-MTHF, *T* = 80 °C, *t* = 24 h), 1.63 g of phenylacetic acid was converted successfully to give an isolated yield of 1.35 g (93%) of 2-phenyl ethanol (**11**). Continuing our studies on phenylacetic acid, we further explored the catalytic performance of [MnBr(CO)_5_] at lower catalyst loading. After a reaction time of 24 h in standard conditions, it was possible to obtain 2-phenyl ethanol in 78% yield with a catalyst loading as low as 0.5 mol %.

A further examination of the reduction of phenylacetic acid was then performed to establish the reaction order of the individual reactive components by the initial rates method (Figure 1, panels B–D). The temperature was reduced to 60 °C for the study to allow for a more accurate determination of yields as a function of time. A variation in the concentration of [MnBr(CO)_5_] while maintaining the same molar concentrations of phenylsilane and phenylacetic acid indicated an apparent first-order dependence of the reduction on the catalyst. A variation in the concentrations of phenylsilane and phenylacetic acid independently also showed linear correlations between the initial rate and concentration, indicating a first-order dependence with respect to both substrates. The same kinetic behavior has been reported previously for manganese catalyzed hydrosilylation reactions of ketones and formates.^51^

**Scheme 3:**
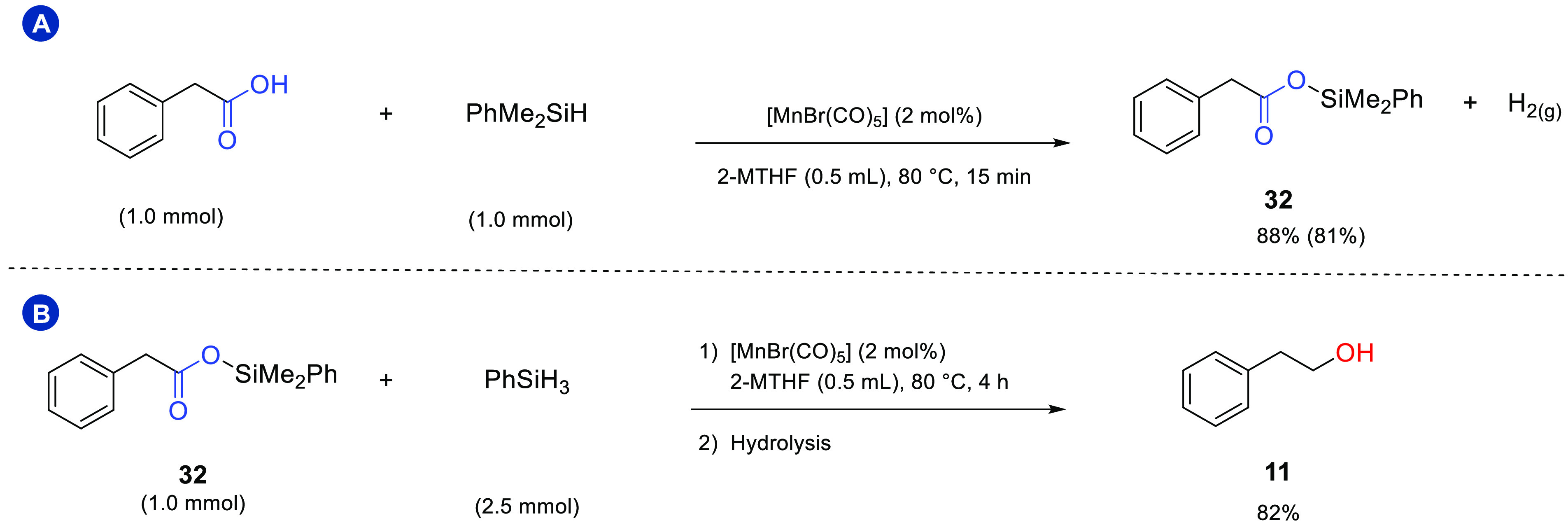
(A) Reaction between Phenylacetic Acid and Dimethylphenylsilane (Isolated Yield Is Given in Brackets); (B) Reaction of Silyl Ester Product **32** in the Same Conditions as Phenylacetic Acid

A vigorous gas evolution in the very early stage of the reaction indicated dehydrogenative coupling of PhSiH_3_ and the carboxylic acids to silyl esters PhSiH_*x*_(O_2_CR)_*y*_ as the initial reaction step.^20^ The evolved H_2_ was identified by GC-TCD analysis of the gas phase for phenylacetic acid as the substrate under standard conditions. The gaseous hydrogen does not contribute to the reduction as ascertained by releasing the atmosphere and flushing with argon immediately after its formation. Interestingly, even the notoriously less reactive tertiary silane PhMe_2_SiH^52,53^ reacted rapidly to give the corresponding silyl ester in 88% yield after 15 min. A blank reaction performed in the same conditions without [MnBr(CO)_5_] did not afford any product, showing the essential role of the Mn complex in catalyzing this step (Scheme 3, panel A). Notably, the tertiary silyl ester **32** was reduced smoothly with phenylsilane under standard conditions leading to 2-phenylethanol in 82% yield (Scheme 3, panel B). While this does not exclude H transfer occurring from PhSiH_*x*_(O_2_CR)_*y*_ as suggested previously,^20^ it demonstrates that PhSiH_3_ is able to reduce silyl ester intermediates in the presence of [MnBr(CO)_5_] as the catalyst.

**Scheme 4:**
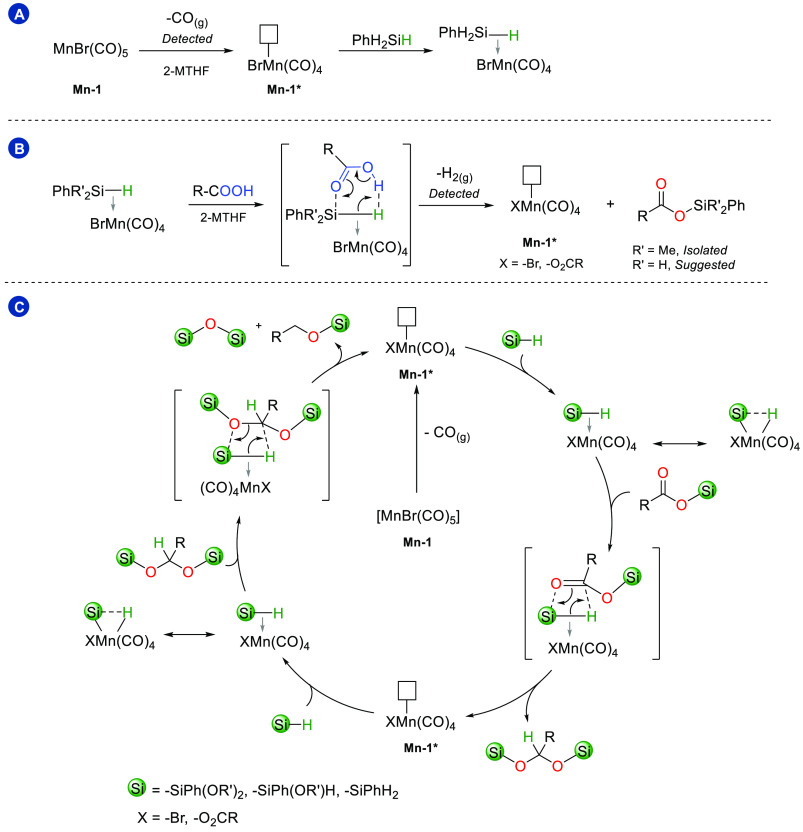
Tentative Reaction Mechanism Proposed in 3 Steps Panel A: Activation of [MnBr(CO)_5_] via CO detected dissociation in the presence of PhSiH_3_ and 2-MTHF as a solvent. Panel B: Formation of the silyl ester via detected H_2_ evolution. Panel C: Proposed catalytic cycle for the observed reduction of the silyl ester (Scheme 3, panel B) to afford the silylated alcohol and the silane-based byproducts (characterization in the Supporting Information).

The analysis of the gas phase composition of the reaction between phenylacetic acid and phenylsilane under standard conditions also revealed the presence of CO. A constant ratio of approximately 50:1 between H_2(g)_ and CO_(g)_ was determined after 15 min and 4 h. When it is assumed that the hydrogen evolved arises from the quantitative Si–O bond formation between the R–COOH and H–Si bonds of silane, the amount of CO accounts for one equivalent relative to the employed [MnBr(CO)_5_] complex. This suggests the dissociation of one CO ligand upon thermal activation to generate a 16-electron complex of type [MnX(CO)_4_] (X = bromide or carboxylate). The corroboration of catalytic activity with the formation of coordinatively unsaturated manganese carbonyl species is supported further by the ^31^P{^1^H}-NMR spectroscopic investigation of the reaction using the ligand modified complexes **Mn-2**, **Mn-3**, and **Mn-4** (see the Supporting Information). The spectra of the inactive complexes **Mn-3** and **Mn-4** revealed the signals of the pristine complex as major species after the typical reaction time. In contrast, the spectrum of the active complex **Mn-2** showed a signal at −16.1 ppm associated with a free PPh_2_ group, indicating ligand dissociation. The reported ability of unsaturated Mn(I) carbonyl complexes to activate Si–H bonds via sigma coordination^54^ suggests the possibility of a Mn(I) redox neutral Si–H bond activation circumventing the need for a Mn(I)/Mn(III) oxidative addition–reductive elimination process. An outer-sphere mechanistic proposal is suggested in Scheme 4.

## Conclusion

In conclusion, we have shown that the bench stable and commercially available manganese carbonyl complex [MnBr(CO)_5_] can catalyze the challenging reduction of a wide variety of both aryl and alkyl carboxylic acids to their respective alcohols in high yields via hydrosilylation. The scalable synthetic procedure can be carried out in standard laboratory glassware using benign solvents under mild conditions with only 1.5 equiv of phenylsilane, the lowest amount of silane reported to date for the hydrosilylation of carboxylic acids. Control experiments are consistent with the formation of silyl esters PhSiH_*x*_(O_2_CR)_*y*_ as intermediates and suggest a coordinatively unsaturated 16-electron Mn(I) carbonyl complex as the active species, probably involved in Si–H activation. Notably, this working hypothesis provides a rational explanation of the catalytic activity of [MnBr(CO)_5_] as compared to related pincer-type complexes due to the easier access of unsaturated species through ligand dissociation. Further investigations to elucidate the mechanistic details for hydride transfer at different Mn(I) coordinative frameworks are underway to broaden the catalytic potential of this earth-abundant and benign transition metal in homogeneous catalysis.

## Supporting Information Available

General considerations, experimental methods and synthetic details, and copies of NMR spectra (PDF)
