# Supplementary material for: Reduction of Carboxylic Acids to Alcohols via Manganese(I) Catalyzed Hydrosilylation
Source: JACS Au. 2021 May 11;1(6):742–9. doi: 10.1021/jacsau.1c00140 (PMC8395667; doi:10.1021/jacsau.1c00140)
Supplement: Supplementary file 1 — au1c00140_si_001.pdf [file au1c00140_si_001.pdf]

Supporting Information

Reduction of Carboxylic Acids to Alcohols via Manganese(I) Catalyzed Hydrosilylation

Emanuele Antico,[1,2,#] Peter Schlichter,[1,2,#] Christophe Werlé[1,3,*] and Walter Leitner[1,2]

[1] Max Planck Institute for Chemical Energy Conversion, Stiftstr. 34 – 36, 45470 Mülheim an der Ruhr, Germany.

[2] Institut für Technische und Makromolekulare Chemie (ITMC), RWTH Aachen University, Worringer Weg 2, 52074 Aachen, Germany.

[3] Ruhr University Bochum, Universitätsstr. 150, 44801 Bochum, Germany.

[#] These authors contributed equally to this work.

[*] Email: christophe.werle@cec.mpg.de

Table of Contents

1. General Considerations S3
2. Reaction Optimization S4
   2.1. General Method and Work-up Procedure S4
   2.2. Catalyst Screening and Control Experiments S5
   2.3. Solvent and Temperature Optimization S6
   2.4. $PhSiH_3$ Stoichiometry Screening S7
   2.5. Silane Screening S8
   2.6. Further Optimization on Benzoic Acid as Model Substrate for Aromatic Acids S9
3. Reaction Scope S10
   3.1. General Procedure (a) S10
   3.2. General Procedure (b) S11
   3.3. General Procedure (c) S12
4. Characterization Data S13
5. Preparative Scale Reaction S22
6. Yield Time Profile S23
7. Gas-phase Analysis Studies S25
   7.1. General Procedure S25
   7.2. Reactions Performed S25
8. Kinetic Experimentation S28

9. Mechanistic Studies S33
9.1. Synthesis of the Silyl Ester S33
9.2. Isolation of the Silyl Ester S34
9.3. Reaction of the Silyl Ester with $PhSiH_3$ S35
9.4. Hydrogen Release Reaction S36
9.5. $^{31}P\{^1H\}$ NMR Analysis of Crude Reaction Mixtures S36
10. Fate of the silane byproducts S41
10.1. Comment on the nature of the silane byproducts S41
10.2. Procedure for the ^1H-NMR of the crude reaction mixture prior to hydrolysis S47
10.3. Procedure for the $^{19}F\{^1H\}$ NMR of the crude reaction mixture prior to hydrolysis S47
10.4. Procedure for the isolation of **Si-3** S48
11. Chemoselectivity studies S49
11.1. The case of some disubstituted carboxylic acids S50
11.2. Amide substituted carboxylic acids S50
11.3. α,β-Unsaturated carboxylic acids S50
12. Low Catalyst Loading Experiments on Phenylacetic acid S51
13. NMR Spectra S53
14. References S85

1. General Considerations

Unless otherwise stated, all air-sensitive experiments were conducted under an argon atmosphere using standard Schlenk techniques or an MBraun inert-gas glovebox. Solvents for air- and moisture-sensitive experiments were purified through a MBraun-SPS-7 system or dried over activated 4Å molecular sieves and degassed according to standard laboratory procedure. Starting materials were purchased from Sigma Aldrich, Alfa Aesar, or TCI Europe and used as received. $[MnBr(CO)_5]$ has been used as received from Alfa Aesar. NMR spectra were recorded on Bruker AV-400 spectrometer. The coupling constants (*J*) are given in Hertz (Hz), and the chemical shifts (δ) expressed in ppm are calibrated using residual undeuterated solvent ($CHCl_3$, DMSO, THF-d_8 at 7.26, 2.50, 1.72 ppm for ^{1}H NMR, respectively, and 77.2, 39.5, 67.2 ppm for ^{13}C NMR, respectively). The peak patterns are indicated as follows: s = singlet; d = doublet; t = triplet; q = quartet; h = sextet: m = multiplet. Gas-phase analyses have been performed on a Shimadzu GC Nexis 2030 via manual injection (100 µL) equipped with a Restek Q-Bond column (Length: 30m; inner Diameter: 0.32 mm; film thickness: 10 µm) and a TCD detector using $He_{(g)}$ as a carrier gas. HR-MS have been recorded via an "LTQ-FT-Ultra" provided by Thermo Scientific. APCI-MS have been recorded on an "expression CMS" device provided by Advion. Optical rotations were measured with an A-Krüss Otronic Model P8000-t polarimeter at a wavelength of 589 nm. The values are given as specific optical rotation with exact temperature, concentration (c/(10 mg/mL)), and solvent.

2. Reaction Optimization

2.1. General Method and Work-up Procedure

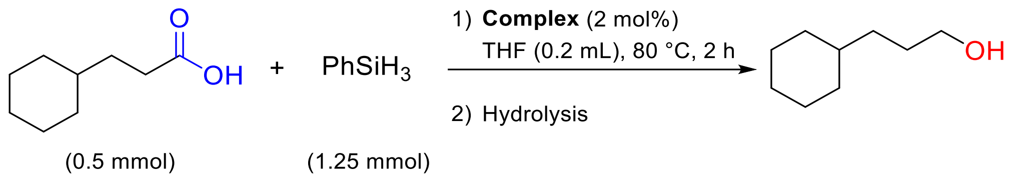

Scheme S1: Reaction between cyclohexane propanoic acid and $PhSiH_3$ in the presence of manganese catalyst.

The indicated complex (0.01 mmol) was placed into a Schlenk tube equipped with a screw cap and containing a magnetic stirrer. The reaction vessel was evacuated and filled with argon three times. Phenylsilane (135.3 mg, 1.25 mmol), cyclohexane propanoic acid (78.1 mg, 0.5 mmol), and THF (0.2 mL) were added under argon. The reaction mixture was heated at 80 °C for 2 hours. After cooling down the reaction vessel to room temperature, MeOH (3 mL) and an aqueous NaOH solution (10% w/w, 2 mL) were added dropwise. The resulting reaction mixture was stirred overnight. Before extraction, ferrocene (18.6 mg, 0.1 mmol) was dissolved in DCM (4 mL) and added as an internal NMR standard. The crude product was then extracted from the aqueous phase with DCM (3 × 10 mL). The combined organic layers were dried over $MgSO_4$, filtered, and concentrated under reduced pressure. Yields were determined by setting the integral value for the ferrocene singlet (4.16 ppm, 10H) as 1.00 and integrating the characteristic signal of the 3-Cyclohexyl-1-propanol (3.62 ppm, 2H).

$[MnBr(CO)_5]$ has been used as received from Alfa Aesar, while complexes **Mn-2**,[1] **Mn-3**,[2] and **Mn-4**[3] have been synthesized according to reported procedures. The spectroscopic data for these complexes is consistent with what has been previously reported.

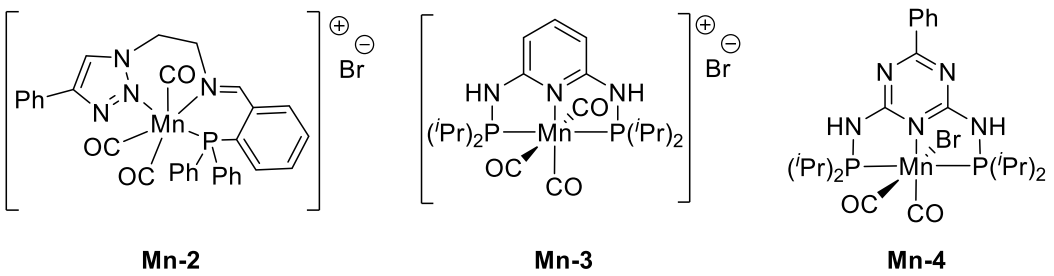

Scheme S2: Scheme of the manganese complexes.

Table S1 – Screening of different Manganese(I) complexes for the hydrosilylation of cyclohexane propanoic acid.[a]Average yield taken from two reaction runs. [b]Solid Gel – within the first five minutes, the reaction mixture would turn into a complete solid gel preventing mixing/stirring. The gel could not be dissolved by organic solvent, but it could be hydrolyzed using a NaOH solution.

Entry	Complex (2 mol%)	Yield (%)
1	$[MnBr(CO)_5]$	83[a]
2	**Mn-2**	77
3	**Mn-3**	<5
4	**Mn-3** + 6 mol% KO^{t}Bu	Solid gel[b]
5	**Mn-4**	0
6	**Mn-4** + 6 mol% KO^{t}Bu	42

2.2. Catalyst Screening and Control Experiments

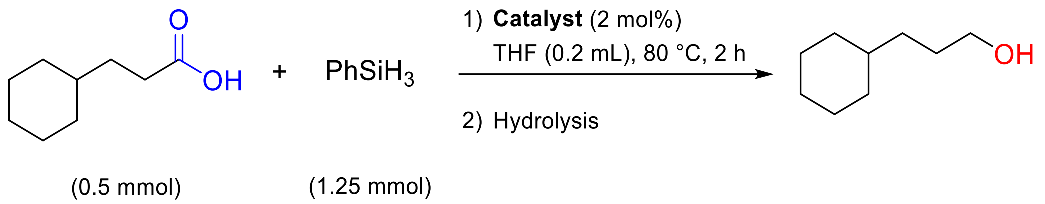

Scheme S3: Reaction between cyclohexane propanoic acid and $PhSiH_3$ in the presence of manganese catalyst.

Table S2 – Testing the catalytic activity of different manganese salts and other compounds for the hydrosilylation of cyclohexane propanoic acid. [a] 6 mol% of KO^{t}Bu was tested.

Entry	Catalyst (2 mol%)	Yield (%)
1	$[MnBr(CO)_5]$	83
2	$MnCl_2$	0
3	$MnBr_2$	0
4	$Mn(acetate)_2$	0
5	$Mn(acetate)_3$	Trace
6	Tetrabutylammonium bromide	0
7	KO^{t}Bu[a]	Solid Gel[a]
8	No Catalyst	0

[a]Eventual presence of the desired alcohol has not been investigated

2.3. Solvent and Temperature Optimization

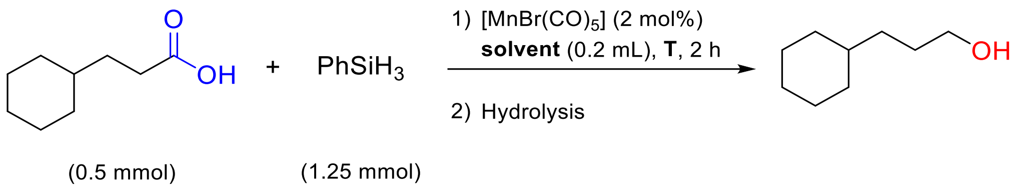

Scheme S4: Reaction between cyclohexane propanoic acid and $PhSiH_3$ in the presence of manganese catalyst.

Table S3 – Solvent and temperature screening for the hydrosilylation of cyclohexane propanoic acid, using $[MnBr(CO)_5]$ as a catalyst.

**Solvent**	**T (°C)**	**Yield (%)**
Neat	100	87
2-MTHF	80	96
Toluene	80	92
Heptane	80	92
Cyclohexane	80	95
$CHCl_3$	80	>99
2-MTHF	60	74
2-MTHF	40	38
Neat	r.t.	7
Toluene	r.t.	5
Heptane	r.t.	6
Cyclohexane	r.t.	7
$CHCl_3$	r.t.	8
THF	r.t.	0
2-MTHF	r.t.	3

2.4. $PhSiH_3$ Stoichiometry Screening

- **2-hour Reaction on Cyclohexane Propanoic Acid**

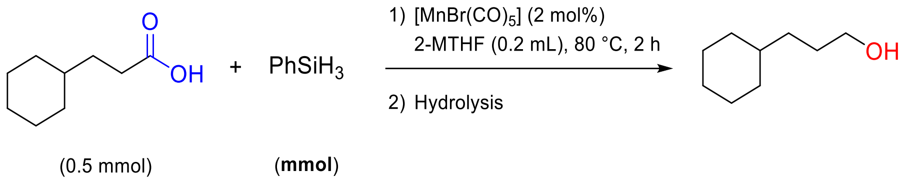

Scheme S5: Reaction between cyclohexane propanoic acid and $PhSiH_3$ in the presence of manganese catalyst.

Table S4 – Varying amounts of phenylsilane were trialed to determine the optimized conditions for the reaction.

$PhSiH_3$ (mmol)	Yield (%)
1.25	96
1.00	89
0.75	82
0.50	59

- **Time and minimum $PhSiH_3$ loading screening on phenyl acetic acid.**

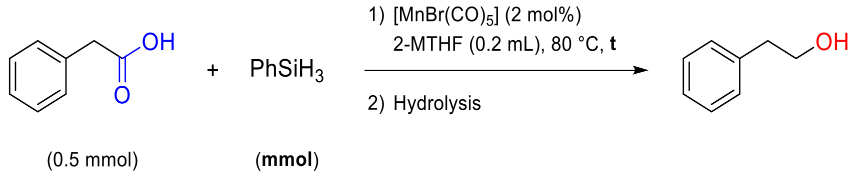

Scheme S6: Reaction between phenylacetic acid and $PhSiH_3$ in the presence of manganese catalyst.

Table S5 – Attempt to minimize $PhSiH_3$ amount by screening longer reaction times.

Time (h)	$PhSiH_3$ (mmol)	Yield (%)
24	0.75	96
24	0.50	59
24	0.35	25
72	0.50	55
72	0.35	20

This optimization was performed for the gram-scale reaction reported in section: 5. Preparative Scale Reaction.

2.5. Silane Screening

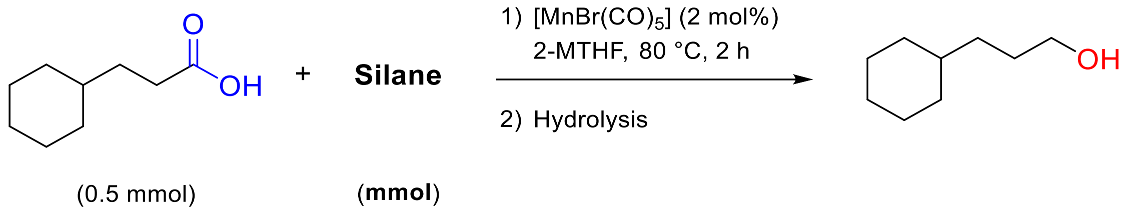

Scheme S7: Reaction between cyclohexane propanoic acid and different silanes in the presence of manganese catalyst.

Table S6 – various silane screening for the hydrosilylation of cyclohexane propanoic acid using $[MnBr(CO)_5]$ as a catalyst. Silanes bearing fewer Si-H bonds with respect to $PhSiH_3$ were also screened, increasing their loading. [a] 0.5 mL of solvent was added. [b] 1 mL of solvent was added, and reaction time was also extended to 4 hours. [c] 2 mL of solvent was added. [d] mmol of monomeric units (MeOSiH) of PMHS are given.

Silane	mmol	Yield (%)
$PhSiH_3$	1.25	96
Ph_2SiH_2	1.25	30
Ph_2SiH_2	3.0	33
Ph_3SiH	1.25	0
TMDS	2.0	9
PMHS[d]	3.0	Solid Gel
PMHS[d]	4.0[a]	Solid Gel
PMHS[d]	4.0[b]	29
PMHS[d]	5.0[c]	31

2.6. Further Optimization on Benzoic Acid as Model Substrate for Aromatic Acids

- **Solvent Screening**

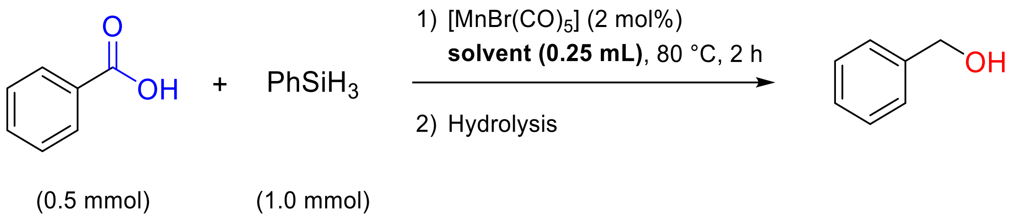

Scheme S8: Reaction between phenylacetic acid and $PhSiH_3$ in the presence of manganese catalyst.

Table S7 – Further solvent screening for the hydrosilylation of benzoic acid.

Solvent	Yield %
2-MTHF	77
Toluene	99
Cyclohexane	98
Heptane	93

- **$PhSiH_3$ Stoichiometry Screening**

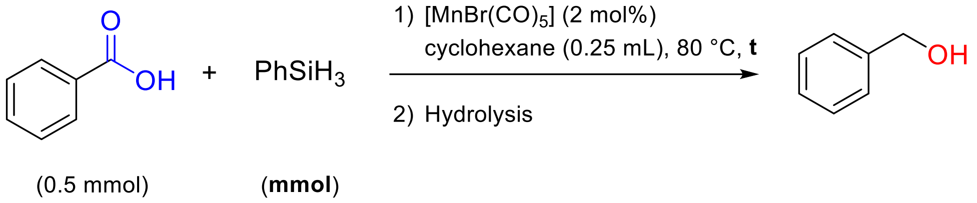

Scheme S9: Reaction between phenylacetic acid and $PhSiH_3$ in the presence of manganese catalyst.

Table S8 – time and phenylsilane equivalents are screened for the hydrosilylation of cyclohexane propanoic acid in cyclohexane.

Time (h)	$PhSiH_3$ (mmol)	Yield %
2	1.00	98
2	0.75	83
4	0.75	94

3. Reaction Scope

3.1. General Procedure (a)

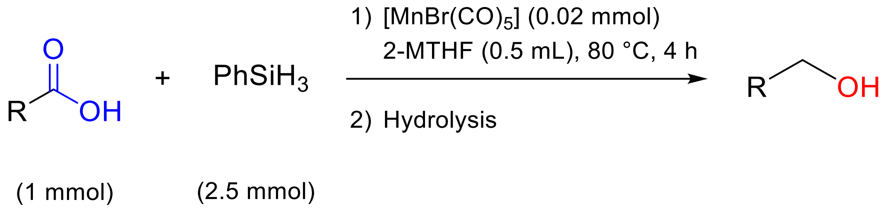

Scheme S10: Reaction between acids and $PhSiH_3$ in the presence of manganese catalyst.

- **For Liquid Carboxylic Acids**

$[MnBr(CO)_5]$ (5.5 mg, 0.02 mmol) was placed into a Schlenk tube equipped with a screw cap and containing a magnetic stirrer. The reaction vessel was evacuated and filled with argon three times. Phenylsilane (270.5 mg, 2.5 mmol), carboxylic acid (1.0 mmol), and 2-MTHF (0.5 mL) were added under argon. The mixture was heated at 80 °C for 4 hours. After cooling down the reaction vessel to room temperature, MeOH (6 mL) and an aqueous NaOH solution (10% w/w, 4 mL) were added dropwise. Then, the resulting reaction mixture was stirred overnight. Before extraction, ferrocene (37.2 mg, 0.2 mmol) was dissolved in DCM (4 mL) and added as an internal. The crude product was then extracted from the aqueous phase with DCM (3 × 10 mL). The combined organic layers were dried over $MgSO_4$, filtered, and the solvents removed under reduced pressure. NMR yields were determined by setting the integral value for the ferrocene singlet (4.16 ppm, 10H) as 1.00 and integrating the characteristic methylene R-CH_2-OH signal of the respective product (typically ≈3.5 ppm, 2H). Products for isolated yields were purified through a bulb to bulb distillation under reduced pressure (Kugelrohr distillation).

- **For Solid Carboxylic Acids**

$[MnBr(CO)_5]$ (5.5 mg, 0.02 mmol) and the carboxylic acid (1 mmol) were placed into a Schlenk tube equipped with a screw cap and containing a magnetic stirrer. The reaction vessel was evacuated and filled with argon three times. Phenylsilane (270.5 mg, 2.5 mmol) and 2-MTHF (0.5 mL) were added under argon. The mixture was heated at 80 °C for 4 hours. After cooling down the reaction vessel to room temperature, MeOH (6 mL) and an aqueous NaOH solution (10% w/w, 4 mL) were added dropwise. Then, the resulting reaction mixture was stirred overnight. Before extraction, ferrocene (37.2 mg, 0.2 mmol) was dissolved in DCM (4 mL) and added as an internal. The crude product was then extracted from the aqueous phase with DCM (3 × 10 mL). The combined organic layers were dried over $MgSO_4$, filtered, and the solvents removed

under reduced pressure. NMR yields were determined by setting the integral value for the ferrocene singlet (4.16 ppm, 10H) as 1.00 and integrating the characteristic methylene R-CH_2-OH signal of the respective product (typically ≈3.5 ppm, 2H). Products for isolated yields were purified through a bulb to bulb distillation under vacuum (Kugelrohr distillation). Naproxen, Flurbiprofen, and Ibuprofen were purified via column chromatography (elution mixture used is given together with NMR data).

3.2. General Procedure (b)

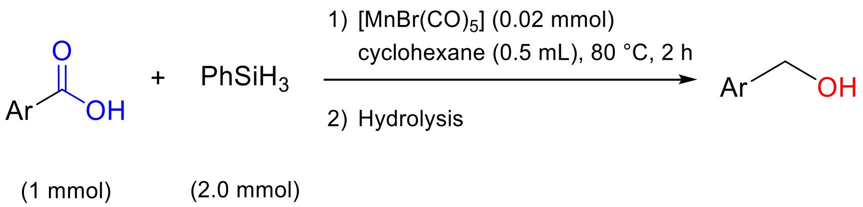

Scheme S11: Reaction between carboxylic acids and $PhSiH_3$ in the presence of manganese catalyst.

[MnBr(CO)$_5$] (5.5 mg, 0.02 mmol) and the carboxylic acid (1 mmol) were placed into a Schlenk tube equipped with a screw cap and containing a magnetic stirrer. The reaction vessel was evacuated and filled with argon three times. Phenylsilane (216.4 mg, 2.0 mmol) and cyclohexane (0.5 mL) were added under argon. The mixture was heated at 80 °C for 2 hours. After cooling down the reaction vessel to room temperature, MeOH (6 mL) and an aqueous NaOH solution (10% w/w, 4 mL) were added dropwise. Then, the resulting reaction mixture was stirred overnight. Before extraction, ferrocene (37.2 mg, 0.2 mmol) was dissolved in DCM (4 mL) and added as an internal. The crude product was then extracted from the aqueous phase with DCM (3 × 10 mL). The combined organic layers were dried over $MgSO_4$, filtered, and the solvents removed under reduced pressure. NMR yields were obtained by setting the integral value for the ferrocene singlet (4.16 ppm, 10H) as 1.00 and integrating the characteristic methylene Ar-CH_2-OH signal of the respective product (typically ≈ 4.7 ppm, 2H). Products for isolated yields were purified through a bulb to bulb distillation under vacuum (Kugelrohr distillation). When necessary, solid alcohols (e.g., 4-Methylbenzyl alcohol, 2-Naphtalene methanol, 2-Methylbenzyl alcohol) were washed after the distillation process with pentane at -25 °C.

3.3. General Procedure (c)

- **For Levulinic acid and Lactic acid**[1]

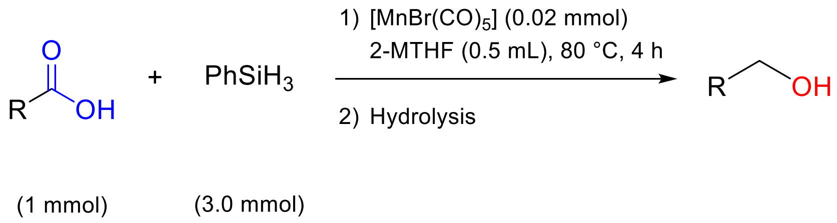

Scheme S12: Reaction between carboxylic acids and $PhSiH_3$ in the presence of manganese catalyst.

$[MnBr(CO)_5]$ (5.5 mg, 0.02 mmol) was placed into a Schlenk tube equipped with a screw cap and containing a magnetic stirrer. The reaction vessel was evacuated and filled with argon three times. Carboxylic acid (1.0 mmol), phenylsilane (324.7 mg, 3.0 mmol), and 2-MTHF (0.5 mL) were added under argon. The mixture was heated at 80 °C for 4 hours. After cooling down the reaction vessel to room temperature, a saturated NaOH solution in MeOH (0.5 mL), MeOH (0.5 mL), and chloroform (4 mL) were added dropwise. Then, the resulting reaction mixture was stirred overnight. The crude product was loaded onto a silica column before being washed with chloroform or dichloromethane (100 mL). The product was then eluted with isopropanol and purified through a bulb to bulb distillation under vacuum (Kugelrohr distillation).

[1] Hydroxy groups may develop $H_{2(g)}$ in reaction conditions. Consider when preparing appropriate experimental set-up.

4. Characterization Data

All spectra of previously existent substances are consistent with reported NMR spectra.

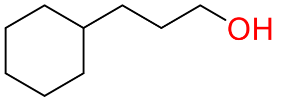

Cyclohexane propanol **(2)**[4]

$C_9H_{18}O$, colorless oil.

^{1}H NMR (400 MHz, $CDCl_3$, 296 K) δ 3.62 (t, *J* = 6.7 Hz, 2H), 1.77 – 1.49 (m, 7H), 1.31 (s, 1H), 1.26 – 1.10 (m, 6H), 0.96 – 0.80 (m, 2H).

^{13}C{^{1}H} NMR (101 MHz, $CDCl_3$, 296 K) δ 63.6, 37.6, 33.6, 33.5 (2C), 30.3, 26.8, 26.5 (2C).

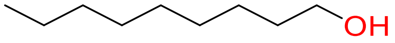

Nonanol **(3)**[5]

$C_9H_{20}O$, colorless oil.

^{1}H NMR (400 MHz, $CDCl_3$, 296 K) δ 3.63 (t, *J* = 6.7 Hz, 2H), 1.61-1.52 (m, 2H), 1.39 – 1.17 (m, 13 H), 0.88 (t, *J* = 6.7 Hz, 3H).

^{13}C{^{1}H} NMR (101 MHz, $CDCl_3$, 296 K) δ 63.3, 32.9, 32.0, 29.7, 29.6, 29.4, 25.9, 22.8, 14.3.

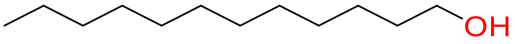

Lauryl alcohol **(4)**[6]

$C_{12}H_{26}O$, colorless oil.

^{1}H NMR (400 MHz, $CDCl_3$, 296 K) δ 3.64 (t, *J* = 6.6 Hz, 2H), 1.62 – 1.51 (m, 2H), 1.38 **-** 1.18 (m, 19H), 0.88 (t, *J* = 6.8 Hz, 3H).

^{13}C{^{1}H} NMR (101 MHz, $CDCl_3$, 296 K) δ 63.3, 32.4, 32.1, 29.8, 29.8, 29.8, 29.8, 29.6, 29.5, 25.9, 22.8, 14.3.

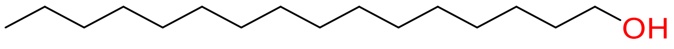

Cetyl alcohol **(5)**[6]

$C_{16}H_{34}O$, white solid.

^{1}H NMR (400 MHz, $CDCl_3$, 296 K) δ 3.67 – 3.60 (t, *J* = 6.4, 2H), 1.60-1.52 (m, 2H), 1.39-1.18 (m, 27H), 0.88 (t, *J* = 6.5 Hz, 3H).

^{13}C{^{1}H} NMR (101 MHz, $CDCl_3$, 297 K) δ 63.2, 32.9, 32.1, 29.8 (4C), 29.8 (2C), 29.8, 29.8, 29.6, 29.5, 25.9, 22.8, 14.3.

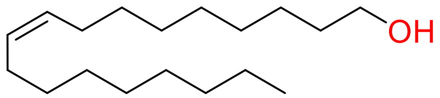

Oleyl alcohol **(6)**[6]

$C_{18}H_{36}O$, colorless oil.

^{1}H NMR (400 MHz, $CDCl_3$, 296 K) δ 5.37 – 5.32 (m, 2H), 3.64 (t, *J* = 6.6 Hz, 2H), 2.02 (m, 4H), 1.65 – 1.48 (m, 2H), 1.39-1.20 (m, 23H), 0.93 – 0.78 (t, *J* = 6.6 Hz, 3H).

^{13}C{^{1}H} NMR (101 MHz, $CDCl_3$, 296 K) δ 130.0, 129.9, 63.3, 32.9, 32.1, 29.9, 29.9, 29.6, 29.6, 29.6, 29.5 (2C), 29.4, 27.4, 27.4, 25.6, 22.8, 14.3.

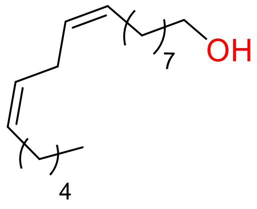

Linoleyl alcohol **(7)**[7]

$C_{18}H_{34}O$, colorless oil.

^{1}H NMR (400 MHz, $CDCl_3$, 296 K) δ 5.50 – 5.27 (m, 4H), 3.72 – 3.57 (q, *J* = 6.8 Hz, 2H), 2.77 (t, *J* = 6.5 Hz, 2H), 2.05 (q, *J* = 6.8 Hz, 4H), 1.56 (m, 2H), 1.32 (m, 17H), 0.89 (t, *J* = 6.8 Hz, 3H).

^{13}C{^{1}H} NMR (101 MHz, $CDCl_3$, 296 K) δ 130.3, 130.3, 128.1, 128.1, 63.2, 32.9, 31.7, 29.8, 29.6, 29.5, 29.5, 29.4, 27.4, 27.4, 25.9, 25.8, 22.7, 14.2.

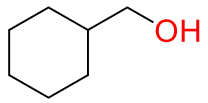

Cyclohexane methanol **(8)**[8]

$C_7H_{14}O$, colorless oil.

^{1}H NMR (400 MHz, $CDCl_3$, 296 K) δ 3.44 (d, *J* = 3.6 Hz, 2H), 1.73-1.63 (m, 5H), 1.53-1.42 (m, 1H), 1.32-1.09 (m, 4H), 0.99-0.87 (m, 2H).

$^{13}C\{^1H\}$ NMR (101 MHz, $CDCl_3$, 296 K) δ 68.9, 40.6, 29.7 (2C), 26.7, 25.9 (2C).

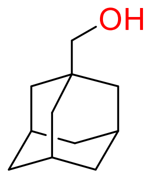

1-Adamantane methanol **(9)**[9]

$C_{11}H_{18}O$, white solid.

^{1}H NMR (400 MHz, $CDCl_3$, 296 K) δ 3.20 (d, *J* = 5.3 Hz, 2H), 1.99 (s, 3H), 1.77 – 1.61 (m, 6H), 1.51 (d, *J* = 2.2 Hz, 6H), 1.25 (br s, 1H).

$^{13}C\{^1H\}$ NMR (101 MHz, $CDCl_3$, 296 K) δ 74.0, 39.2 (3C), 37.3 (3C), 34.6, 28.3 (3C).

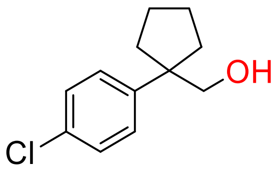

1-(4-chlorophenyl)-1-cyclopentane methanol **(10)**[10]

$C_{12}H_{15}ClO$, white solid.

^{1}H NMR (400 MHz, $CDCl_3$, 296 K) δ 7.35 – 7.19 (m, 4H), 3.51 (d, *J* = 6.2 Hz, 2H), 2.00 (m, 2H), 1.83 (m, 2H), 1.73 (m, 4H), 1.20 (s, 1H).

$^{13}C\{^1H\}$ NMR (101 MHz, $CDCl_3$, 296 K) δ 145.5, 132.1, 128.9 (2C), 128.5(2C), 70.2, 53.1, 34.5 (2C), 23.9 (2C).

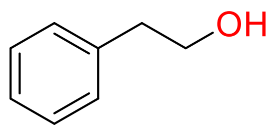

2-Phenylethanol **(11)**[8]

$C_8H_{10}O$, colorless oil.

^{1}H NMR (400 MHz, $CDCl_3$, 296 K) δ 7.37 – 7.29 (m, 2H), 7.24 (m, 3H), 3.87 (q, *J* = 5.9 Hz, 2H), 2.88 (t, *J* = 6.6 Hz, 2H), 1.42 (s, 1H).

^{13}C{^{1}H} NMR (101 MHz, $CDCl_3$, 296 K) δ 138.6, 129.2 (2C), 128.7 (2C), 126.6, 63.8, 39.3.

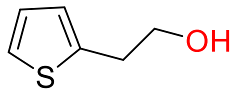

2-Thiopheneethanol **(12)**[11]

C_6H_8OS, colorless oil.

^{1}H NMR (400 MHz, $CDCl_3$, 296 K) δ 7.18 (dd, *J* = 5.1, 1.2 Hz, 1H), 6.96 (dd, *J* = 5.1, 3.4 Hz, 1H), 6.88 (m, 1H), 3.87 (t, *J* = 6.3 Hz, 2H), 3.09 (t, *J* = 5.8 Hz, 2H), 1.58 (s, 1H).

^{13}C{^{1}H} NMR (101 MHz, $CDCl_3$, 296 K) δ 140.9, 127.1, 125.8, 124.2, 63.7, 33.4.

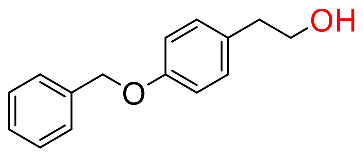

(4-Benzyloxyphenyl)ethanol **(14)**[12]

$C_{15}H_{16}O_2$, white solid.

^{1}H NMR (400 MHz, $CDCl_3$, 296 K) δ 7.48 – 7.29 (m, 5H), 7.15 (d, *J* = 8.6 Hz, 2H), 6.94 (d, *J* = 8.6 Hz, 2H), 5.05 (s, 2H), 3.83 (q, *J* = 6.4 Hz, 2H), 2.82 (t, *J* = 6.5 Hz, 2H), 1.36 (t, *J* = 6.0 Hz, 1H).

^{13}C{^{1}H} NMR (101 MHz, $CDCl_3$, 296 K) δ 157.7, 137.2, 130.8, 130.1 (2C), 128.7 (2C), 128.1, 127.6 (2C), 115.2 (2C), 70.2, 63.9, 38.4.

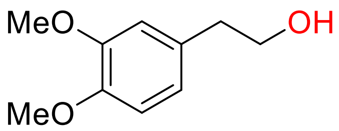

3,4-dimethoxy-Benzyl ethanol **(15)**[13]

$C_{10}H_{14}O_3$, colorless oil.

^{1}H NMR (400 MHz, CDCl$_3$, 296 K) δ 6.83 (d, *J* = 8.0 Hz, 1H), 6.80 – 6.74 (m, 2H), 3.91 – 3.80 (m, 8H), 2.82 (t, *J* = 6.5 Hz, 2H), 1.41 (s, 1H).

^{13}C{^{1}H} NMR (101 MHz, CDCl$_3$, 297 K) δ 149.1, 147.8, 131.1, 121.1, 112.3, 111.5, 63.9, 56.0, 55.9, 38.9.

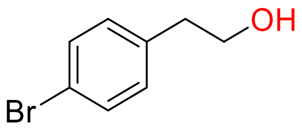

4-bromophenyl ethanol **(16)**[14]

C_8H_9OBr, colorless oil.

^{1}H NMR (400 MHz, CDCl$_3$, 296 K) δ 7.43 (d, *J* = 8.3 Hz, 2H), 7.11 (d, *J* = 8.3 Hz, 2H), 3.85 (q, *J* = 6.0 Hz, 2H), 2.83 (t, *J* = 6.5 Hz, 2H), 1.38 (s, 1H).

^{13}C{^{1}H} NMR (101 MHz, CDCl$_3$, 297 K) δ 137.7, 131.7 (2C), 130.9 (2C), 120.4, 63.5, 38.7.

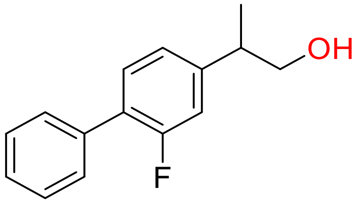

Flurbiprofen alcohol **(17)**[15]

$C_{15}H_{15}OF$, white solid. This compound was purified from the crude by column chromatography, using an elution mixture of 4:1 Pentane: Ethyl Acetate.

^{1}H NMR (400 MHz, CDCl$_3$, 296 K) δ 7.57 – 7.51 (m, 2H), 7.48 – 7.32 (m, 4H), 7.15 – 7.01 (m, 2H), 3.75 (t, *J* = 6.3 Hz, 2H), 3.00 (h, *J* = 6.9 Hz, 1H), 1.38 (t, *J* = 6.1 Hz, 1H), 1.31 (d, *J* = 7.0 Hz, 3H).

^{13}C{^{1}H} NMR (101 MHz, CDCl$_3$, 296 K) δ 160.0 (d, *J* = 248.1 Hz), 145.7 (d, *J* = 7.2 Hz), 135.8, 130.9 (d, *J* = 4.0 Hz), 129.1 (d, *J* = 2.9 Hz, 2C), 128.6 (2C), 127.7, 127.4 (d, *J* = 13.5 Hz), 123.7 (d, *J* = 3.2 Hz), 115.1 (d, *J* = 22.8 Hz), 68.6, 42.2 (d, *J* = 1.5 Hz), 17.6.

^{19}F{^{1}H} NMR (376 MHz, CDCl$_3$, 296 K) δ 117.9.

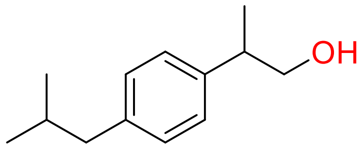

Ibuprofen alcohol **(18)**[15]

$C_{13}H_{20}O$, colorless oil.

This compound was purified from the crude by column chromatography, using an elution mixture of 15:1 Pentane: Ethyl Acetate.

^{1}H NMR (400 MHz, $CDCl_3$, 296 K) δ 7.13 (q, J = 8.2 Hz, 4H), 3.69 (d, J = 6.8 Hz, 2H), 2.93 (h, J = 7.0 Hz, 1H), 2.45 (d, J = 7.2 Hz, 2H), 1.85 (m, 1H), 1.30 (s, 1H), 1.27 (d, J = 7.0 Hz, 3H), 0.90 (d, J = 6.7 Hz, 6H).

^{13}C{^{1}H} NMR (101 MHz, $CDCl_3$, 296 K) δ 140.7, 140.1, 129.4 (2C), 127.2 (2C), 68.8, 45.1, 42.1, 30.2, 22.4 (2C), 17.6.

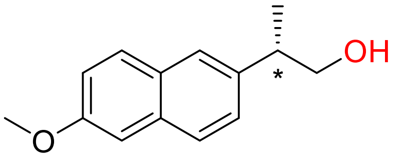

Naproxen alcohol **(19)**[15]

$C_{14}H_{16}O_2$, white solid.

This compound was purified from the crude by column chromatography, using an elution mixture of 3:1 Pentane: Ethyl Acetate. $[\alpha]_D^{20}$ = –18.7 (c = 1, $CHCl_3$). $[\alpha]_D^{25}$ = –18.5 (c = 1, $CHCl_3$). The specific optical rotation values obtained for (S)-Naproxen alcohol at the given temperatures and concentration, are in agreement with what reported in the literature[16].

^{1}H NMR (400 MHz, $CDCl_3$, 296 K) δ 7.71 (dd, J = 8.5, 7.0 Hz, 2H), 7.61 (d, J = 1.8 Hz, 1H), 7.35 (dd, J = 8.5, 1.8 Hz, 1H), 7.17 – 7.10 (m, 2H), 3.92 (s, 3H), 3.78 (t, J = 6.4 Hz, 2H), 3.09 (h, J = 6.9 Hz, 1H), 1.36 (d, J = 7.0 Hz, 3H).

^{13}C{^{1}H} NMR (101 MHz, $CDCl_3$, 296K) δ 157.5, 138.6, 133. 6, 129.1, 129.1, 127.3, 126.3, 125.93, 118.9, 105.6, 68.7, 55.3, 42.4, 17.7.

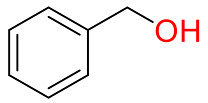

Benzyl alcohol **(20)**[8]

C_7H_8O, colorless oil.

^{1}H NMR (400 MHz, $CDCl_3$, 296 K) δ 7.40 – 7.35 (d, 4.7 Hz, 4H), 7.34 – 7.27 (m, 1H), 4.71 (d, *J* = 5.7 Hz 2H), 1.68 – 1.60 (m, 1H).

^{13}C{^{1}H} NMR (101 MHz, $CDCl_3$, 296 K) δ 140.9, 128.5 (2C), 127.6, 127.0 (2C), 65.1.

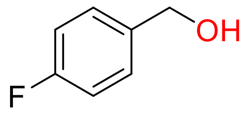

4-fluoro-Benzyl alcohol **(21)**[6]

C_7H_7OF, colorless oil.

^{1}H NMR (400 MHz, $CDCl_3$, 296 K) δ 7.36 – 7.30 (m, 2H), 7.09 – 7.00 (m, 2H), 4.66 (d, *J* = 5.6 Hz, 2H), 1.80 – 1.66 (m, 1H).

^{13}C{^{1}H} NMR (101 MHz, $CDCl_3$, 296 K) δ 162.4 (d, *J* = 245.5 Hz), 136.7 (d, *J* = 3.1 Hz), 128.9 (d, *J* = 8.0 Hz, 2C), 115.5 (d, *J* = 21.4 Hz, 2C), 64.8.

^{19}F{^{1}H} NMR (376 MHz, $CDCl_3$, 296 K) δ 114.9.

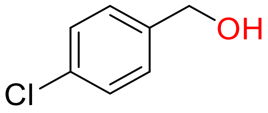

4-chloro-Benzyl alcohol **(22)**[8]

C_7H_7OCl, white solid.

^{1}H NMR (400 MHz, $CDCl_3$, 296 K) δ 7.36 – 7.28 (m, 4H), 4.68 (d, *J* = 5.6 Hz, 2H), 1.65 (t, *J* = 5.8 Hz, 1H).

^{13}C{^{1}H} NMR (101 MHz, $CDCl_3$, 297 K) δ 139.4, 133.4, 128.8 (2C), 128.4 (2C), 64.5.

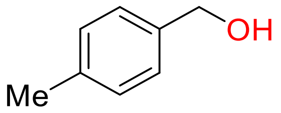

4-methyl-Benzenemethanol **(23)**[17]

$C_8H_{10}O$, white solid.

^{1}H NMR (400 MHz, $CDCl_3$, 296 K) δ 7.26 (d, *J* = 8.0 Hz, 2H), 7.18 (d, *J* = 7.8 Hz, 2H), 4.66 (d, *J* = 5.9 Hz, 2H), 2.35 (s, 3H), 1.55 (m, 1H).

^{13}C{^{1}H} NMR (101 MHz, $CDCl_3$, 296 K) δ 137.92, 137.44, 129.27 (2C), 127.14 (2C), 65.32, 21.17.

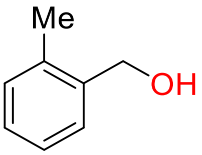

2-methyl-Benzenemethanol **(24)**[17]

$C_8H_{10}O$, white solid.

^{1}H NMR (400 MHz, $CDCl_3$, 296 K) δ 7.40-7.32 (m, 1H), 7.25 – 7.15 (m, 3H), 4.71 (d, *J* = 5.8 Hz, 2H), 2.37 (s, 3H), 1.51 (t, *J* = 5.9 Hz, 1H).

^{13}C{^{1}H} NMR (101 MHz, $CDCl_3$, 296 K) δ 138.8, 136.3, 130.5, 127.9, 127.7, 126.2, 63.8, 18.8.

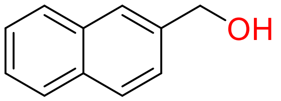

2-Naphthalene methanol **(25)**[17]

$C_{11}H_{10}O$, white solid.

^{1}H NMR (400 MHz, $CDCl_3$, 296 K) δ 7.87 – 7.80 (m, 4H), 7.53 – 7.45 (m, 3H), 4.87 (d, *J* = 3.8 Hz, 2H), 1.78 (s, 1H).

^{13}C{^{1}H} NMR (101 MHz, $CDCl_3$, 296 K) δ 138.4, 133.5, 133.1, 128.5, 128.0, 127.9, 126.3, 126.1, 125.6, 125.3, 65.7.

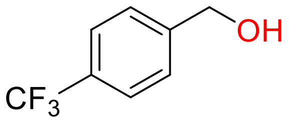

4-(trifluoromethyl)-Benzyl alcohol **(27)**[8]

$C_8H_7OF_3$, colorless oil.

^{1}H NMR (400 MHz, $CDCl_3$, 296 K) δ 7.62 (d, *J* = 8.1 Hz, *2*H), 7.48 (d, *J* = 7.9 Hz, 2H), 4.78 (d, *J* = 5.4 Hz, 2H), 1.82 (s, *1*H).

^{13}C{^{1}H} NMR (101 MHz, $CDCl_3$, 297 K) δ 144.8 (d, *J* = 1.4 Hz), 129.9 (q, *J* = 32.3 Hz), 126.9 (2C), 125.6 (q, *J* = 3.9 Hz, 2C), 122.9, 64.5.

^{19}F{^{1}H} NMR (376 MHz, $CDCl_3$, 296 K) δ −62.5.

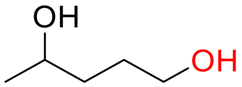

Pentan-1,4-diol **(30)**[18]

$C_5H_{12}O_2$, colorless oil

^{1}H NMR (400 MHz, $CDCl_3$, 296 K) δ 3.85 (ddd, J = 7.7, 6.1, 3.7 Hz, 1H), 3.74-3.58 (m, 2H), 2.45 (s, 2H), 1.75-1.57 (m, 3H), 1.57-1.44 (m, 1H), 1.21 (d, *J* = 6.2 Hz, 3H).

^{13}C{^{1}H} NMR (101 MHz, $CDCl_3$, 297 K) δ 67.8, 62.8, 36.1, 29.0, 23.5.

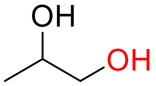

Propylene Glycol **(31)**

$C_3H_8O_2$, colorless oil.

^{1}H NMR (400 MHz, $CDCl_3$, 296 K) δ 3.83 (m, 1H), 3.55 (dd, *J* = 11.2, 3.0 Hz, 1H), 3.32 (dd, *J* = 11.2, 7.8 Hz, 1H), 2.87 (br s, 2H), 1.09 (d, *J* = 6.3 Hz, 3H).

^{13}C{^{1}H} NMR (101 MHz, $CDCl_3$, 297 K) δ 68.3, 68.0, 18.8.

5. Preparative Scale Reaction

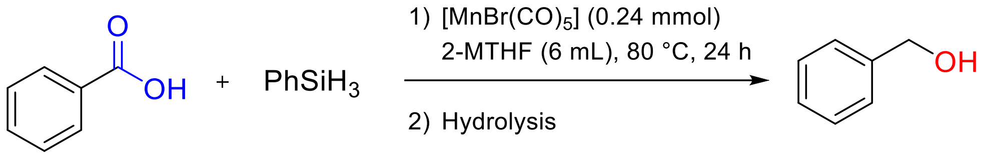

(12 mmol) (18 mmol)

Scheme S13: Reaction between phenylacetic acid and $PhSiH_3$ in the presence of manganese catalyst.

$[MnBr(CO)_5]$ (66.0 mg, 0.24 mmol) and phenylacetic acid (1.63 g, 12.0 mmol) were placed into a Schlenk tube equipped with a screw cap and containing a magnetic stirrer. The reaction vessel was evacuated and filled with argon three times. Phenylsilane (1.95 g, 18.0 mmol) and 2-MTHF (6 mL) were added under argon, and the mixture was heated at 80 °C. During this stage, the Schlenk tube was connected via a Schlenk line to a bubbler to prevent overpressure in the apparatus due to initial H_2 evolution. After 24 hours, the reaction vessel was cooled down to room temperature and an aqueous NaOH solution (10% w/w, 25 mL) was added dropwise to the reaction. Then, the resulting mixture stirred overnight. The crude product was extracted with 2-MTHF (4 × 25 mL). The combined organic layers were dried over $MgSO_4$, filtered, and the solvents removed under reduced pressure. The crude product was purified by a bulb to bulb distillation under vacuum (Kugelrohr distillation), affording 1.35 g of 2-phenylethanol (11.1 mmol, Yield = 93%).

6. Yield Time Profile

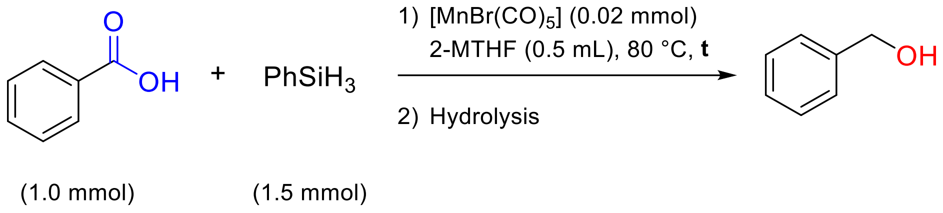

Scheme S14: Reaction between phenylacetic acid and $PhSiH_3$ in the presence of manganese catalyst.

$[MnBr(CO)_5]$ (5.5 mg, 0.02 mmol) and phenylacetic acid (136.2 mg, 1.0 mmol) were placed into a Schlenk tube equipped with a screw cap and containing a magnetic stirrer. The reaction vessel was evacuated and filled with argon three times. Phenylsilane (162.3 mg, 1.5 mmol) and 2-MTHF (0.5 mL) were added under argon flush, and the mixture was heated at 80 °C. The reaction was left stirring for the specific allotted time shown the Table S9. After cooling down the reaction vessel to room temperature, MeOH (6 mL) and an aqueous NaOH solution (10% w/w, 4 mL) were added dropwise. Then, the resulting reaction mixture was stirred overnight. Before extraction, ferrocene (37.2 mg, 0.2 mmol) was dissolved in DCM (4 mL) and added as an internal. The crude product was then extracted extracted from the aqueous phase with DCM (3 × 10 mL). The combined organic layers were dried over $MgSO_4$, filtered, and the solvents removed under reduced pressure. Yields were obtained by setting the integral value for the ferrocene singlet (4.16 ppm, 10H) as 1.00 and integrating the characteristic signal of the 2-Phenylethanol (2.88 ppm, 2H).

Table S9 – Results of the yield-time profile for the hydrosilylation of phenylacetic acid in conditions used for the gram-scale synthesis. The graph is seen below.

Reaction time	Yield (%)
0 min	00
5 min	40
10 min	49
15 min	55
30 min	59
1 h	66
2 h	75
4 h	86
6 h	91
8 h	93
24 h	96

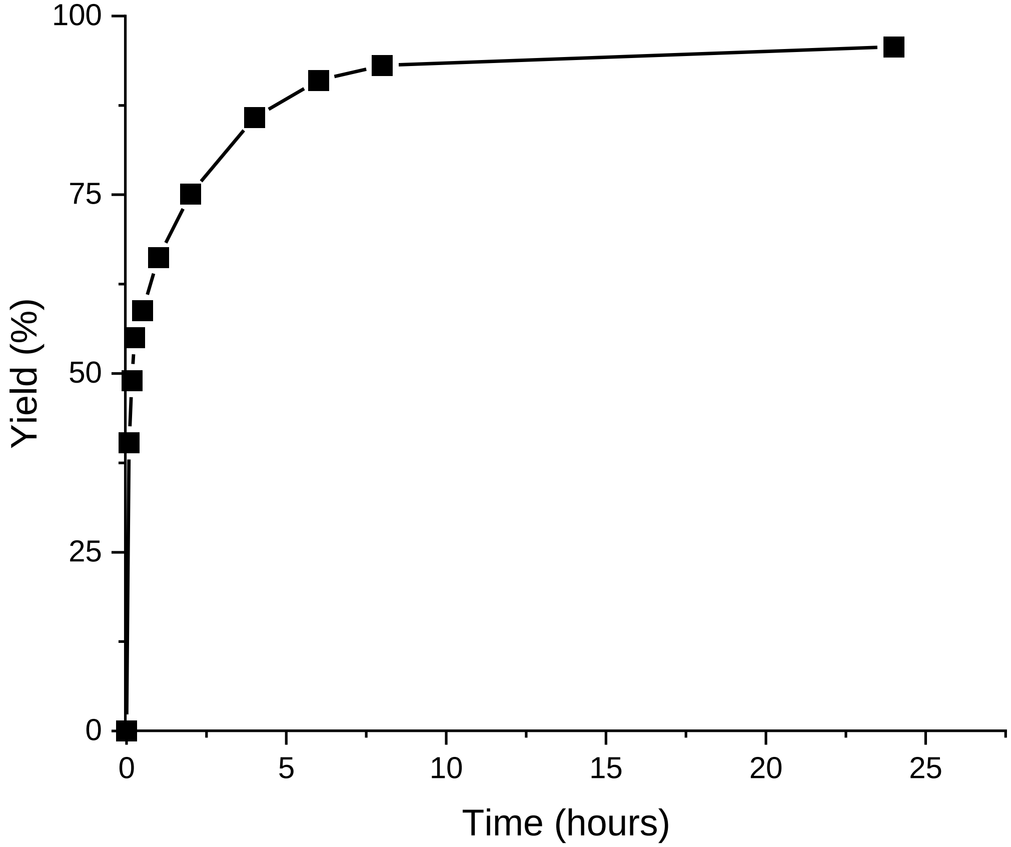

Graph S1 – Yield-time profile for the hydrosilylation reaction of phenylacetic acid with [$MnBr(CO)_5$] catalyst and 1.5 equivalents of phenylsilane.

7. Gas-phase Analysis Studies

7.1. General Procedure

An oven-dried Schlenk tube equipped with a screw cap and containing a magnetic stirrer is evacuated and filled with argon three times. $PhSiH_3$ (2.5 mmol), phenylacetic acid (1 mmol), the respective manganese catalyst (0.02 mmol), and dry and degassed 2-MTHF (0.5 mL) were added under argon. The Schlenk tube was then rapidly closed with a cap equipped with a septum and placed in a pre-heated oil bath at 80 °C for the required amount of time. The reaction mixture was allowed to cool down to room temperature, and the gas phase (100 µL) was injected into a GC instrument. The quantification of $H_{2(g)}$ and $CO_{(g)}$ has been performed using calibration curves corresponding to the respective integrated areas of the two gases.

7.2. Reactions Performed

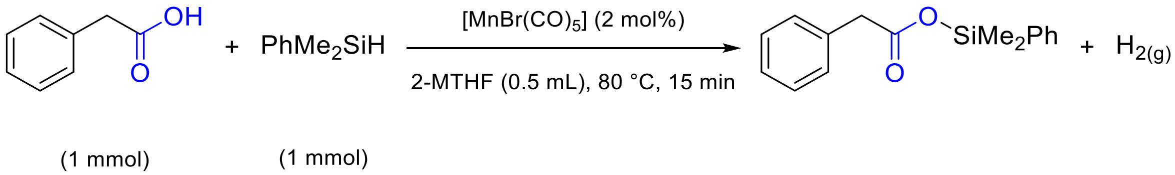

Scheme S15: Reaction between phenylacetic acid and $PhMe_2SiH$.

Gas identified	Retention time	Vol%
H_2	2.22	66.525
Ar	3.53	30.398
CO	4.61	3.077

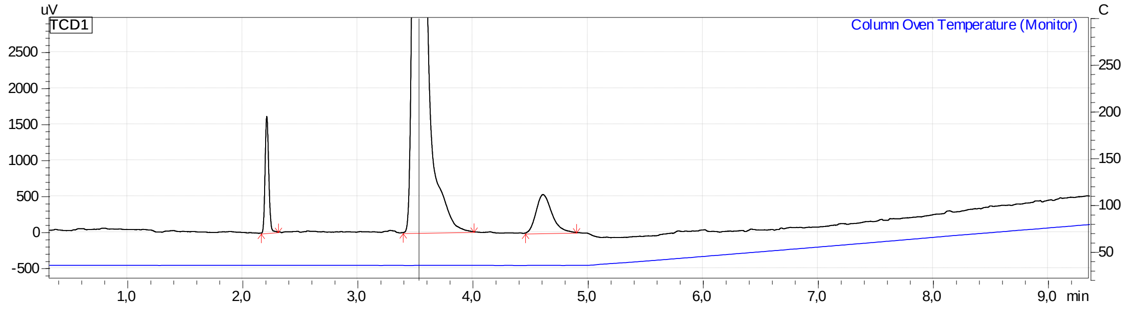

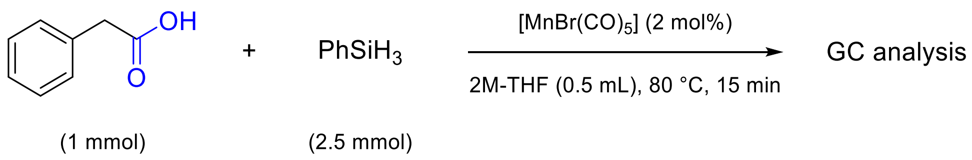

Scheme S16: Reaction between phenylacetic acid and $PhSiH_3$.

Gas identified	Retention time	Vol%
H_2	2.22	66.6
Ar	3.53	32.1
CO	4.62	1.3

Ratio H_2:CO = 51

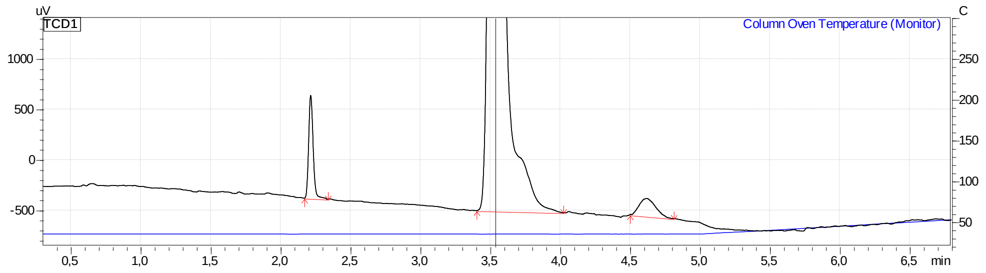

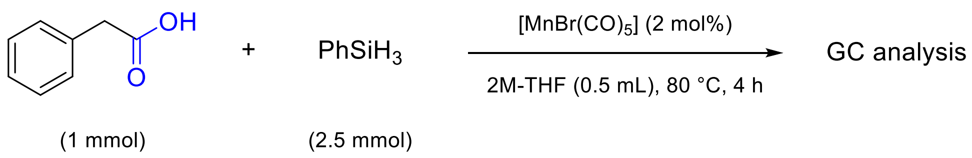

Scheme S17: Reaction between phenylacetic acid and $PhSiH_3$.

Gas identified	Retention time	Vol%
H_2	2.22	68.2
Ar	3.54	30.4
CO	4.62	1.4

Ratio H_2:CO = 49

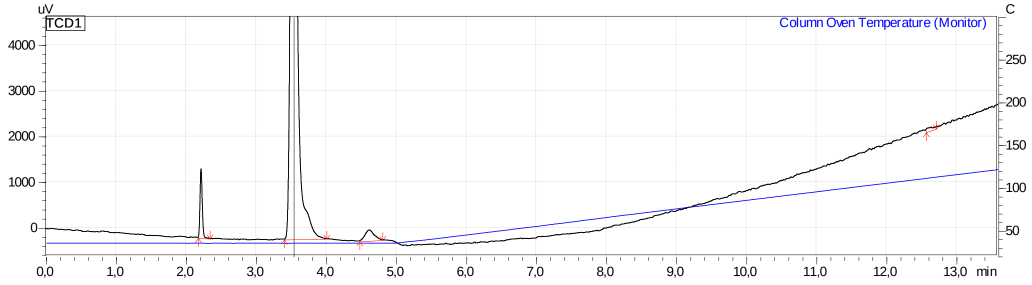

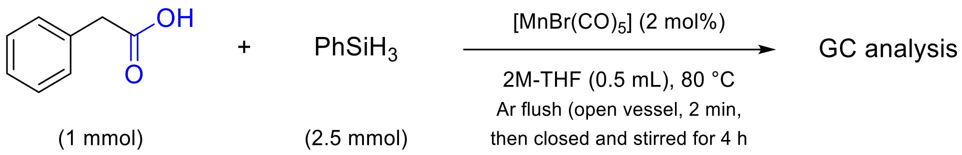

Scheme S18: Reaction between phenylacetic acid and $PhSiH_3$.

Gas identified	Retention time	Vol%
Ar	3.53	29.9
N_2	3.73	70.1

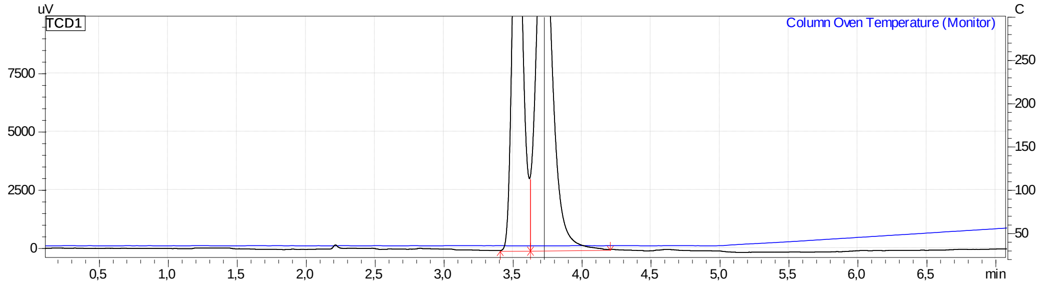

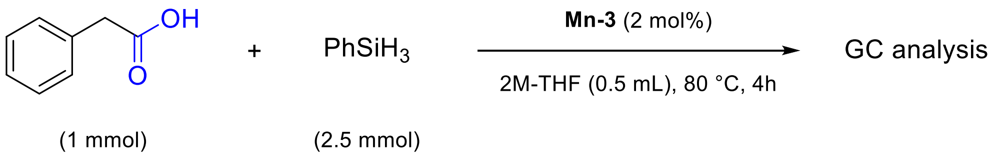

Scheme S19: Reaction between phenylacetic acid and $PhSiH_3$.

Gas identified	Retention time	Vol%
H_2	2.01	54.7
Ar	3.32	36.3

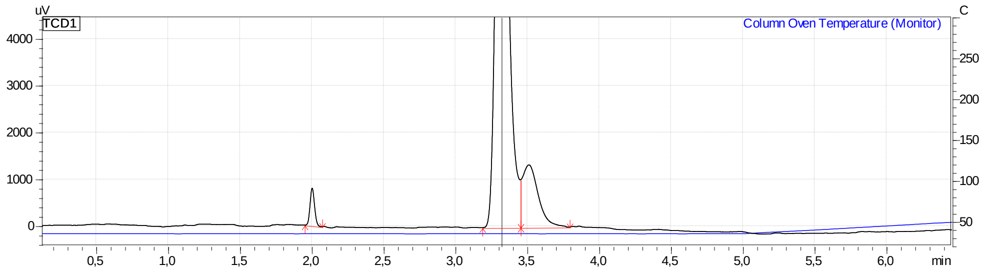

8. Kinetic Experimentation

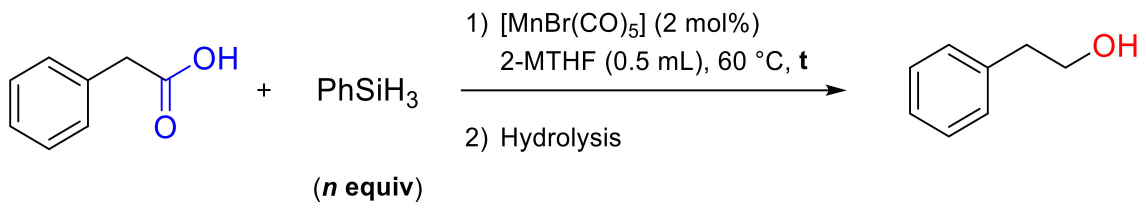

Scheme S20: Reaction between phenylacetic acid and $PhSiH_3$.

An *Initial Rates* method was used to monitor the kinetic profile of the start of the reaction (most reactions were stopped at yields < 20%). Four single-point reactions were performed at different times within the first 15 minutes, using Ferrocene as an internal standard (added after hydrolysis). The method used does not allow for the determination of fractional orders. As a result, the precision of the measurement that gives a 1st order correlation has to be considered between 0.5-1.5 orders.

Three different concentrations of either reagents or catalysts were used to determine the order of the reaction. Each time point for the middle concentration of each set was repeated for reliability.

Example data, reaction procedure, and rate profile used to determine the order of the reaction with respect to [$MnBr(CO)_5$] is given below:

Varying amounts of [$MnBr(CO)_5$] ((**1**) 5.5 mg, 0.02 mmol) ((**2**) 2.8 mg, 0.01 mmol) ((**3**) 8.3 mg, 0.03 mmol), and Phenylacetic acid (136.2 mg, 1.00 mmol) were placed into a Schlenk tube equipped with a screw cap and containing a magnetic stirrer. The reaction vessel was evacuated and filled with argon three times. Phenylsilane (270.5 mg, 2.5 mmol) and 2-MTHF (0.5 mL) were added under argon, and the mixture was heated at 60 °C. The reaction was left stirring for the specific allotted time shown the Table S10. After cooling down the reaction vessel to room temperature, MeOH (6 mL) and an aqueous NaOH solution (10% w/w, 4 mL) were added dropwise. Then, the reaction mixture was stirred overnight. Before extraction, ferrocene (18.6 mg, 0.1 mmol) was dissolved in DCM (4 mL) and added as an internal. The crude product was then extracted from the aqueous phase with DCM (3 × 10 mL). The combined organic layers were dried over $MgSO_4$, filtered, and the solvents removed under reduced pressure. Yields were obtained by setting the integral value for the ferrocene singlet (4.16 ppm, 10H) as 1.00 and integrating the characteristic signal of the 2-Phenylethanol (2.88 ppm, 2H).[2]

[2] All spectra were processed performing a peak deconvolution analysis when necessary.

Table S10 – Data obtained for the Kinetic Plots to determine the order of the reaction with respect to [$MnBr(CO)_5$] is given below.

	Catalyst Concentration, mol dm^{-3}	Reaction Time, s	Concentration of Product, mol dm^{-3}	Initial Observed Rate, x10^{-4} M s^{-1}
1	0.04	000	0.0000	3.9096
1	0.04	150	0.0831	
1	0.04	150	0.0587	
1	0.04	300	0.1175	
1	0.04	300	0.1089	
1	0.04	600	0.2223	
1	0.04	600	0.2603	
1	0.04	900	0.3305	
1	0.04	900	0.3620	
2	0.02	000	0.0000	1.8937
2	0.02	150	0.0231	
2	0.02	300	0.0434	
2	0.02	600	0.0977	
2	0.02	900	0.1864	
3	0.06	000	0.0000	5.2324
3	0.06	150	0.0990	
3	0.06	300	0.1558	
3	0.06	600	0.3089	
3	0.06	900	0.4712	

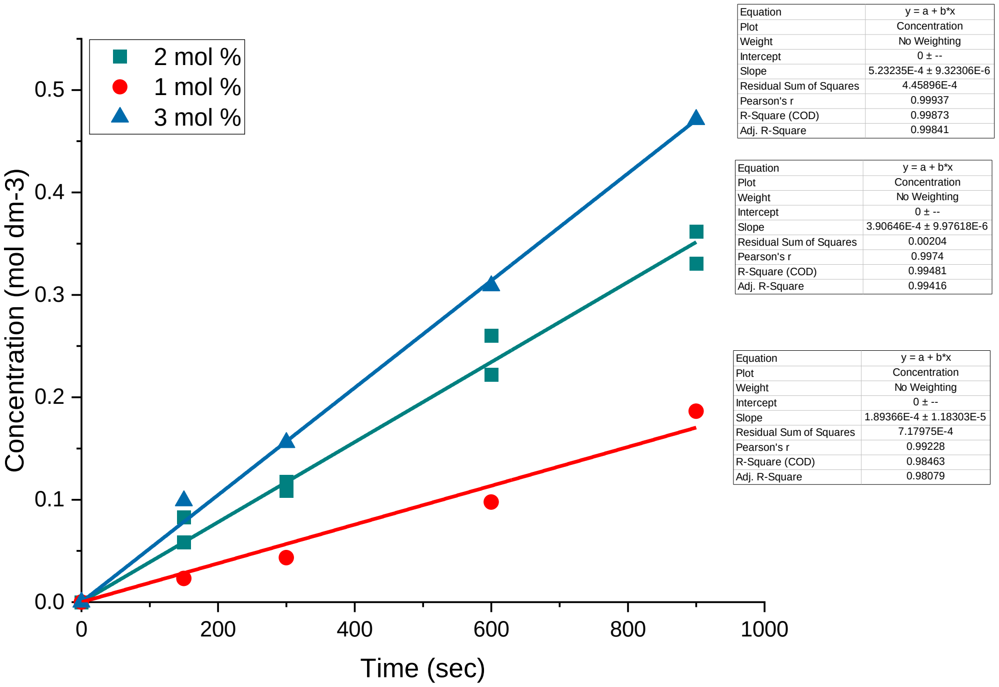

Graph S2 – Concentration of 2-Phenylethanol against time for each of the following [MnBr(CO)$_5$] concentrations: blue, 0.06 mol dm^{-3} – reaction 3; turquoise, 0.04 mol dm^{-3} – reaction 1; red, 0.02 mol dm^{-3} – reaction 2.

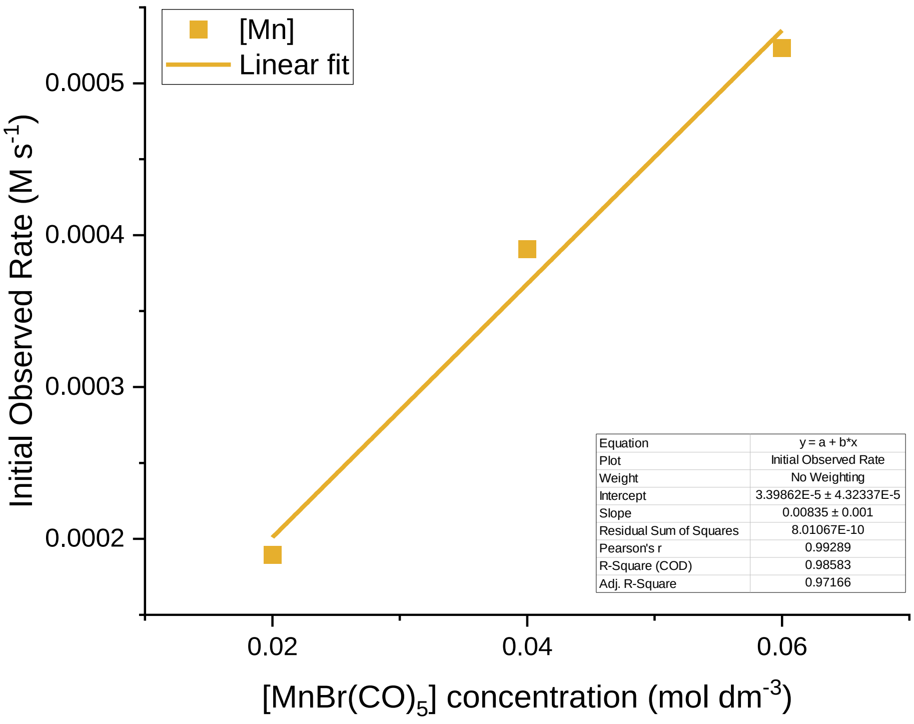

Graph S3 – Initial rates as a function of [MnBr(CO)$_5$] concentration.

Only graphical data for the rate profile is given for varying Phenylsilane concentration and Phenyl Acetic acid concentrations. The equivalent procedure was followed.

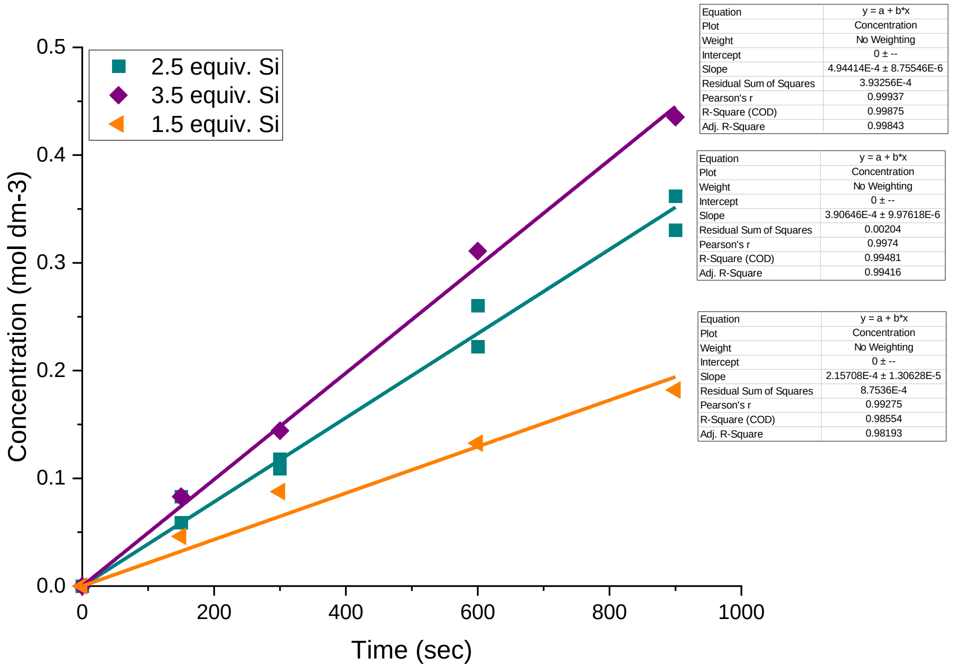

Graph S4 – Concentration of 2-Phenylethanol against time for each of the following Phenylsilane concentrations: purple, 2.96 mol dm^{-3}; turquoise, 5.20 mol dm^{-3}; orange, 7.16 mol dm^{-3}.

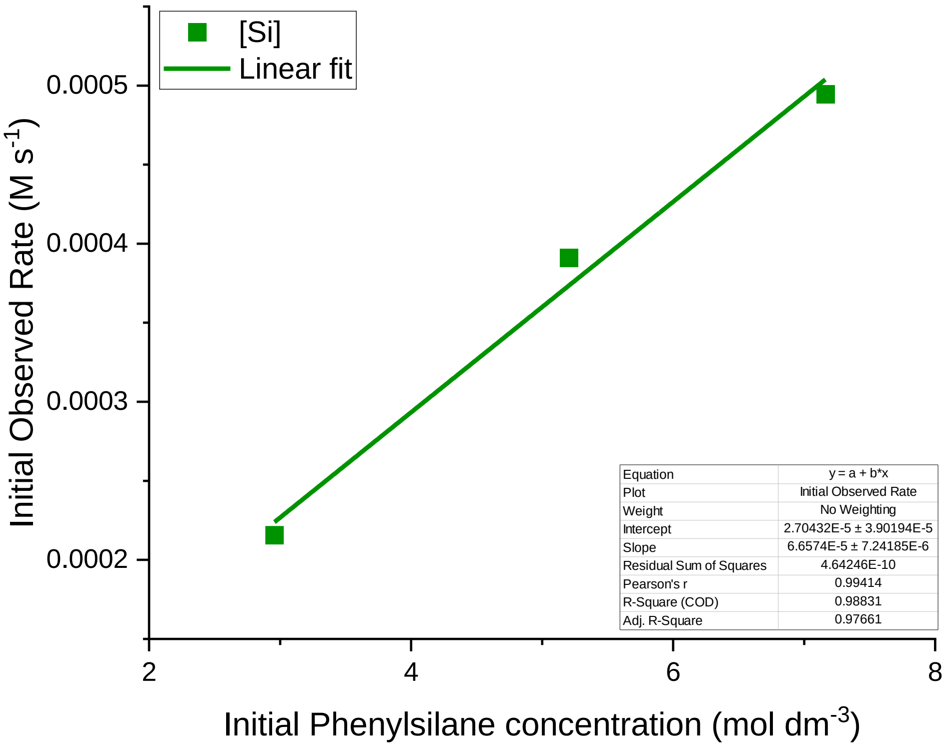

Graph S5 – Initial rates as a function of Phenylsilane concentration.

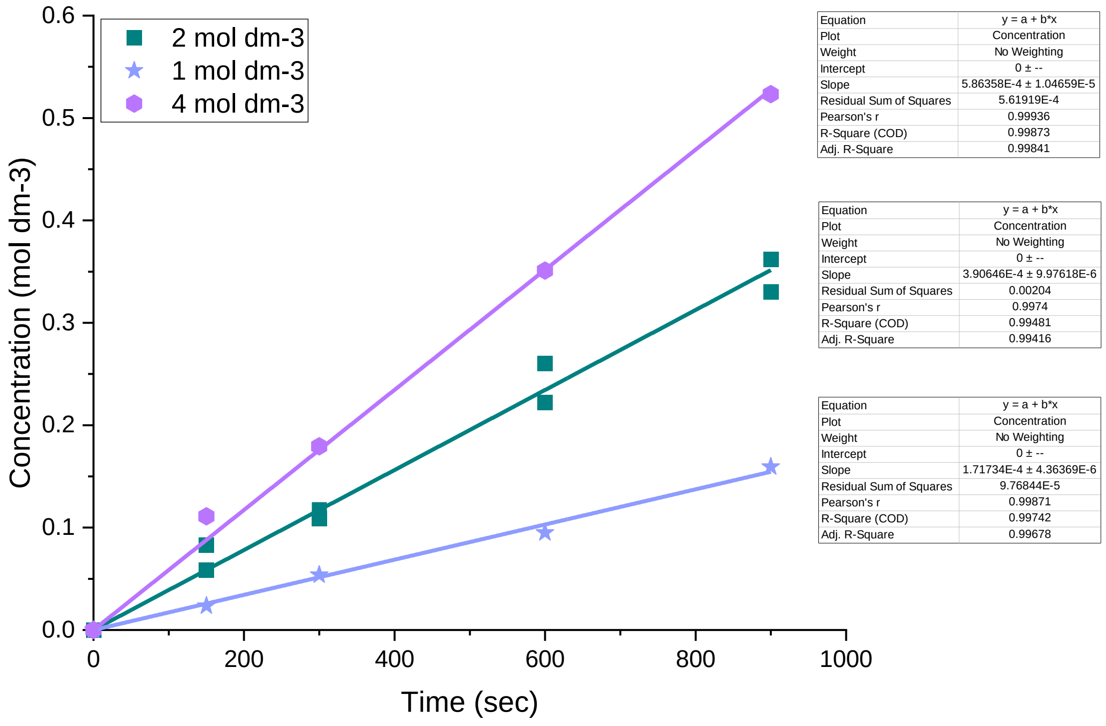

Graph S6 – Concentration of 2-Phenylethanol against time for each of the following Phenylacetic acid concentration: light purple, 4 mol dm^{-3}; turquoise, 2 mol dm^{-3}; pale blue, 1 mol dm^{-3}.

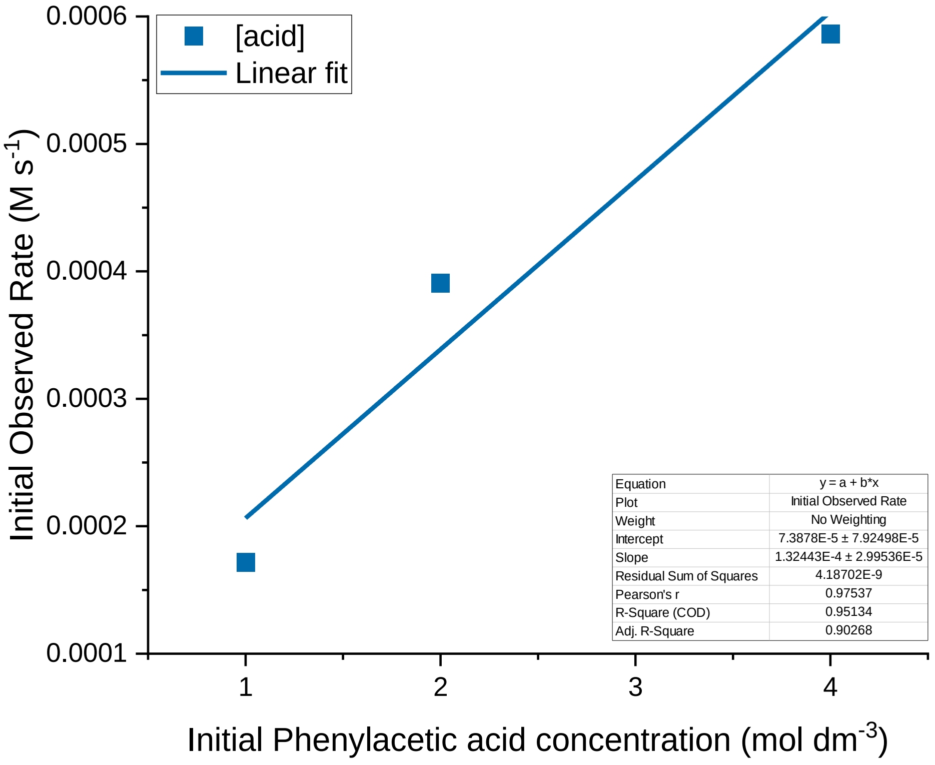

Graph S7 – Initial rates as a function of Phenylacetic acid concentration.

9. Mechanistic Studies

9.1. Synthesis of the Silyl Ester

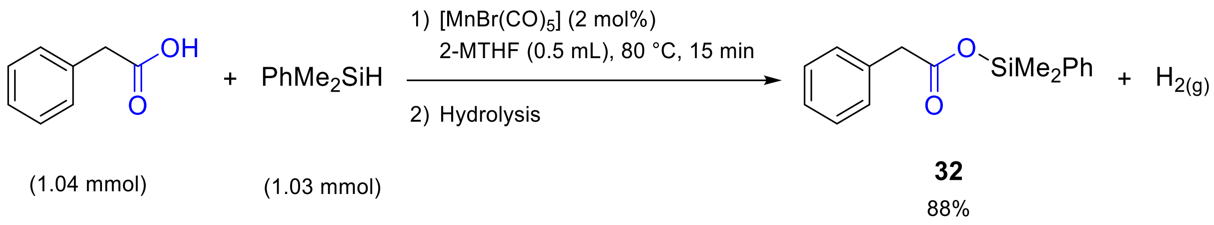

Scheme S21: Reaction between phenylacetic acid and $PhMe_2SiH$.

- **NMR yield**

Dimethylphenylsilane (140.8 mg, 1.03 mmol) has been weighed inside the glovebox into an oven-dried Schlenk tube equipped with a screw cap and containing a magnetic stirrer. Phenylacetic acid (141.2 mg, 1.04 mmol), $[MnBr(CO)_5]$ (5.6 mg, 0.02 mmol), and dry and degassed 2-MTHF (0.5 mL) were added to the Schlenk tube under argon. The Schlenk tube was closed and placed at 80 °C in a pre-heated oil bath for 15 minutes. Then, the reaction vessel was cooled down, the mixture concentrated under reduced pressure, and mesitylene (61.9 mg, 0.52 mmol) was added under argon. After stirring for 5 minutes, the reaction mixture (ca. 0.1 mL) was transferred into an oven-dried NMR tube, and THF-d_8 was added to reach 0.5 mL of solution. Yields were obtained by setting the integral value for mesitylene (6.75 ppm) as 1.00 and integrating the characteristic signal of the product (0.51 ppm).

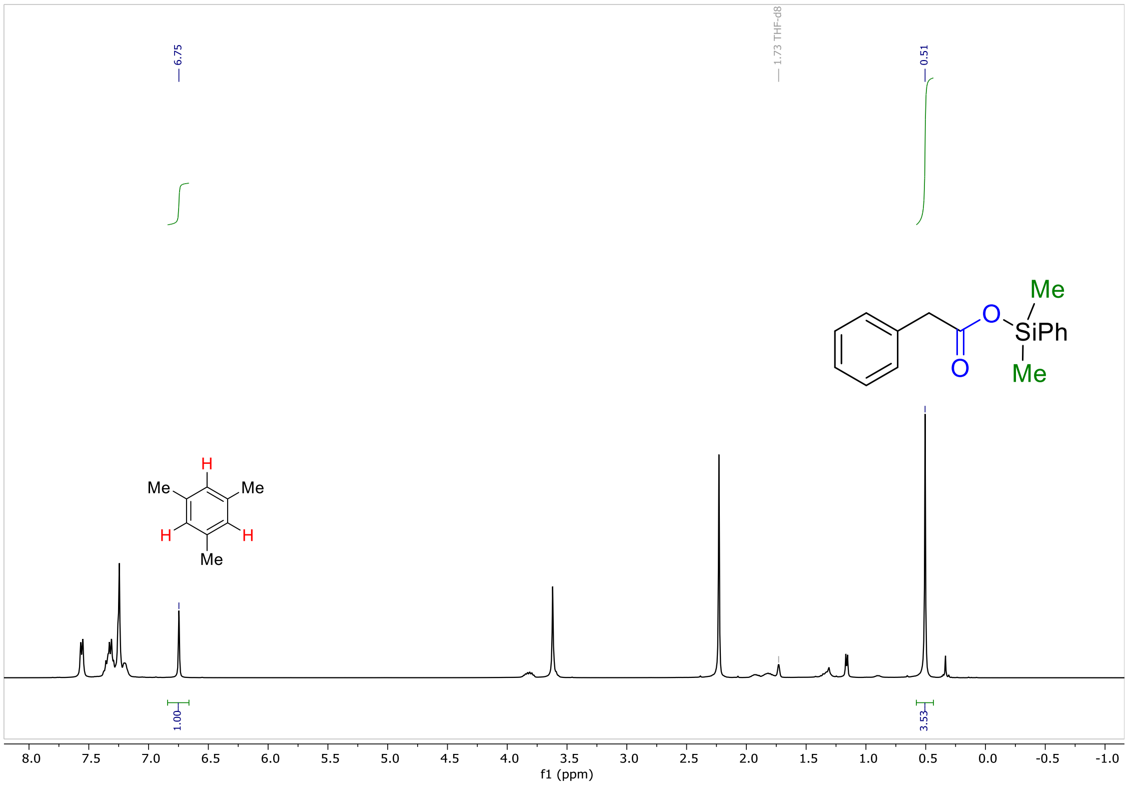

Figure S1 – ^{1}H NMR (400 MHz, THF-d_8, 296 K) spectrum of silyl ester formation. NMR yield.

9.2. Isolation of the Silyl Ester

Dimethylphenylsilane (137.6 mg, 1.01 mmol) was weighed inside the glovebox into an oven-dried Schlenk tube equipped with a screw cap and containing a magnetic stirrer. Phenylacetic acid (137.4 mg, 1.01 mmol), [MnBr(CO)$_5$] (5.8 mg, 0.02 mmol), and dry and degassed 2-MTHF (0.5 mL) were added under argon flow. The Schlenk tube was closed and placed at 80 °C in a pre-heated oil bath for 15 minutes. After cooling down the reaction mixture to room temperature, the solvent was evaporated under reduced pressure. The concentrated reaction mixture was then transferred into an oven-dried Kugelrohr distillation flask to perform a Kugelrohr distillation. We note that only oven-dried distillation flasks were used. This procedure, similar to others described in the literature for the isolation of similar compounds,[19] allowed the recovery of 221.3 mg of product (Yield = 81 %).

HR-MS: m/z calculated for $[C_{16}H_{18}O_2SiNa]^+$ = 293.09683, found = 293.09679

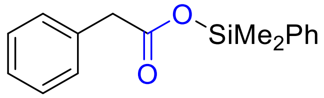

^{1}H NMR (400 MHz, CDCl$_3$, 296 K) δ 7.56 – 7.42 (m, 2H), 7.41 – 7.17 (m, 8H), 3.58 (s, 2H), 0.47 (s, 6H). **^{13}C{^{1}H} NMR** (101 MHz, THF-d_8, 296 K) δ 171.9, 136.9, 135.9, 134.5, 130.9 (d), 130.3, 129.2 (d), 128.7 (d), 127.6 (d), 43.4 (d), -1.3 (d).

The product is susceptible to hydrolysis. To perform an optimal distillation, the use of a well-dried collection flask is recommended. Also, the use of more than one collection flask is suggested.

9.3. Reaction of the Silyl Ester with $PhSiH_3$

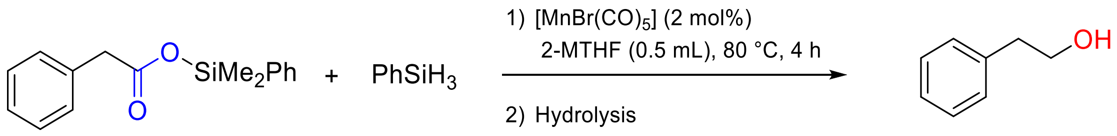

Scheme S22: Reaction between silyl ester **32** and $PhSiH_3$.

Silyl ester **32** (272.8 mg, 1.0 mmol) was weighed inside the glovebox into an oven-dried Schlenk tube equipped with a screw cap and containing a magnetic stirrer. After connecting the Schlenk tube to a Schlenk line, $PhSiH_3$ (266.3 mg, 2.5 mmol), $[MnBr(CO)_5]$ (5.8 mg, 0.02 mmol), and dry and degassed 2-MTHF (0.5 mL) are successively added under argon. The Schlenk tube was then closed and placed at 80 °C in a pre-heated oil bath for 4 hours. After cooling down the reaction vessel to room temperature, MeOH (6 mL) and an aqueous NaOH solution (10% w/w, 4 mL) were added dropwise. Then, the resulting reaction mixture was stirred overnight. Before extraction, ferrocene (18.6 mg, 0.1 mmol) was dissolved in DCM (4 mL) and added as an internal. The crude product was then extracted from the aqueous phase with DCM (3 × 10 mL). The combined organic layers were dried over $MgSO_4$, filtered, and the solvents removed under reduced pressure. The NMR yield was obtained by setting the integral value for the ferrocene singlet (4.16 ppm, 10H) as 1.00 and integrating the characteristic signal of the 2-Phenylethanol (2.88 ppm, 2H).

9.4. Hydrogen Release Reaction

$[MnBr(CO)_5]$ (5.5 mg, 0.02 mmol) and phenyl acetic acid (136.1 mg, 1.0 mmol) were placed into a Schlenk tube equipped with a screw cap and containing a magnetic stirrer. The reaction vessel was evacuated and filled with argon three times. Phenylsilane (270.5 mg, 2.5 mmol) and 2-MTHF (0.5 mL) were added under argon. The Schlenk tube was closed and placed in an oil bath set to 80 °C until the bubbling ceased. While the Schlenk was still in the oil bath, the stopcock connected to the Schlenk line was opened to release the overpressure. Then, the cap of the Schlenk tube was removed, and argon was flushed for 2 minutes. The cap was placed back on the Schlenk tube, the stopcock closed again, and the reaction left to run for 4 hours. After cooling down the reaction vessel to room temperature, MeOH (6 mL) and aqueous NaOH (10% w/w, 4 mL) were added dropwise. Then, the reaction mixture was left stirring overnight. Before extraction, ferrocene (21.2 mg, 0.1 mmol) was dissolved in DCM (4 mL) and added as an internal. The crude product was then extracted from the aqueous phase with DCM (3 × 10 mL). The combined organic layers were dried over $MgSO_4$, filtered, and the solvents removed under reduced pressure. The yield was obtained by setting the integral value for the ferrocene singlet (4.16 ppm, 10H) as 1.00 and integrating the characteristic signal of the 2-Phenylethanol (2.88 ppm, 2H). Yield = 87%.

9.5. $^{31}P\{^1H\}$ NMR Analysis of Crude Reaction Mixtures

- **General Sample Preparation**

The indicated complex (0.01 mmol) was placed into a Schlenk tube equipped with a screw cap and containing a magnetic stirrer. The reaction vessel was evacuated and filled with argon three times. Phenylsilane (135.3 mg, 1.25 mmol), cyclohexane propanoic acid (78.1 mg, 0.5 mmol) and THF (0.2 mL) were added under argon. The reaction mixture was heated at 80 °C for 2 hours. Subsequently, the reaction mixture was allowed to cool down to room temperature, and the crude (0.25 mL) was transferred into an oven-dried NMR tube kept under argon. The respective dry NMR solvent was added to the NMR tube to a volume of approximately 0.5 mL. A $^{31}P\{^1H\}$NMR spectrum was then recorded at 296 K.

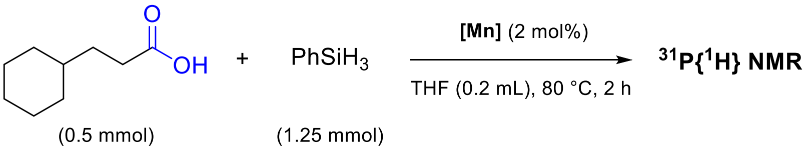

$^{31}P\{^{1}H\}$ Crude NMR (ppm)		
-16.14	133.66	136.15
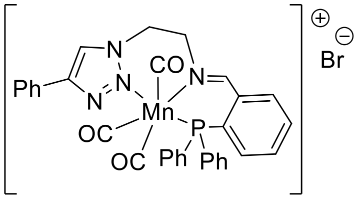 **Mn-2** 43.97[1]	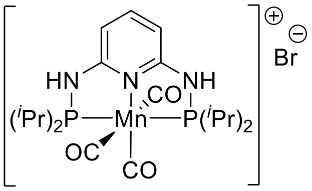 **Mn-3** 133.86[2]	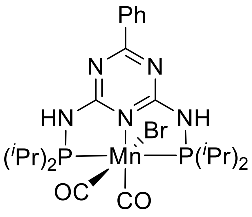 **Mn-4** 135.5[3]

Figure 2: Ligand environment investigation, by $^{31}P\{^{1}H\}$ NMR, on the manganese complexes with pincer ligands at the end of the reaction (before hydrolysis).

- **Reactions and Spectra**

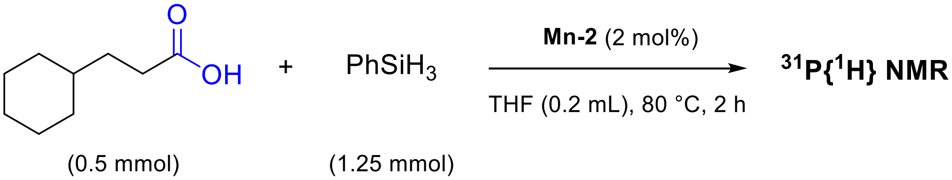

Scheme S23: Reaction protocol used to determine the phosphorus environment on the ligand of the complex **Mn-2** during the reaction.

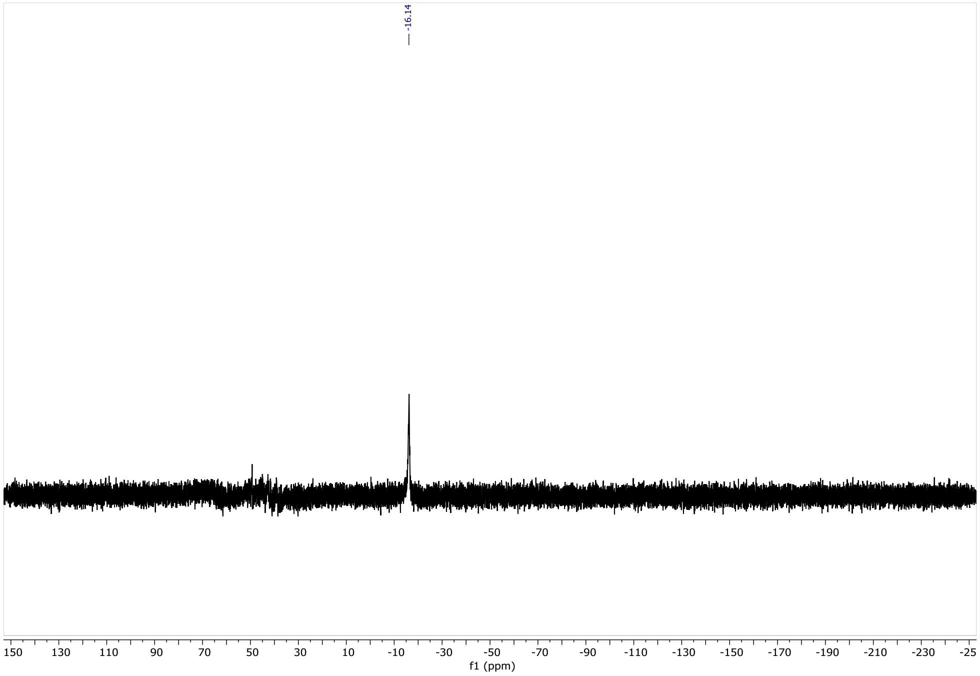

Figure S3 – **$^{31}P\{^1H\}$ NMR (162 MHz, THF-d_8, 296 K)** spectrum of **Mn-2** after the reaction reported in **Scheme S23**(−16.14 ppm).

For the purposes of the analysis, NMR spectra for the free ligand, the complex, and crude reaction mixture were all performed in THF-d_8.

^{31}P NMR for complex **Mn-2** in THF-d_8**:** 43.97 ppm (44.0 ppm in CD_2Cl_2)[1]

^{31}P NMR for the free ligand of **Mn-2** in THF-d_8: −13.13 ppm (−12.2 ppm in $CDCl_3$)[1]

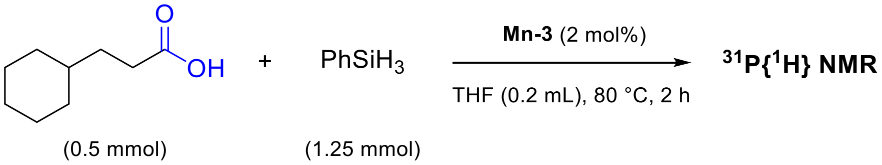

Scheme S24 Reaction protocol used to determine the phosphorus environment within the complex Mn-3 during the hydrosilylation step of the reaction.

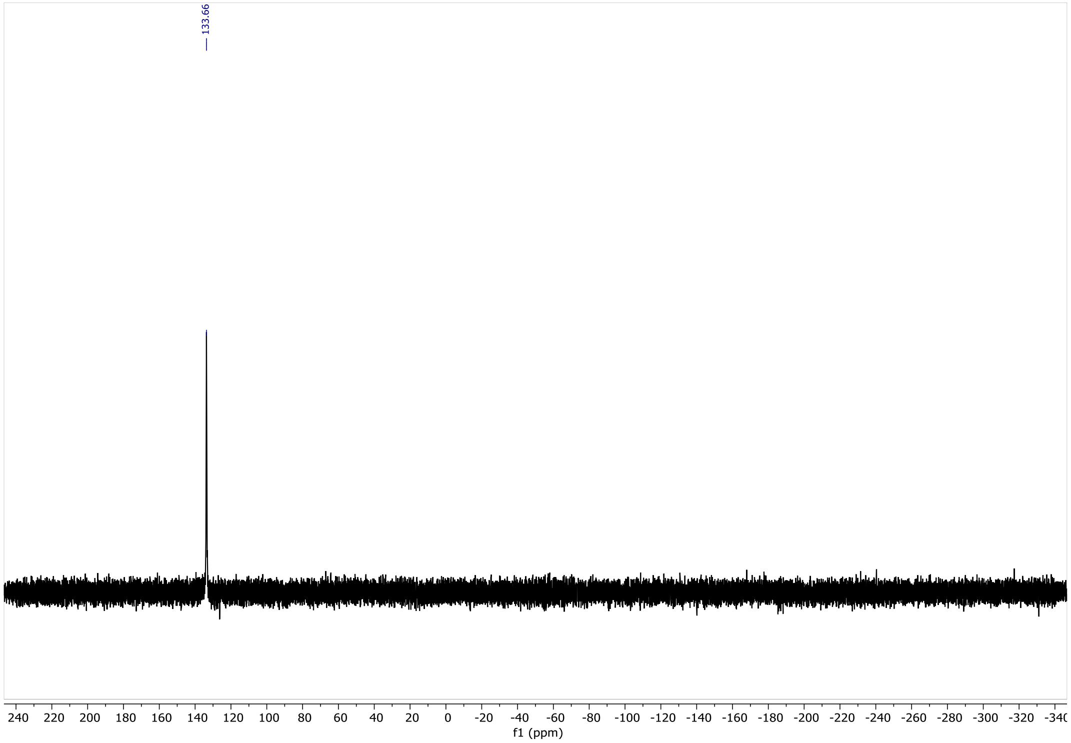

Figure S4 – **$^{31}P\{^{1}H\}$ NMR (162 MHz, DMSO-*d*$_6$, 296 K)** spectrum of Mn-3 after the reaction reported in Scheme S24. (133.66 ppm).

DMSO-*d*$_6$ has been used as NMR solvent to record the spectrum of the crude reaction mixture (as reported in the literature for the complex). Literature reported Phosphorus NMR of complex **Mn-3**: 133.86 ppm[2]

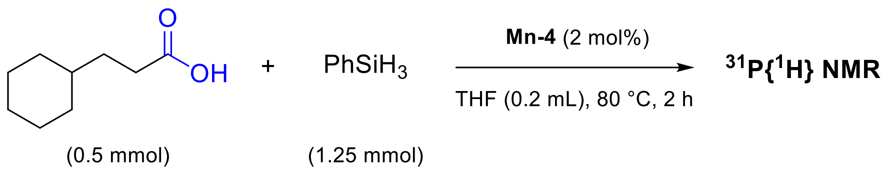

Scheme S25 – Reaction protocol used to determine the phosphorus environment within the complex Mn-4 during the hydrosilylation step of the reaction.

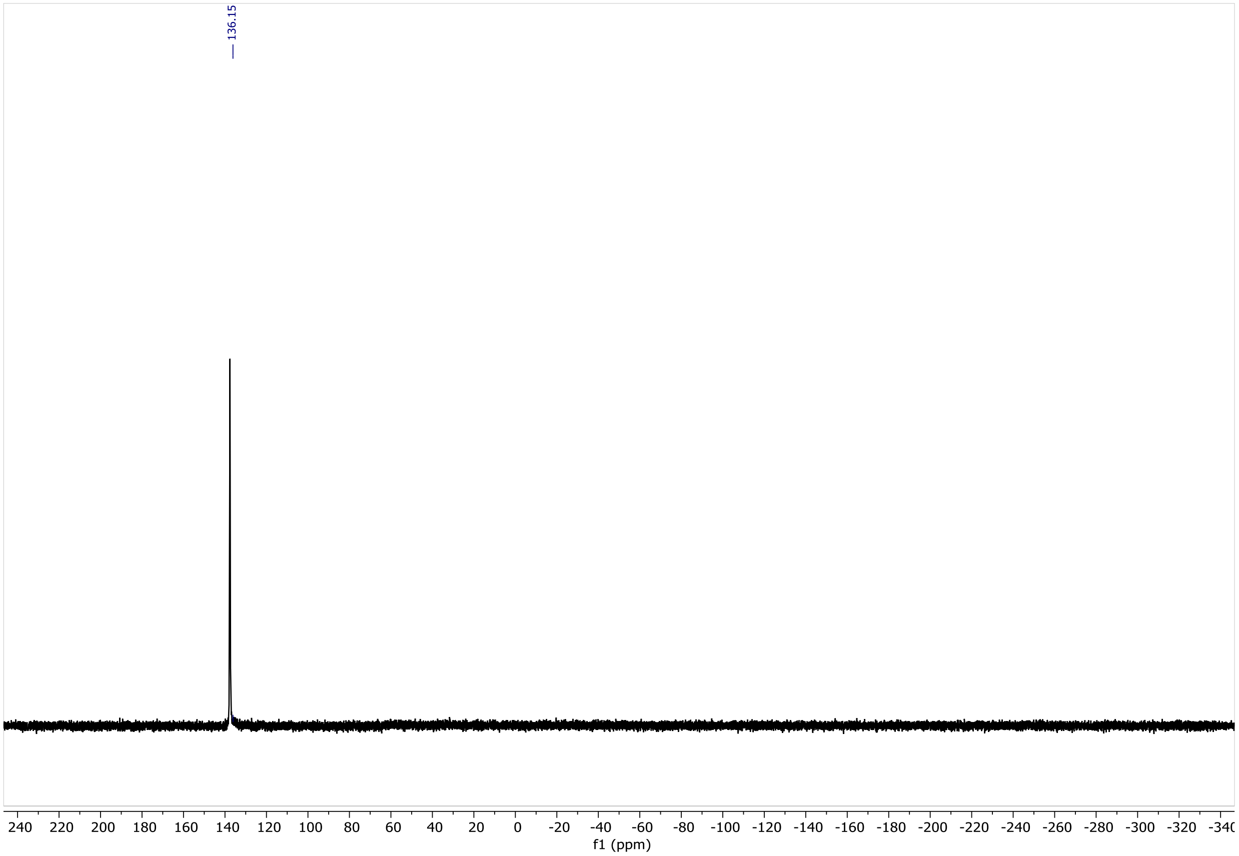

Figure S5 – **$^{31}P\{^1H\}$ NMR (162 MHz, $CDCl_3$, 296 K)** spectrum of **Mn-4** after the reaction reported in Scheme S25. (136.15 ppm).

$CDCl_3$ has been used as NMR solvent to record the spectrum of the crude reaction mixture (as reported in the literature for the complex).

Literature reported Phosphorus NMR of complex **Mn-4**: 135.5, 134.2 ppm[3]

10. Fate of the silane byproducts

10.1. Comment on the nature of the silane byproducts

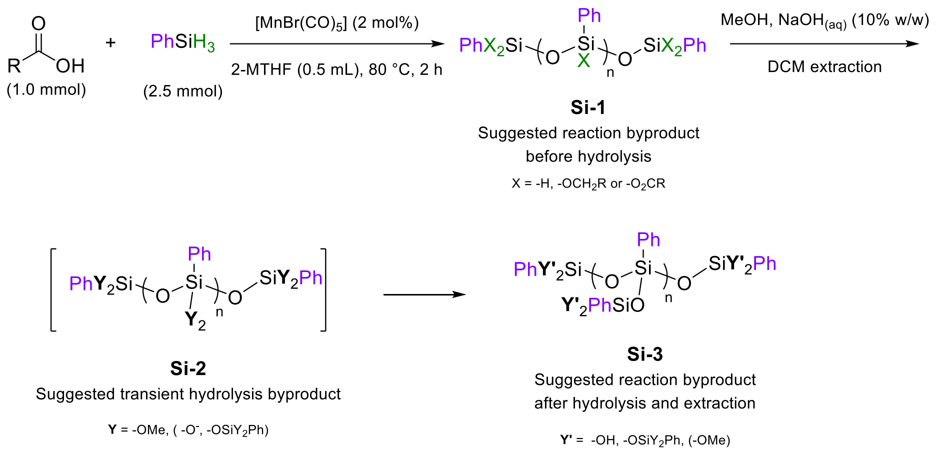

Scheme S26: Suggested reactions and products involving $PhSiH_3$ along with the reaction progress.

The complete cleavage of the C=O double bond of the carboxylic acid, together with the formation of gels when the reaction is performed with polymeric silanes (e.g., PMHS), indicates the formation of an oxygen bridging structure between silane units. Structures of the type **Si-1** [(Scheme S26, where -X (= -H, -O_2CR, -OCH_2R)] are suggested when using $PhSiH_3$ in the reaction. Crude ^{1}H-NMR and ^{19}F{^{1}H}-NMR performed at the end of the reaction before hydrolysis [respectively using cyclohexanecarboxylic acid (Scheme S27) and 4-Trifluoromethyl benzoic acid (Scheme S28) as substrates], confirm the identity of the suggested products of the type **Si-1.** A broad area between 5.25 and 4.75 ppm in the ^{1}H spectrum (Figure S6), with singlets ranging from 5.47 to 4.45 ppm, can be attributed to the residual hydrogens on **Si-1**. This is coherent with the expected shift to lower field with respect to the singlet of unreacted Phenylsilane (4.19 ppm) and with previous reports in the literature[20]. Also, a very broad peak between -64.85 and -65.1 ppm is present in the ^{19}F{^{1}H} spectrum of the silylated 4-Trifluoromethyl benzyl alcohol (Figure S7). This signal shape is in agreement with what was reported previously in the literature, where the use of a Zn^{2+} catalyzed $PhSiH_3$ reduction of amides also produced a very similar outcome on a fluorinated substrate in the ^{19}F{^{1}H}-NMR.[20]

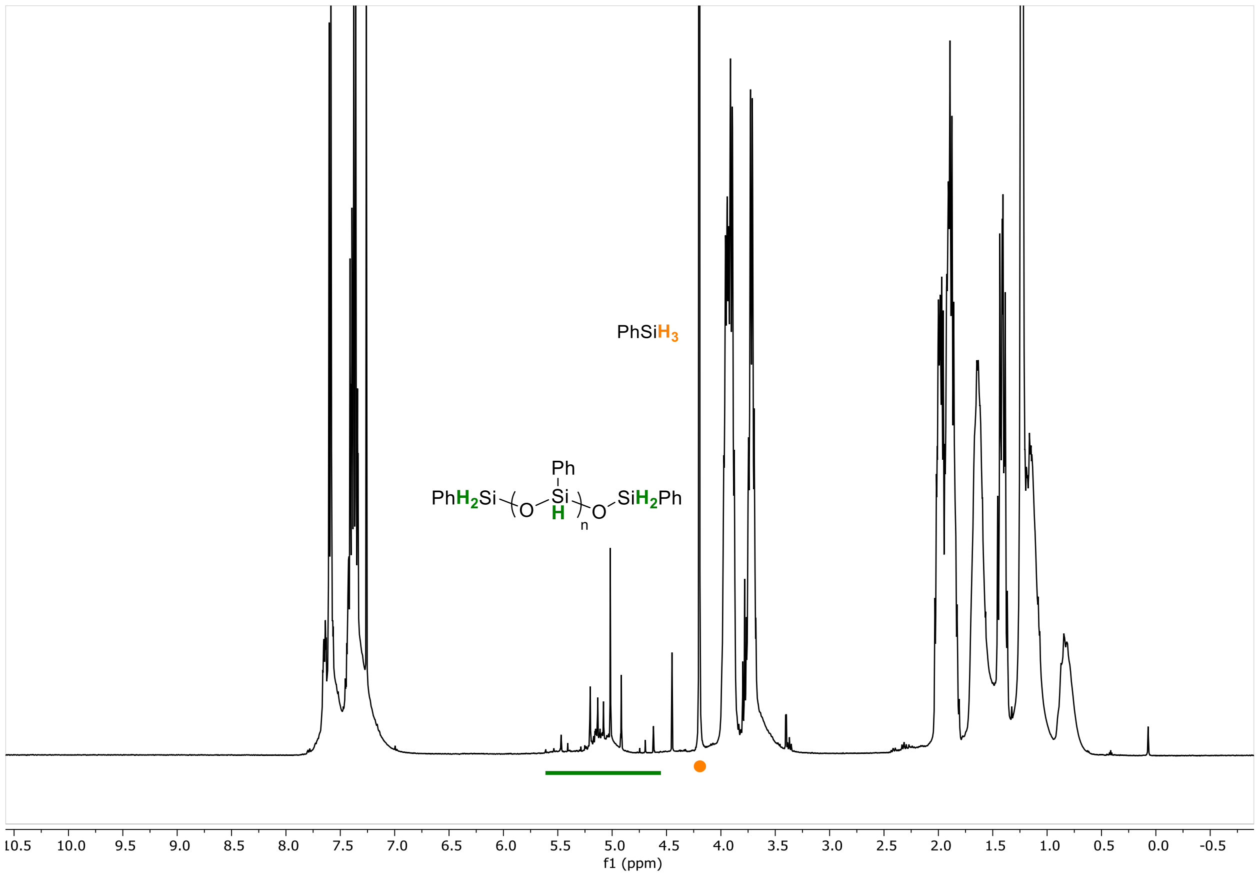

Figure S6 – **^{1}H NMR (376 MHz, CDCl₃, 296 K)** Spectrum of the crude silylated 3-Cychlohexylpropanol before hydrolysis.

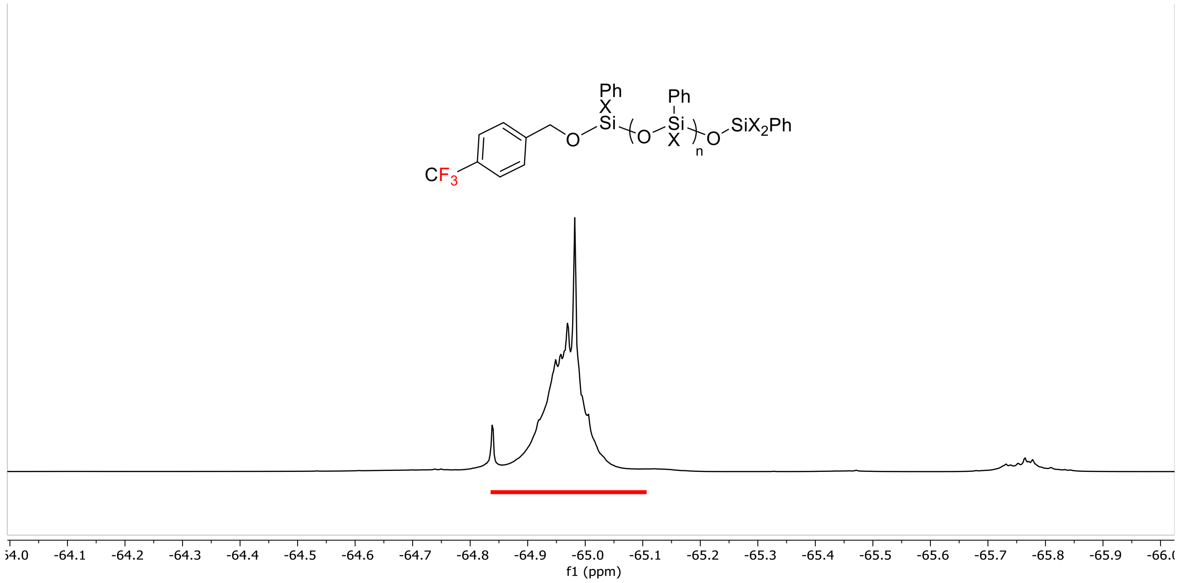

Figure S7 – **$^{19}F\{^1H\}$ NMR (376 MHz, THF-*d*₈, 296 K)** spectrum of the crude silylated 4-Trifluoromethylbenzyl alcohol before hydrolysis.

Upon hydrolysis, it is possible to observe a strong bubbling due to H_2 evolution of the residual Si-H bonds on the silane, used in excess. Complete substitution of -X (= -H, $-O_2CR$, $-OCH_2R$) with a methoxide or a hydroxide would lead to an alkoxysilane of the type **Si-2** (Scheme S26). Alkoxysilanes can form new bridging oxygen bonds in basic water solutions (reactivity at the base of the sol-gel synthesis), generating over time species of the type **Si-3** (Scheme S26). Due to the strongly basic conditions employed in the hydrolysis and the higher acidity of aryl-silanols compared to alcohols and water, most of the structures, **Si-3**, will be deprotonated in the water phase. As a result, only aggregates of a certain size will dissolve in the organic phase, leading to the extraction of a small fraction of the silane-based byproducts with the desired alcohol in DCM. This is confirmed by the presence of aromatic signals in the ^{1}H-NMR of aliphatic alcohols after hydrolysis and extraction (also in alcohols that afforded a nearly quantitative NMR yield, example reported in Figure S8).

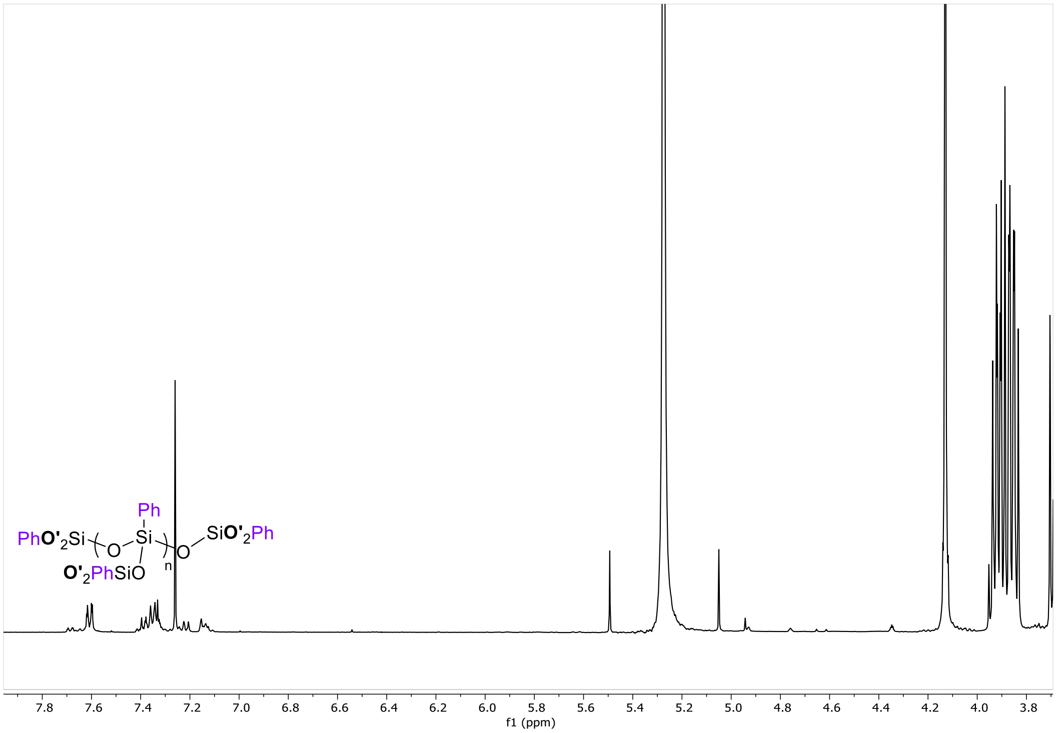

Figure S8: ^{1}H NMR (400 MHz, $CDCl_3$, 296 K) spectrum of the crude reaction in the aromatic region of a mixture after hydrolysis to recover the 96% NMR yield of 3-Cyclohexyl propanol.

It was also possible to isolate **Si-3** as a distillation residue in the form of a white solid, on which ^1H (Figure S9), $^{13}C\{^1H\}$-NMR (Figure S10) and Anionic APCI-MS have been performed.

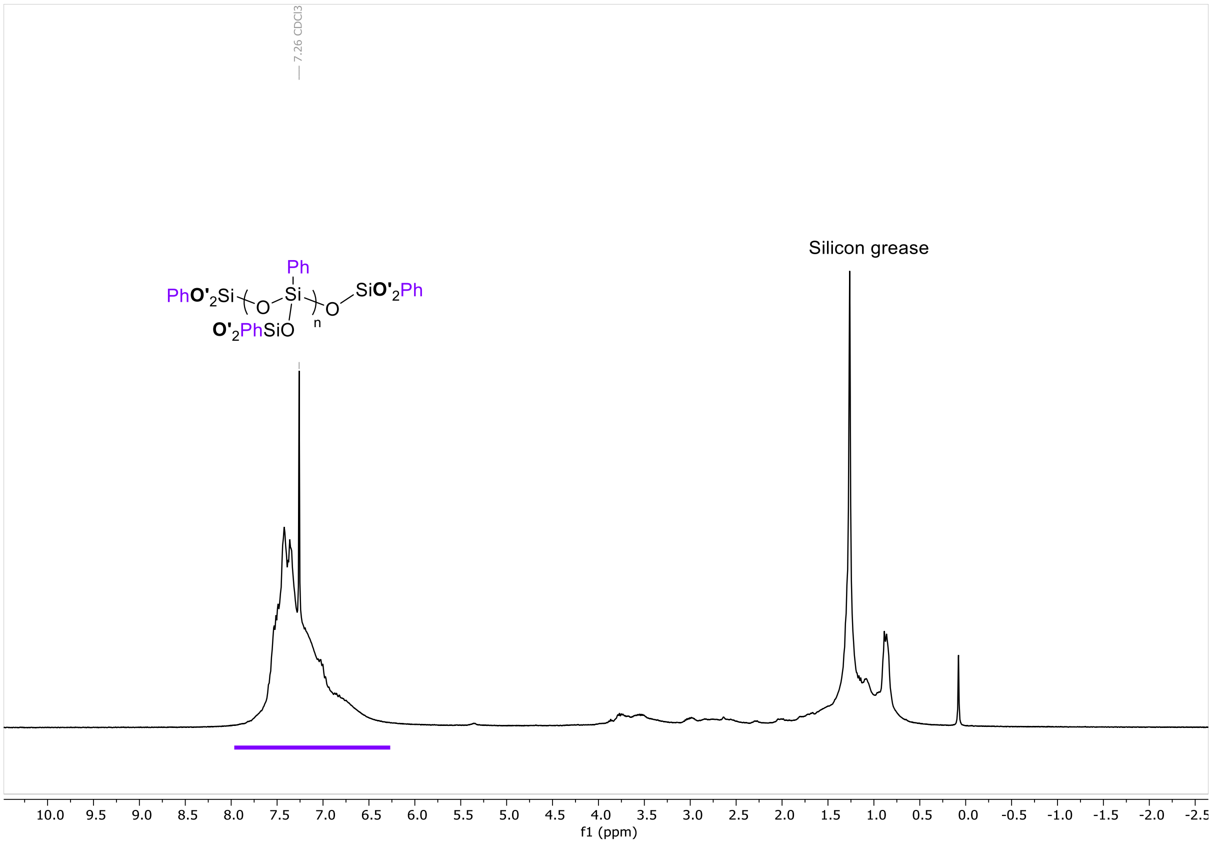

Figure S9: ^{1}H NMR (400 MHz, $CDCl_3$, 296 K) spectrum of **Si-3** after isolation via Kugelrohr distillation.

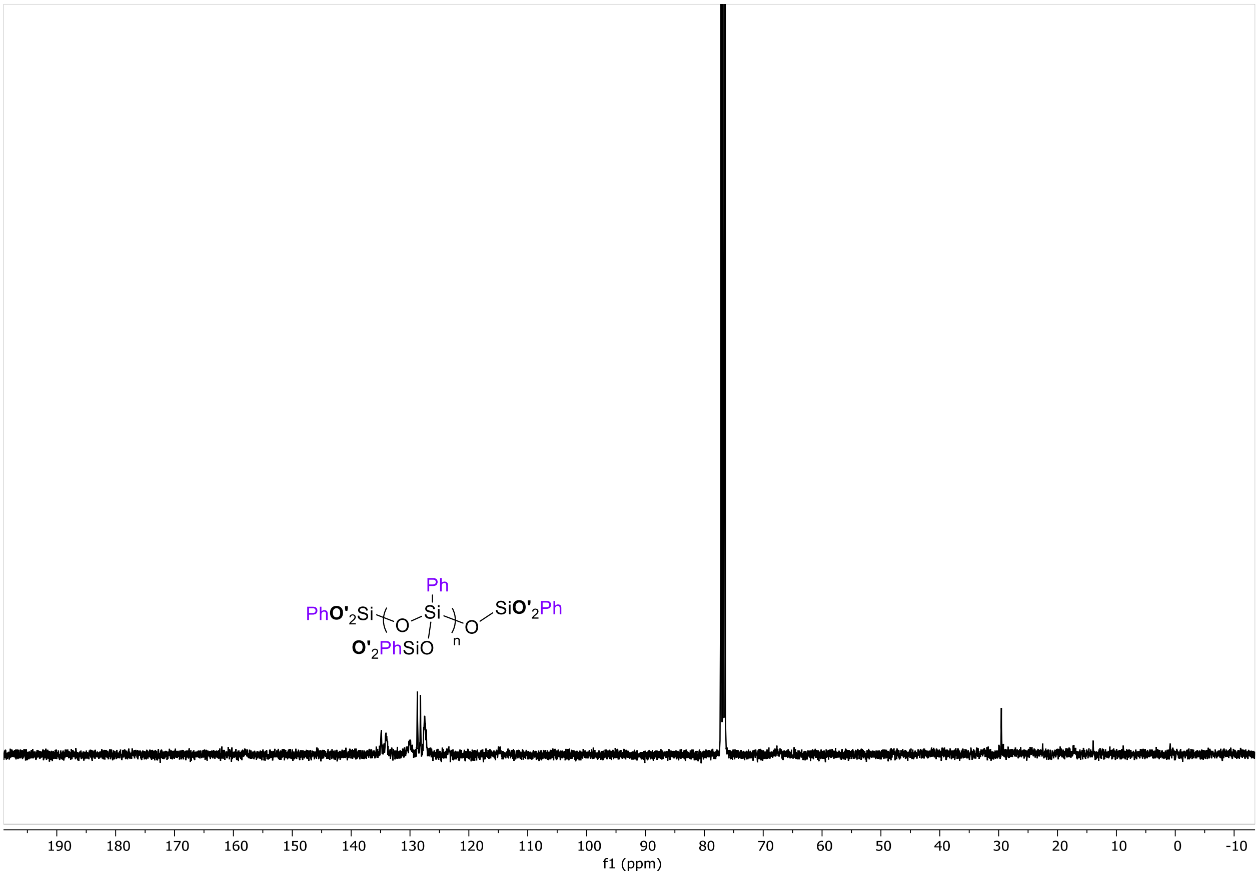

Figure S10: $^{13}C\{^{1}H\}$ **NMR (400 MHz, $CDCl_3$, 296 K)** spectrum of **Si-3** after isolation via Kugelrohr distillation.

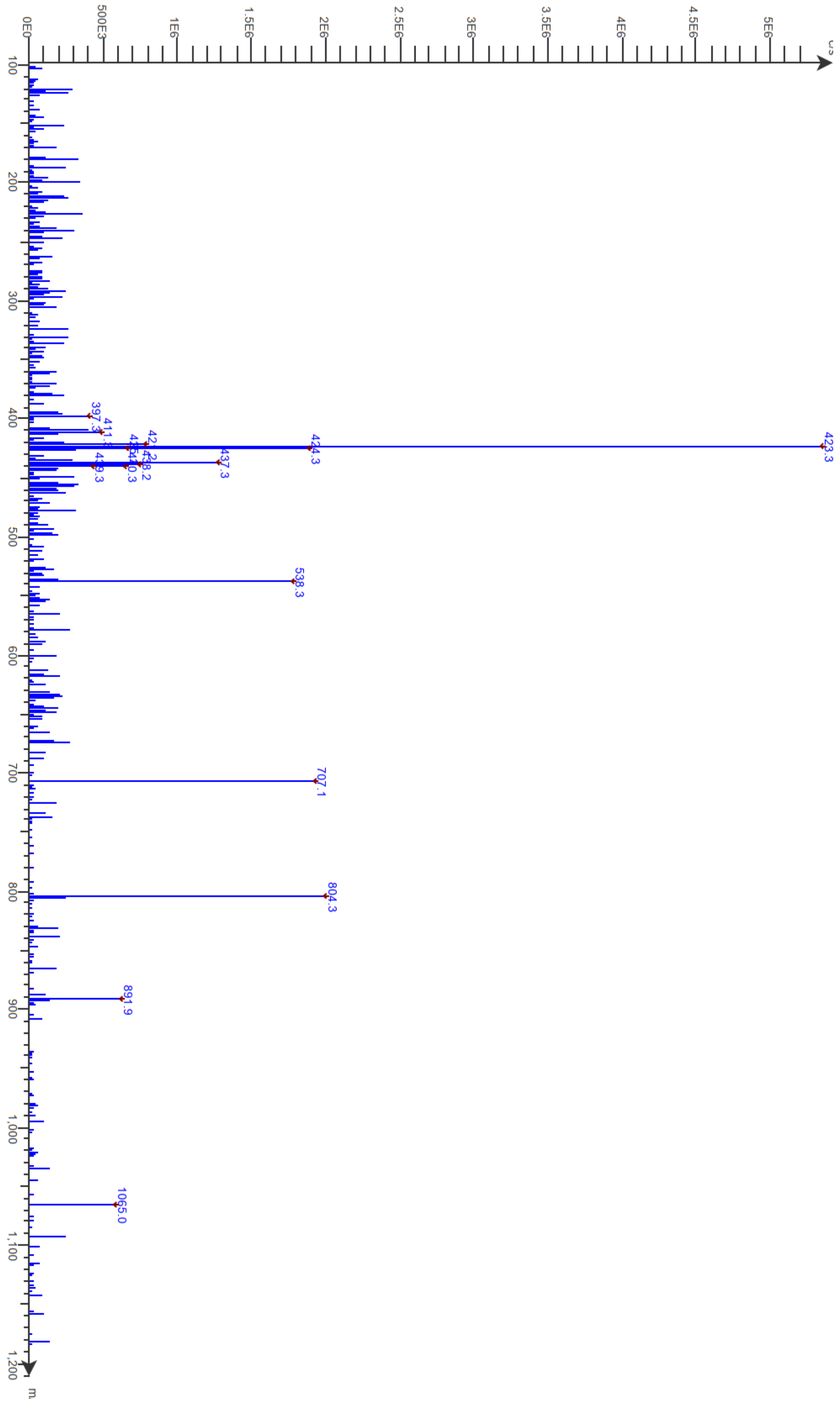

Graph S8 – Anionic APCI-MS of **Si-3** obtained after Kugelrohr distillation.

10.2. Procedure for the ^{1}H-NMR of the crude reaction mixture prior to hydrolysis

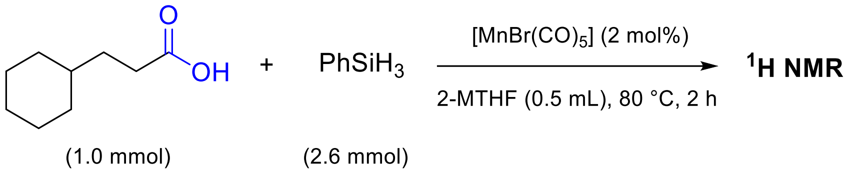

Scheme S27: Reaction between cyclohexanecarboxylic acid and $PhSiH_3$ performed to record crude ^{1}H-NMR of the reaction mixture before hydrolysis.

[MnBr(CO)$_5$] (5.7 mg, 0.02 mmol) was placed into a Schlenk tube equipped with a screw cap and containing a magnetic stirrer. The reaction vessel was evacuated and filled with argon three times. Cyclohexanepropanoic acid (163.3 mg, 1.0 mmol), $PhSiH_3$ (279.2 mg, 2.6 mmol) and 2-MTHF (0.5 mL) were added under argon. The mixture was heated at 80 °C for 2 hours. The reaction vessel was allowed to cool down to room temperature and brought into the glovebox. The crude reaction mixture (0.1 mL) was transferred into an oven-dried NMR tube, and dry and degassed $CDCl_3$ (0.4 mL) was added. A ^{1}H-NMR spectrum was then recorded at room temperature.

10.3. Procedure for the ^{19}F{^{1}H} NMR of the crude reaction mixture prior to hydrolysis

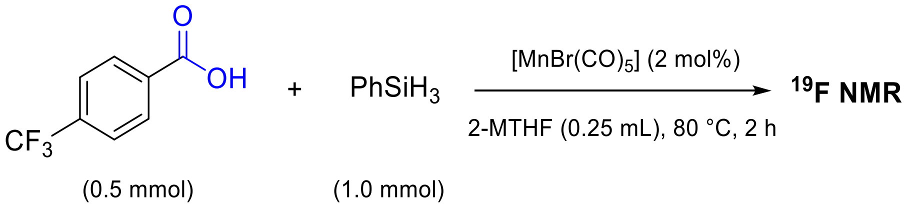

Scheme S28: Reaction between 4-Trifluoromethylbenzoic acid and $PhSiH_3$ performed to record crude ^{19}F{H}-NMR of the reaction mixture before hydrolysis.

[MnBr(CO)$_5$] (2.7 mg, 0.01 mmol) and 4-Trifluoromethylbenzoic acid (94.5 mg, 0.5 mmol) were placed into a Schlenk tube equipped with a screw cap and containing a magnetic stirrer. The reaction vessel was evacuated and filled with argon three times. $PhSiH_3$ (108.2 mg, 1.0 mmol) and 2-MTHF (0.25 mL) were added under argon flush. The mixture was heated at 80 °C for 2 hours. The reaction vessel was allowed to cool down to room temperature and brought into the glovebox. The crude reaction mixture (0.2 mL) was transferred into an oven-dried NMR tube, and dry and degassed THF-d_8 (0.3 mL) was added. A ^{19}F-NMR spectrum was then recorded at room temperature.

10.4. Procedure for the isolation of Si-3

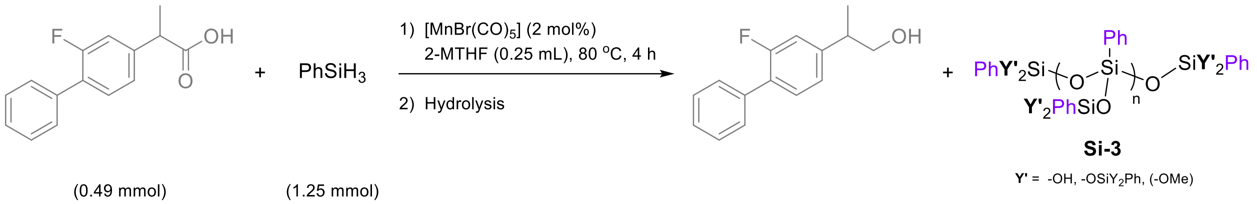

Scheme S29: Reaction between flurbiprofen acid and $PhSiH_3$ was performed to record ^{1}H NMR and APCI-MS spectra.

Flurbiprofen (118.6 mg, 0.49 mmol) was reacted under the same conditions used for substrates of type (a) (Section 3), with the respective amounts of $[MnBr(CO)_5]$ (2.8 mg, 0.01 mmol) and $PhSiH_3$ (135.9 mg, 1.26 mmol). After performing a bulb to bulb distillation and separating the product, the distillation (**Si-3**) residue was dissolved in DCM (0.5 mL), transferred in a vial, and dried under reduced pressure. A ^{1}H-NMR of the compound was recorded in $CDCl_3$. The anionic APCI-MS was recorded with a mass range from 100 to 1200 (Graph S8).

11. Chemoselectivity studies

As mentioned in the manuscript, some substrates were not successful in the reaction and did not afford the desired products. Here are reported the substrates that did not lead to the detection of any desired product, with an attempt to rationalize the fate of some notable examples.

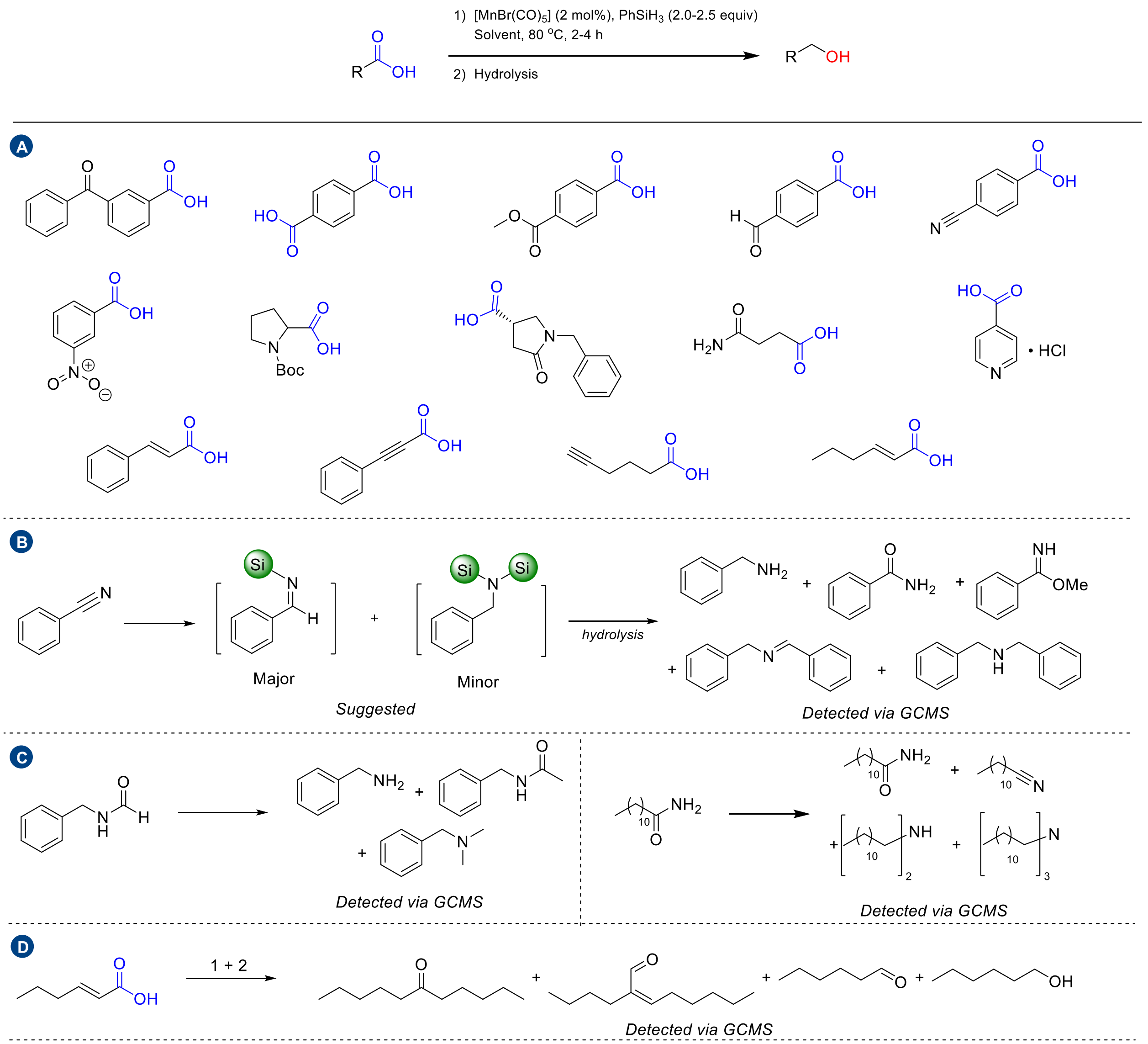

Figure S11 – Chemoselectivity studies performed on multiple substrates are shown. Panel A: List of several carboxylic acids that did not afford any product. Panel B: Reaction on benzonitrile performed using general procedure [b] (section 3.2) to afford multiple products detected by GC-MS. Panel C: Reaction on N-Benzyl formamide and lauramide were performed respectively according to the general procedure [b] and [a] (Section 3.2) to afford multiple products detected by GC-MS. Panel D: Reaction on trans-2-hexenoic acid to afford multiple products detected via GC-MS (see section 11.3 for the procedure).

11.1. The case of some disubstituted carboxylic acids

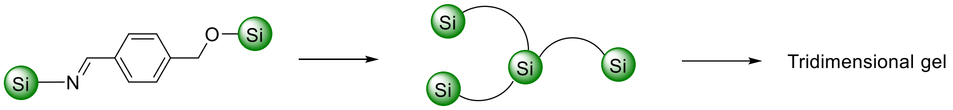

Figure S12 – Gel formation suggested as a possible reason for the low success in the reaction performed on some difunctional carboxylic acids.

As a result of the 4-cyanobenzoic acid not affording the desired product, benzonitrile was reacted using general procedure [b] (section 3.2), and the crude reaction mixture after hydrolysis has been analyzed by GC-MS. The results indicate that the nitrile is, to some extent, reduced in the presence of $PhSiH_3$ and $[MnBr(CO)_5]$ (Figure S11, section B). The activation of the nitrile group demonstrated on benzonitrile suggests the possibility for crosslinking by the difunctional, 4-cyanobenzoic acid when treated with $PhSiH_3$. It can be postulated that the crosslinking of substrates can form large tridimensional structures (Figure S12), resulting in gel-like substances—observed for this particular substrate and other difunctional carboxylic acids (shown in Figure S11, section A). In particular, terephthalic acid and 4-formylbenzoic acid were also observed to form a gel during the reaction, preventing complete homogenous mixing and limiting the progress of the reaction. This limitation is, to some extent, substrate (probably, more specifically *geometry*) dependent since levulinic acid (a different keto-acid) was successfully reduced to the respective diol in good yield.

11.2. Amide substituted carboxylic acids

The reaction of N-benzyl formamide and Lauramide were studied via GC-MS analysis after performing the general procedure [b] (section 3.2) and general procedure [a] (section 3.1), respectively. This allowed us to rationalize the result obtained from some amide-substituted carboxylic acid. It was shown that the amide group is also susceptible to reduction in the respective reaction conditions and that differently substituted amines are obtained after hydrolysis (shown in Figure S11, section C).

In the case of Boc-L-Proline, it does appear like the *boc* protection group is unstable in the reaction conditions, since upon hydrolysis, it was not possible to detect the protecting group anymore via ^{1}H-NMR nor via GC-MS.

11.3. α,β-Unsaturated carboxylic acids

The reaction of trans-2-hexenoic acid was studied via GC-MS analysis after hydrolyzing the crude reaction mixture. The general procedure [a] was followed (section 3.1) until hydrolysis, which used $CHCl_3$ as a solvent and the conditions from general procedure [c] (section 3.3)). It was shown that the substrate, very reactive in the reaction environment, formed multiple different products: fully reduced 1-hexanol; the

ketone, undecane-6-one; hexanal; and condensation product, 2-butyloct-2-enal. Condensation products may have formed in the hydrolysis step, performed in $CHCl_3$. Conjugated α,β-unsaturated carboxylic acids like trans-cinnamic acid did not afford any product. Therefore it seems reasonable to assume that, in general, the reaction is not tolerant towards α,β-unsaturated carboxylic acids.

12. Low Catalyst Loading Experiments on Phenylacetic acid

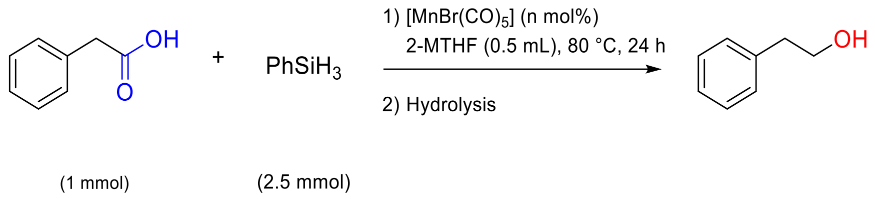

Scheme S30: Experiments at low catalyst loading in the case of phenylacetic acid.

Phenylacetic acid (1 mmol) was added to a Schlenk tube equipped with a screw cap and containing a magnetic stirrer. The reaction vessel was evacuated and filled with argon three times. Phenylsilane (270.5 mg, 2.5 mmol) was added under argon. The Schlenk tube was transferred into a glovebox, and an exact volume of $[MnBr(CO)_5]$ stock solution[3] (0.01 M in dry and degassed 2-MTHF) was added via Hamilton syringe. Then, additional dry and degassed 2-MTHF was added to the Schlenk tube to equate to 0.5 mL per 1 mmol of acid. The mixture was heated at 80 °C for 24 hours. After cooling down the reaction vessel to room temperature, MeOH (6 mL) and an aqueous NaOH solution (10% w/w, 4 mL) were added dropwise. Then, the reaction mixture was stirred overnight. Before extraction, ferrocene (37.2 mg, 0.2 mmol) was dissolved in DCM (4 mL) and added as an internal. The crude product was then extracted from the aqueous phase with DCM (3 × 10 mL). The combined organic layers were dried over $MgSO_4$, filtered, and the solvents removed under reduced pressure. NMR yields were obtained by setting the integral value for the ferrocene singlet (4.16 ppm, 10 H) as 1.00 and integrating the characteristic methylene R-**CH_2**-CH_2OH signal of the respective product (typically ≈2.9 ppm, 2H). The results of the reactions are reported in Table S11.

[3]Prepared inside the glovebox in a 2 mL volumetric flask

Stock Solution Vol.	Catalyst Loading	Yield, %	TON
500 μL	0.5 mol%	78	156
100 μL	0.1 mol%	1	N/A

Table S11 – NMR yields for the low catalyst loading experiments performed on phenyl acetic acid. Yields were determined by ^{1}H NMR with respect to an internal standard, Ferrocene.

13. NMR Spectra

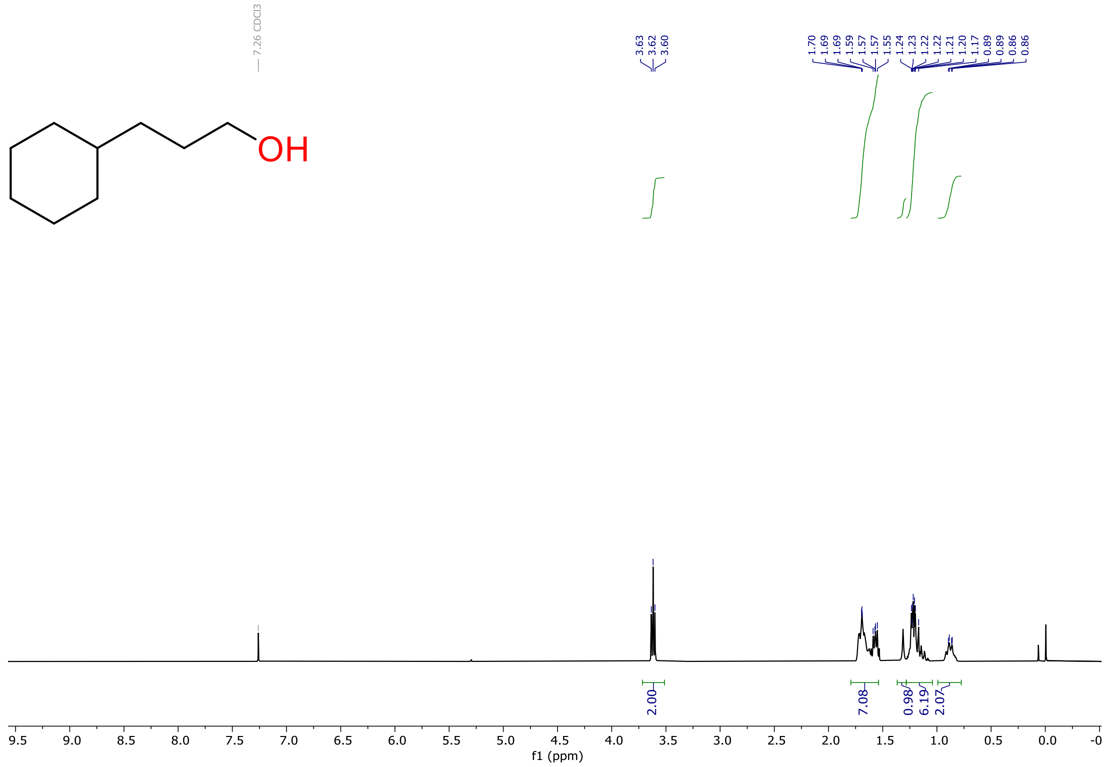

Figure S13 – **^{1}H NMR (400 MHz, $CDCl_3$, 296 K)** spectrum of compound **2**.

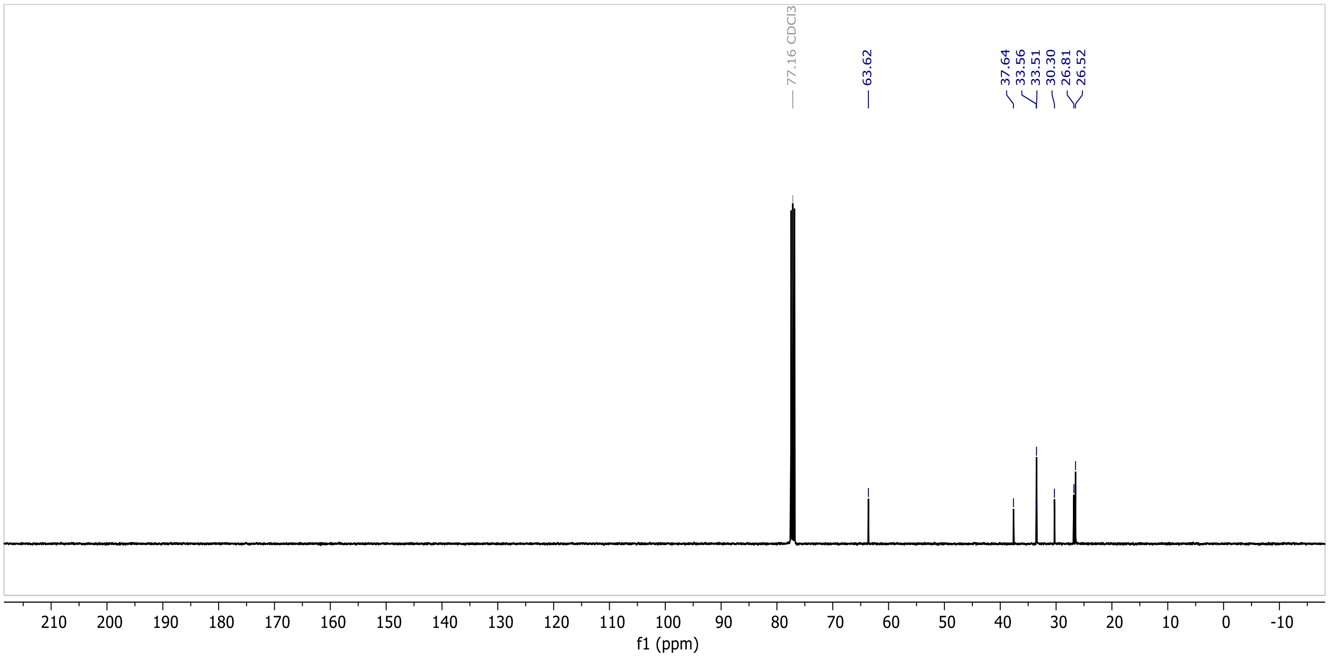

Figure S14 – **$^{13}C\{^1H\}$ NMR (101 MHz, $CDCl_3$, 296 K)** spectrum of compound **2**.

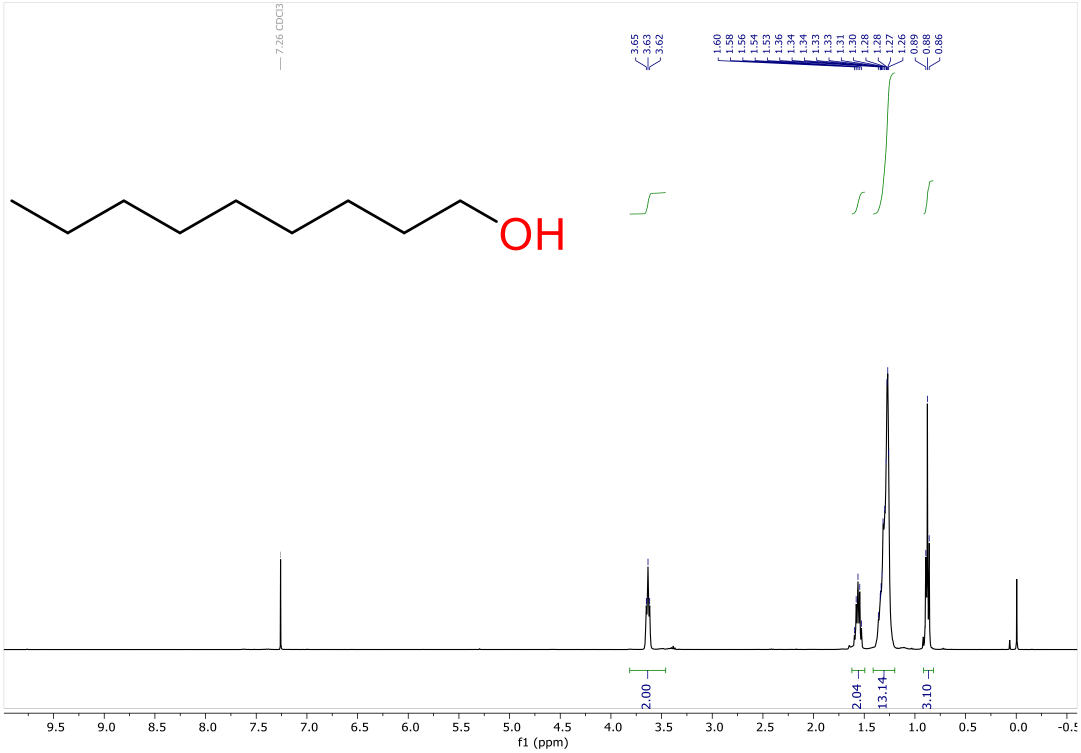

Figure S15 – **^{1}H NMR (400 MHz, $CDCl_3$, 296 K)** spectrum of compound **3**.

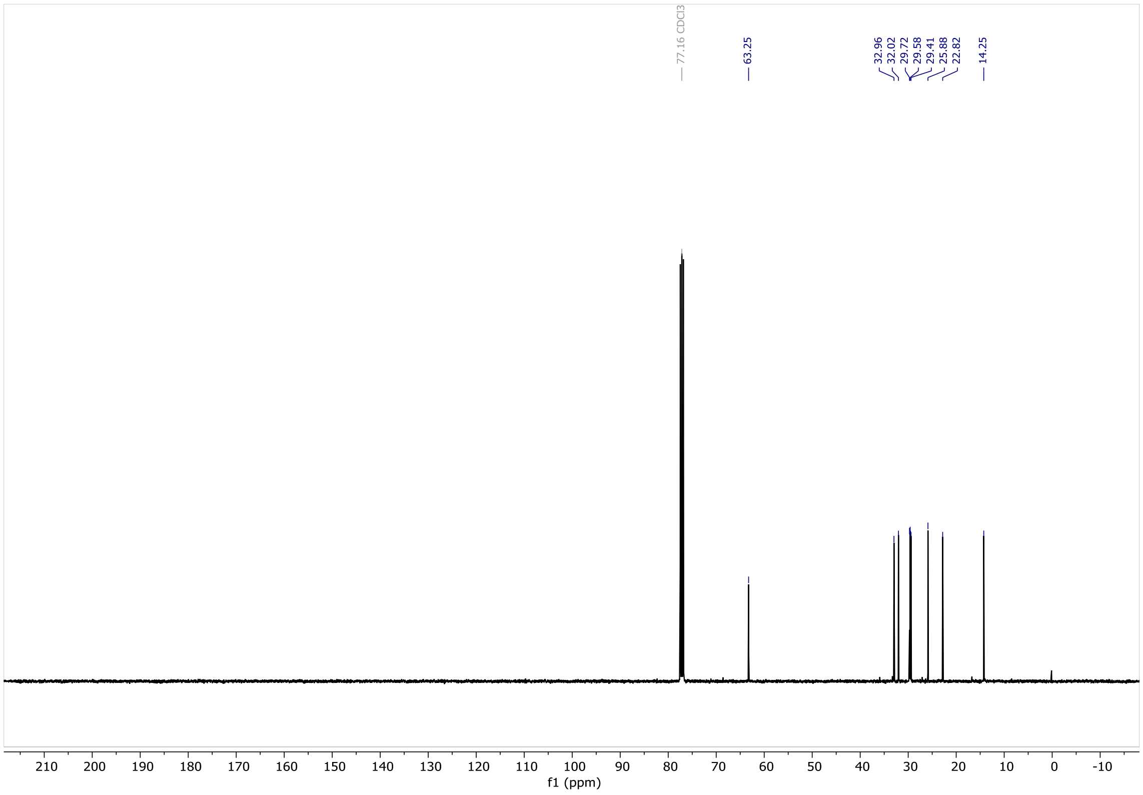

Figure S16 – **$^{13}C\{^1H\}$ NMR (101 MHz, $CDCl_3$, 296 K)** spectrum of compound **3**.

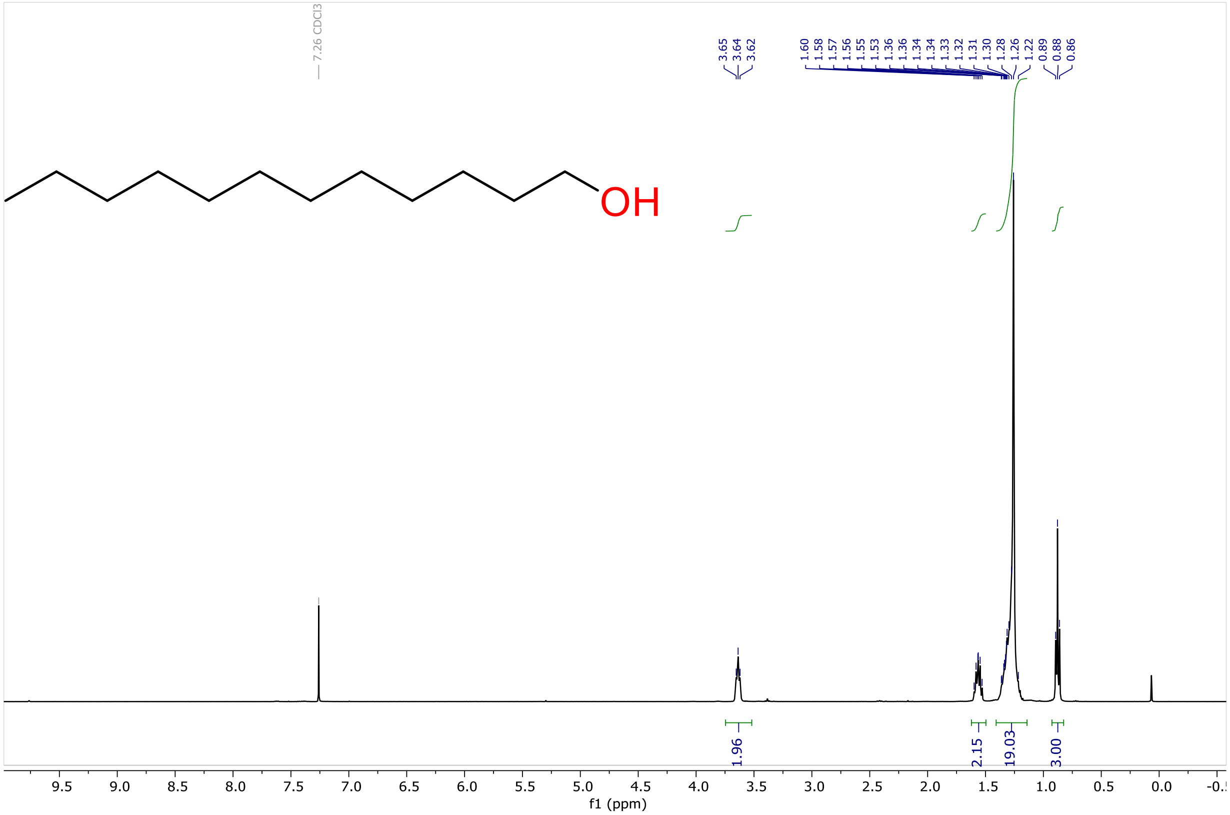

Figure S17 – **^{1}H NMR (400 MHz, $CDCl_3$, 296 K)** spectrum of compound **4**.

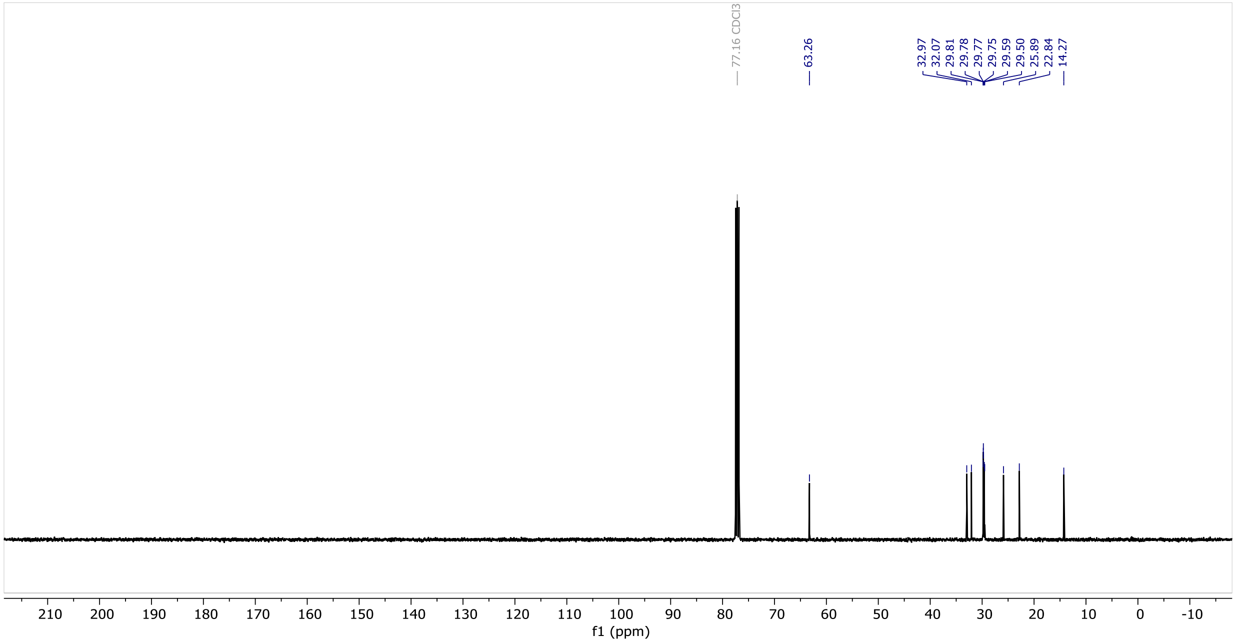

Figure S18 – **^{13}C{^{1}H} NMR (101 MHz, $CDCl_3$, 296 K)** spectrum of compound **4**.

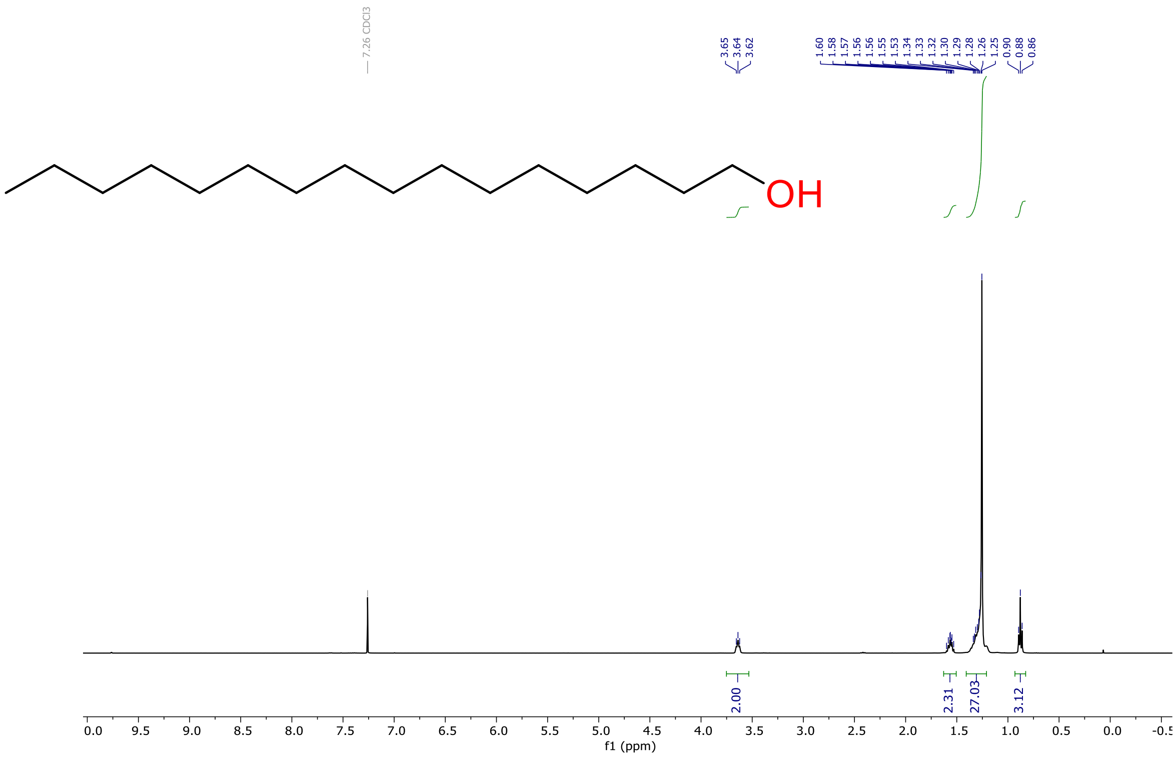

Figure S19 – **^{1}H NMR (400 MHz, $CDCl_3$, 296 K)** spectrum of compound **5**.

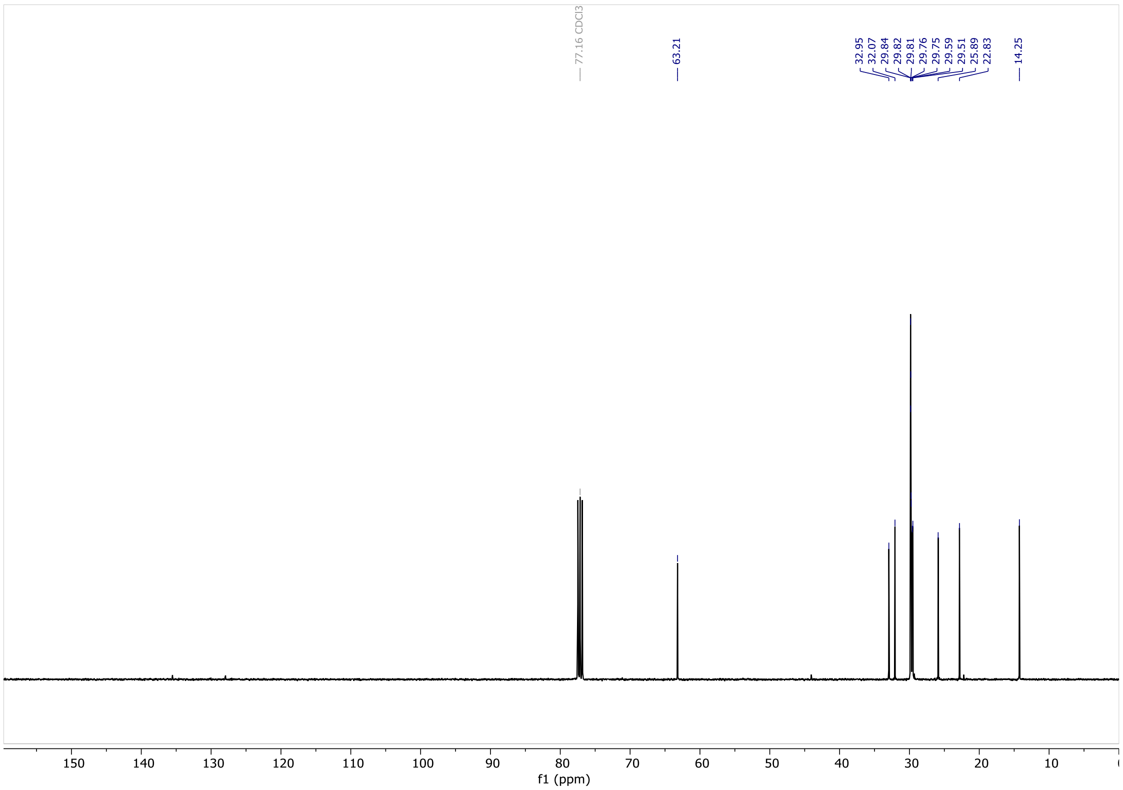

Figure S20 – **^{13}C{^{1}H} NMR (101 MHz, $CDCl_3$, 297 K)** spectrum of compound **5**.

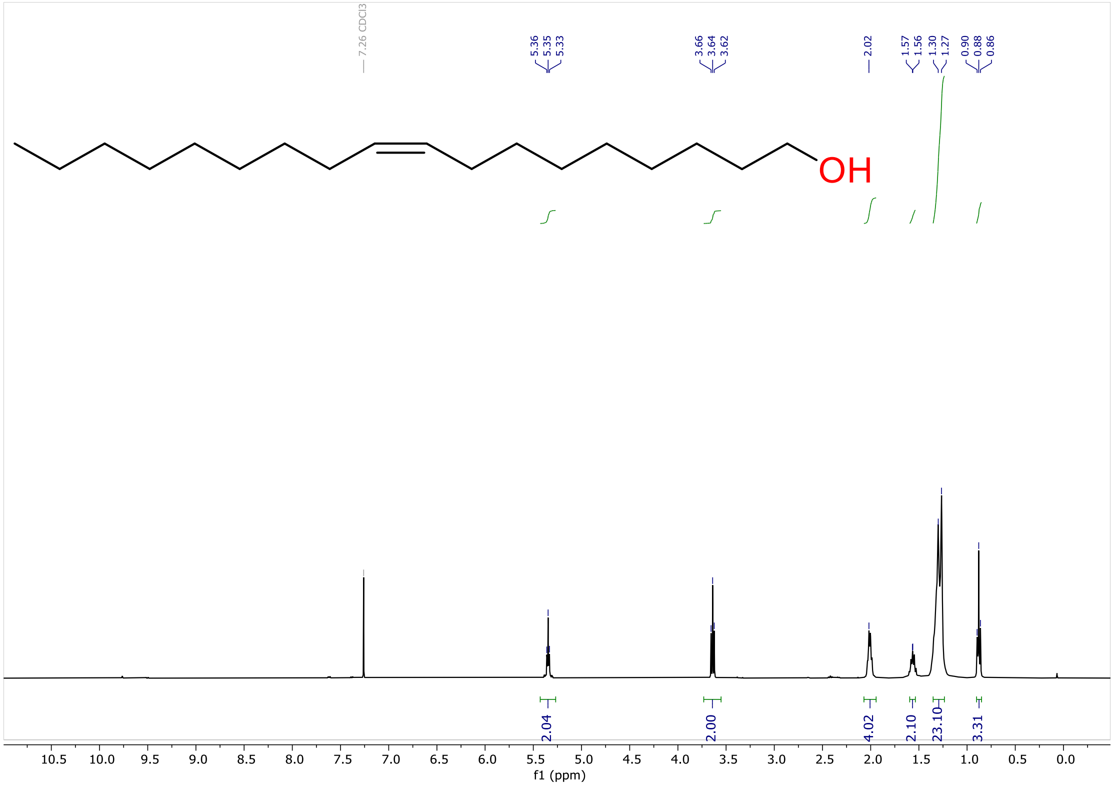

Figure S21 – **^{1}H NMR (400 MHz, $CDCl_3$, 296 K)** spectrum of compound **6**.

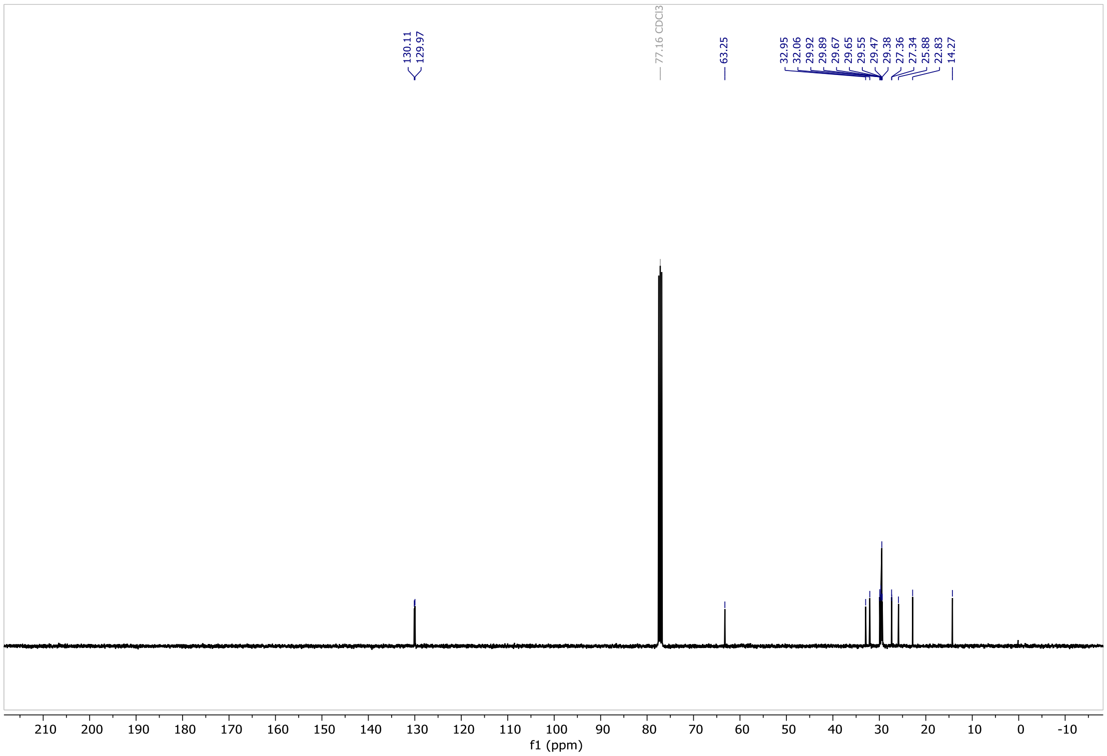

Figure S22 – **^{13}C{^{1}H} NMR (101 MHz, $CDCl_3$, 296 K)** spectrum of compound **6**.

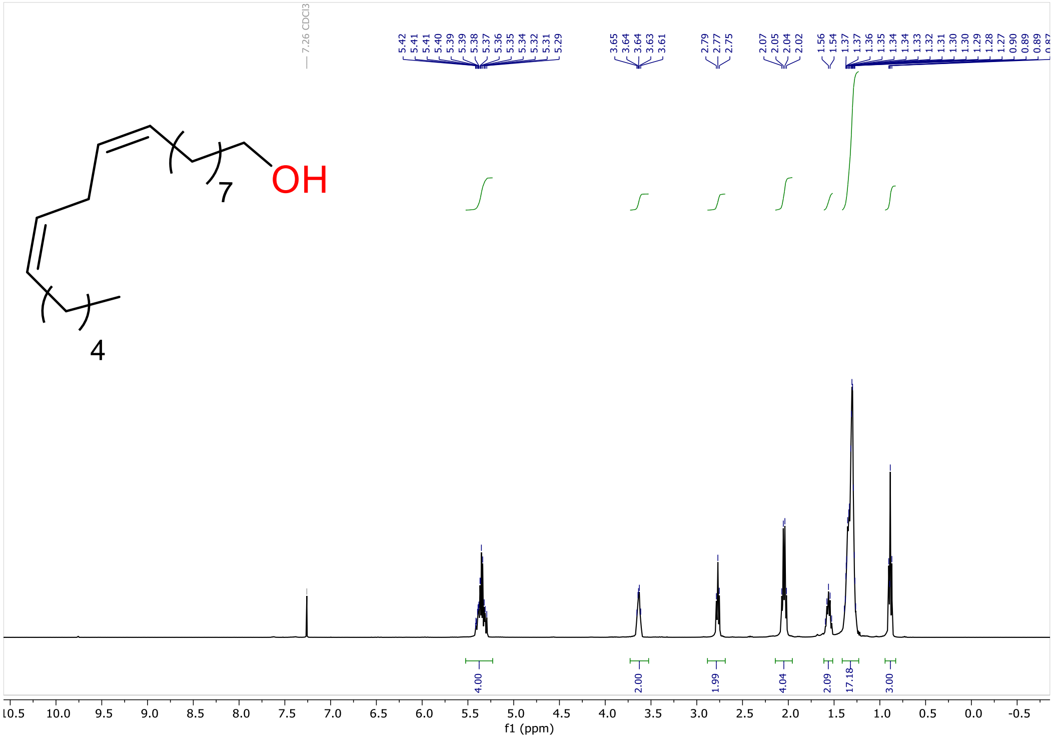

Figure S23 – **^{1}H NMR (400 MHz, $CDCl_3$, 296 K**) spectrum of compound **7**.

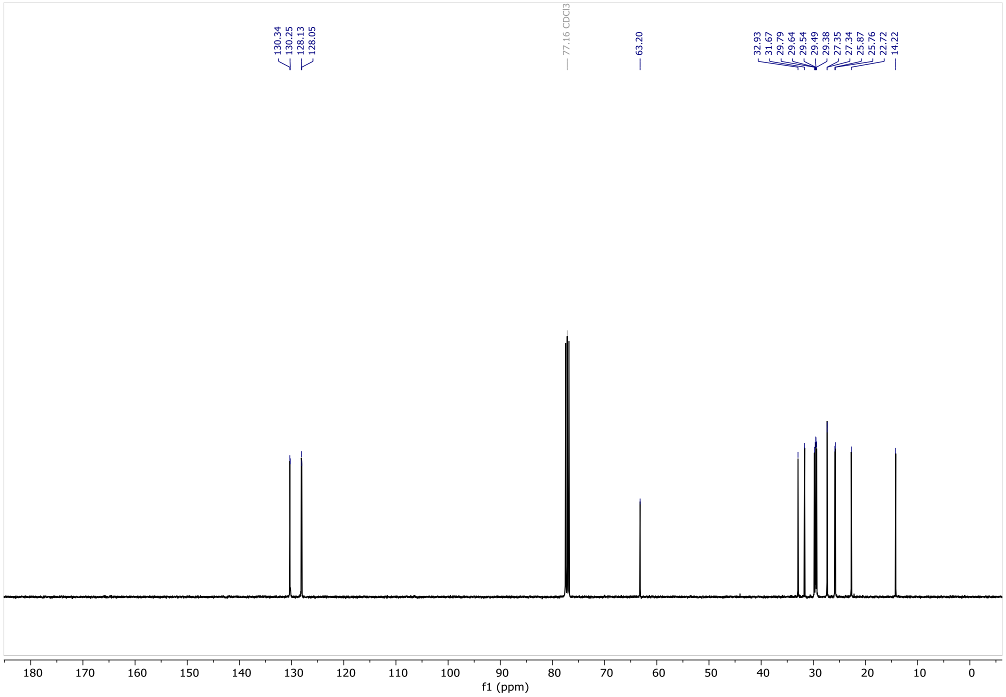

Figure S24 – **^{13}C{^{1}H} NMR (101 MHz, $CDCl_3$, 296 K)** spectrum of compound **7**.

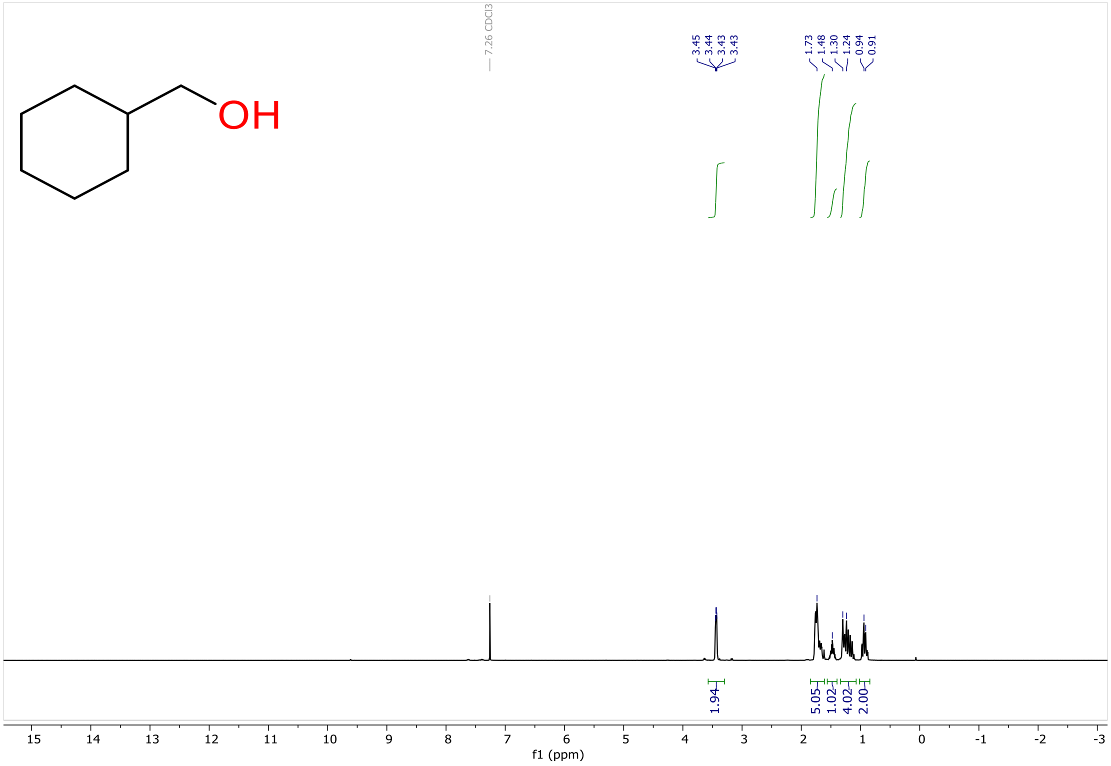

Figure S25 – **^{1}H NMR (400 MHz, $CDCl_3$, 296 K)** spectrum of compound **8**.

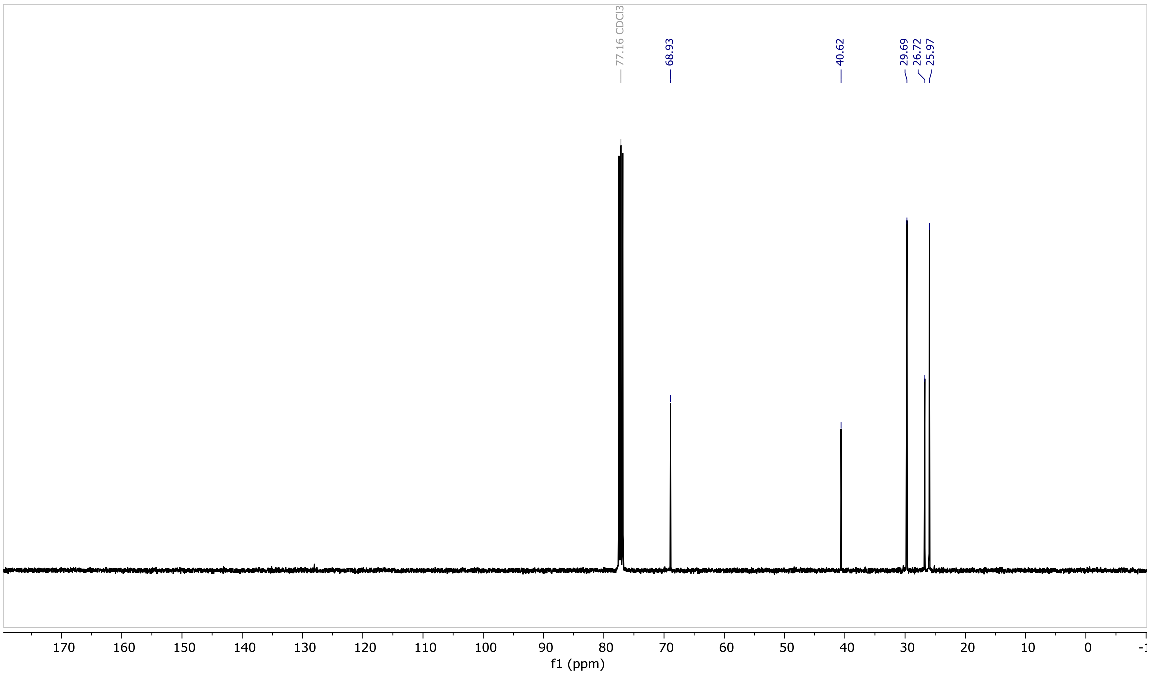

Figure S26 – **$^{13}C\{^{1}H\}$ NMR (101 MHz, $CDCl_3$, 296 K)** spectrum of compound **8**.

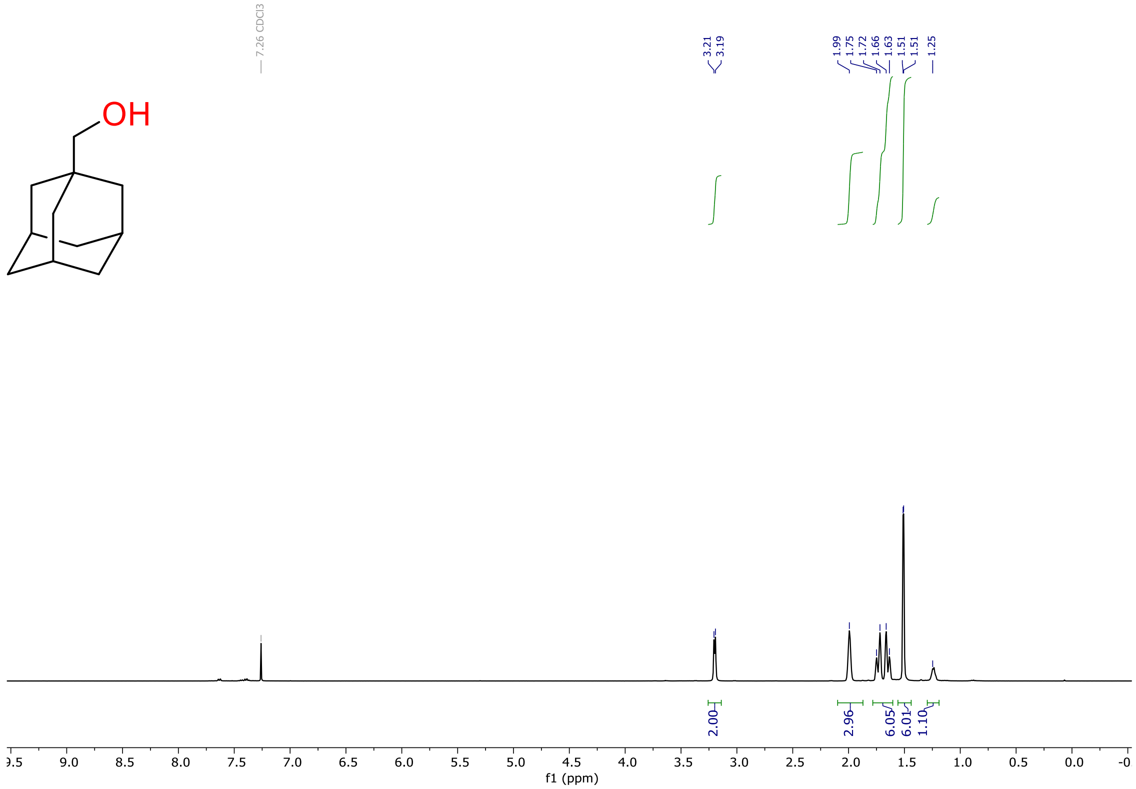

Figure S27 – ^{1}H NMR (400 MHz, $CDCl_3$, 296 K) spectrum of compound **9**.

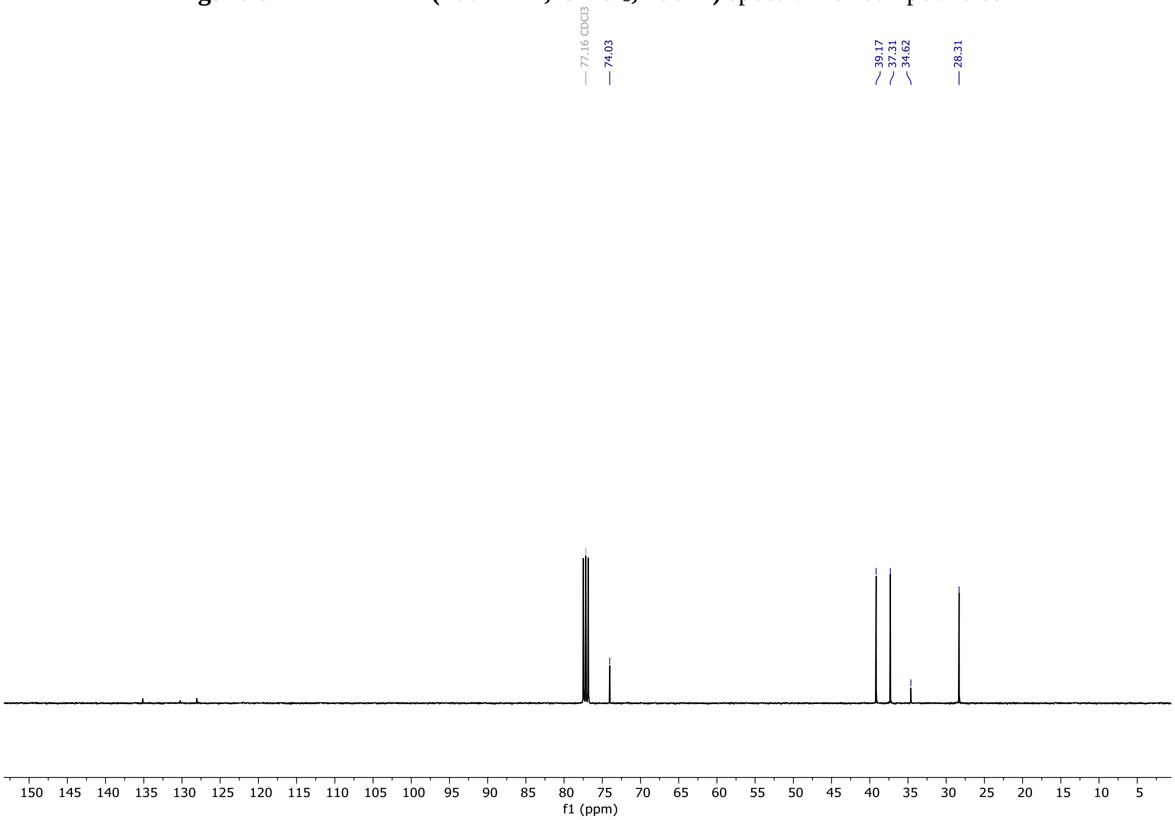

Figure S28 – ^{13}C{^{1}H} NMR (101 MHz, $CDCl_3$, 296 K) spectrum of compound **9**.

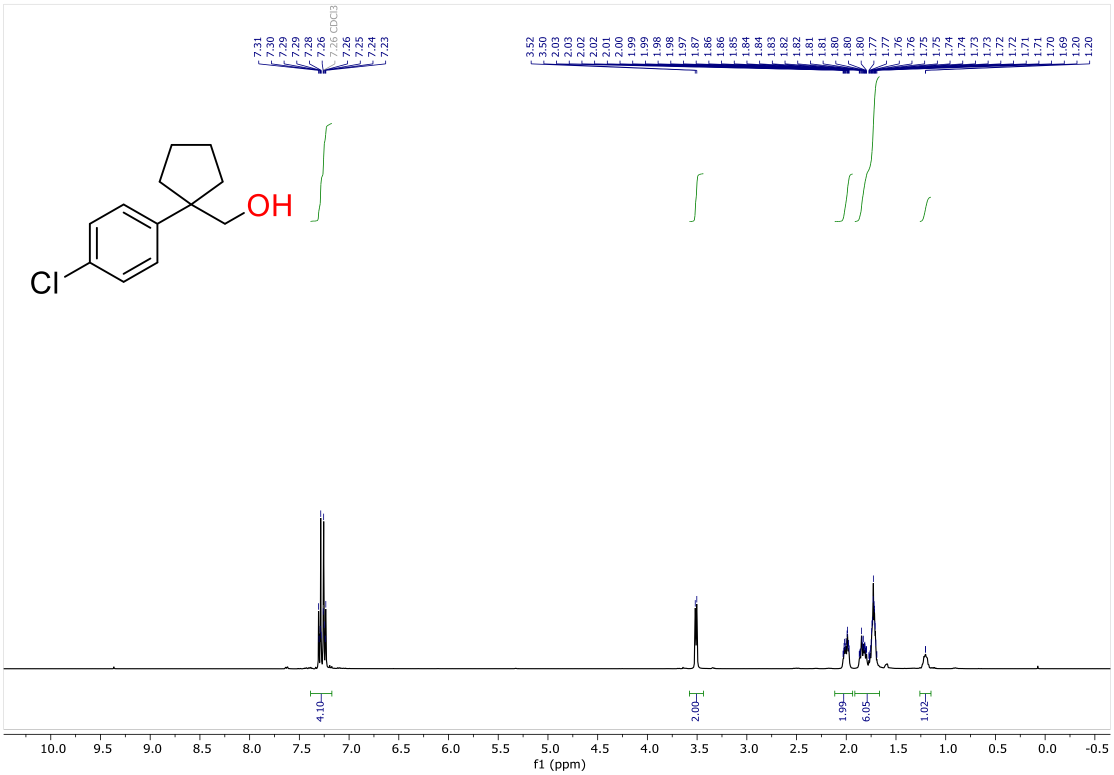

Figure S29 – **^{1}H NMR (400 MHz, CDCl$_3$, 296 K)** spectrum of compound **10**.

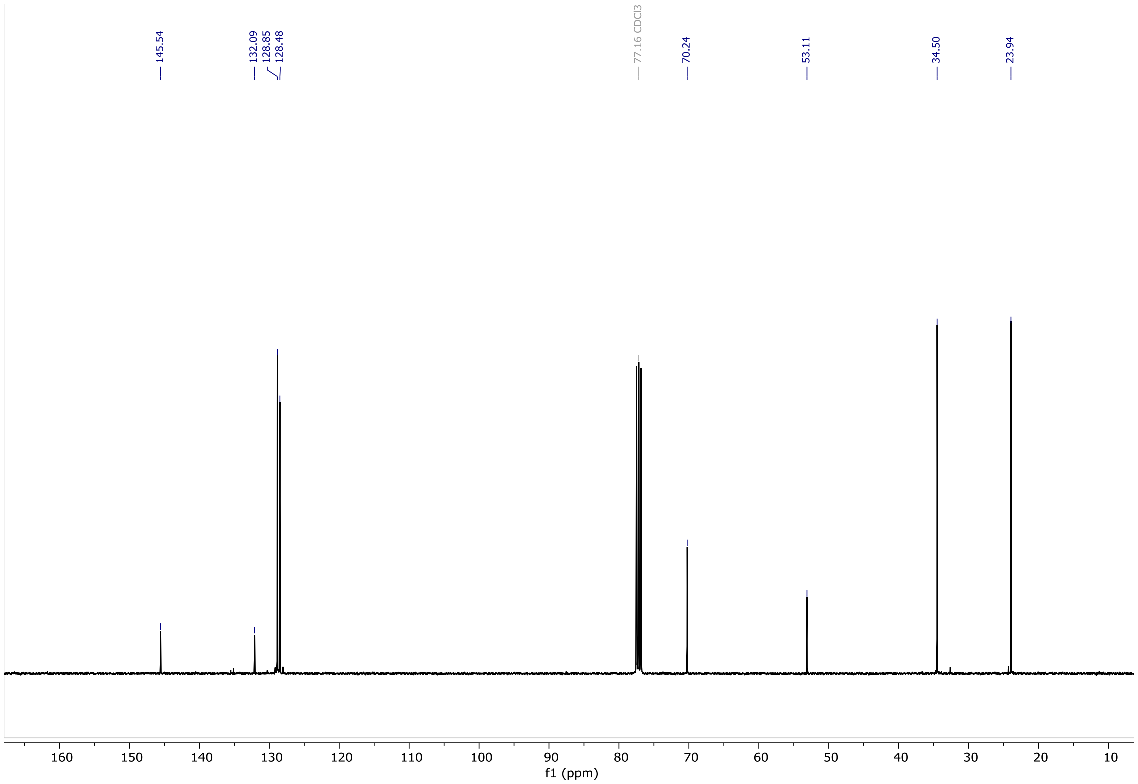

Figure S30 – **^{13}C{^{1}H} NMR (101 MHz, CDCl$_3$, 296 K)** spectrum of compound **10**.

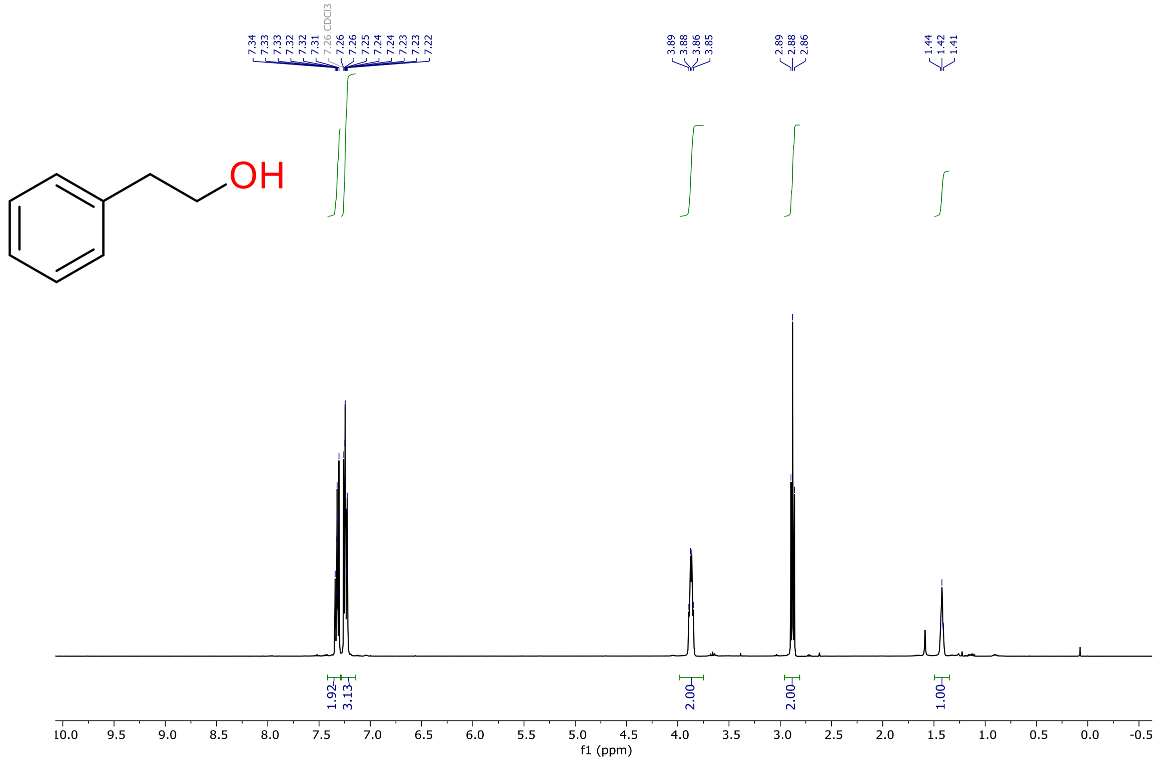

Figure S31 – **^{1}H NMR (400 MHz, $CDCl_3$, 296 K)** spectrum of compound **11**.

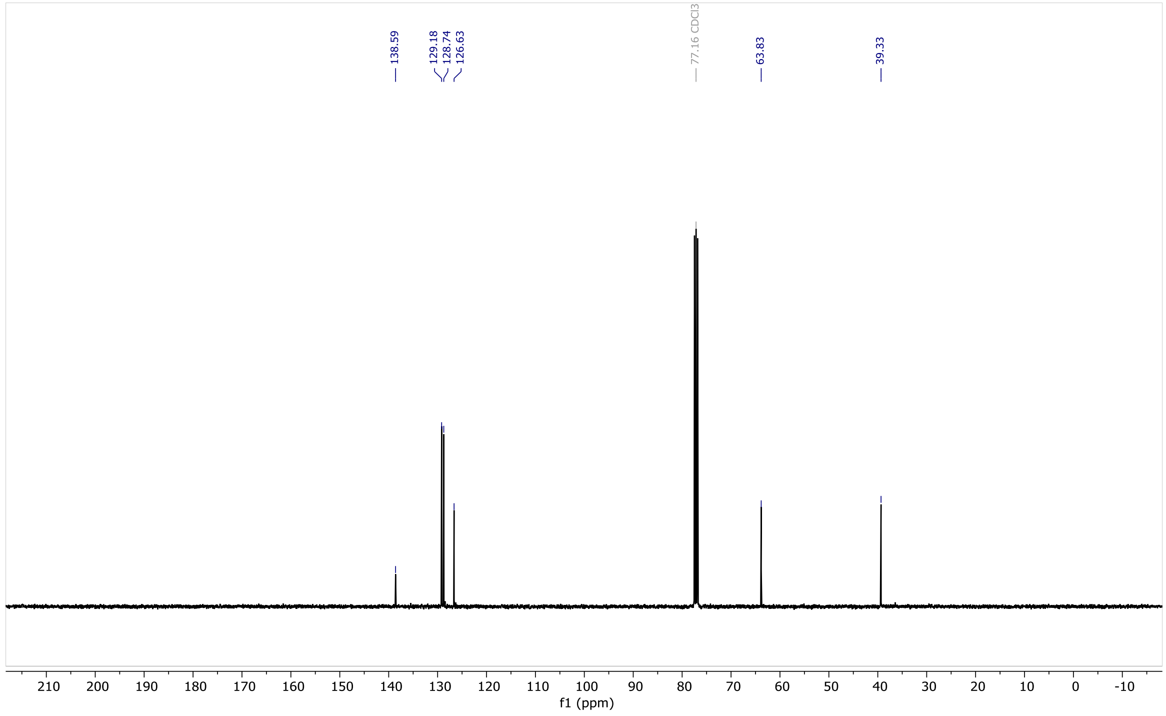

Figure S32 – **$^{13}C\{^{1}H\}$ NMR (101 MHz, $CDCl_3$, 296 K)** spectrum of compound **11**.

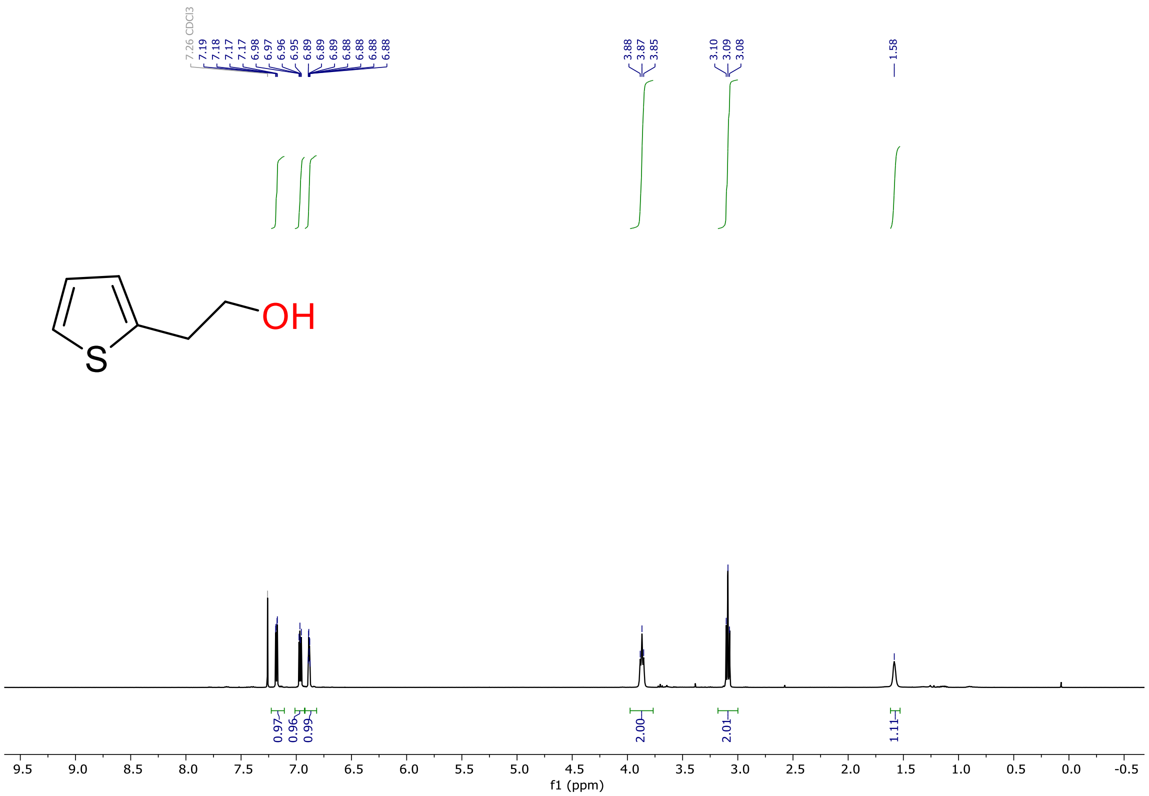

Figure S33 – **^{1}H NMR (400 MHz, $CDCl_3$, 296 K)** spectrum of compound **12**.

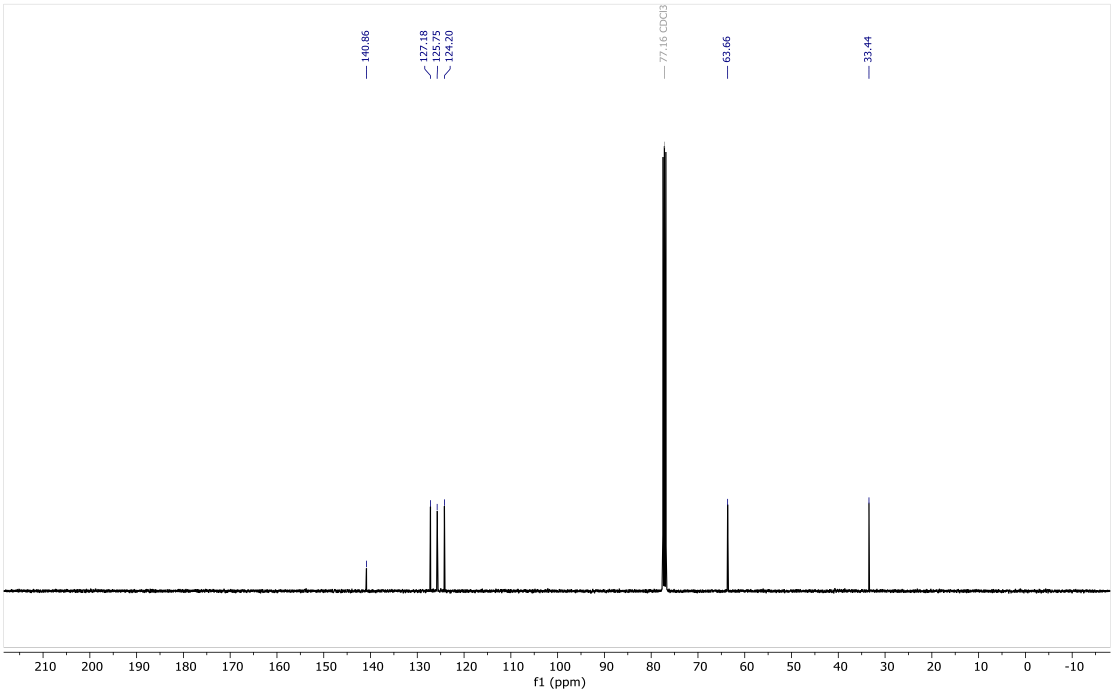

Figure S34 – **$^{13}C\{^{1}H\}$ NMR (101 MHz, $CDCl_3$, 296 K)** spectrum of compound **12**.

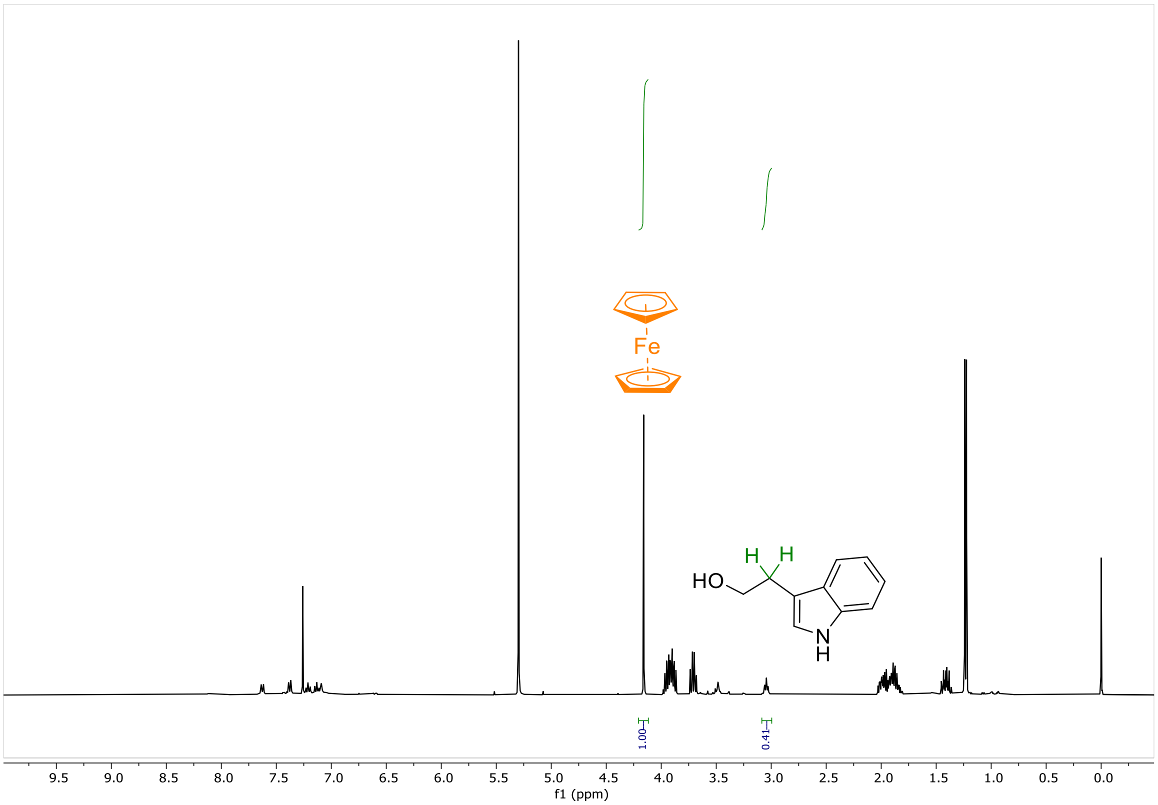

Figure S35 – ^{1}**H NMR (400 MHz, CDCl$_3$, 296 K)** spectrum of compound **13**.

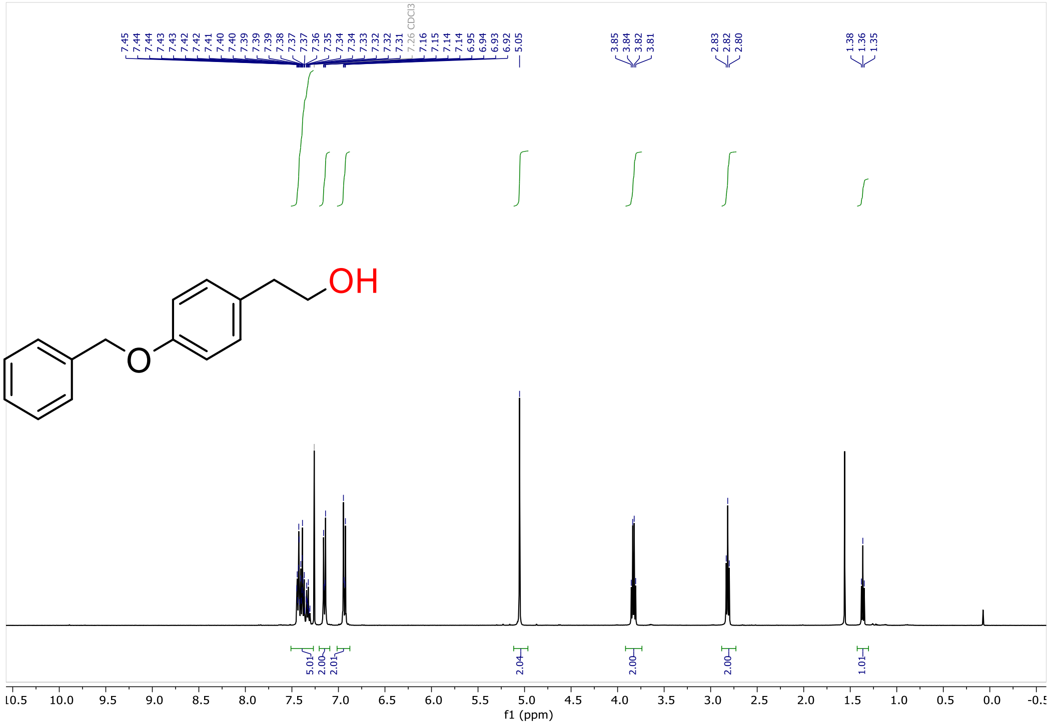

Figure S36 – ^{1}H NMR (400 MHz, $CDCl_3$, 296 K) spectrum of compound **14**.

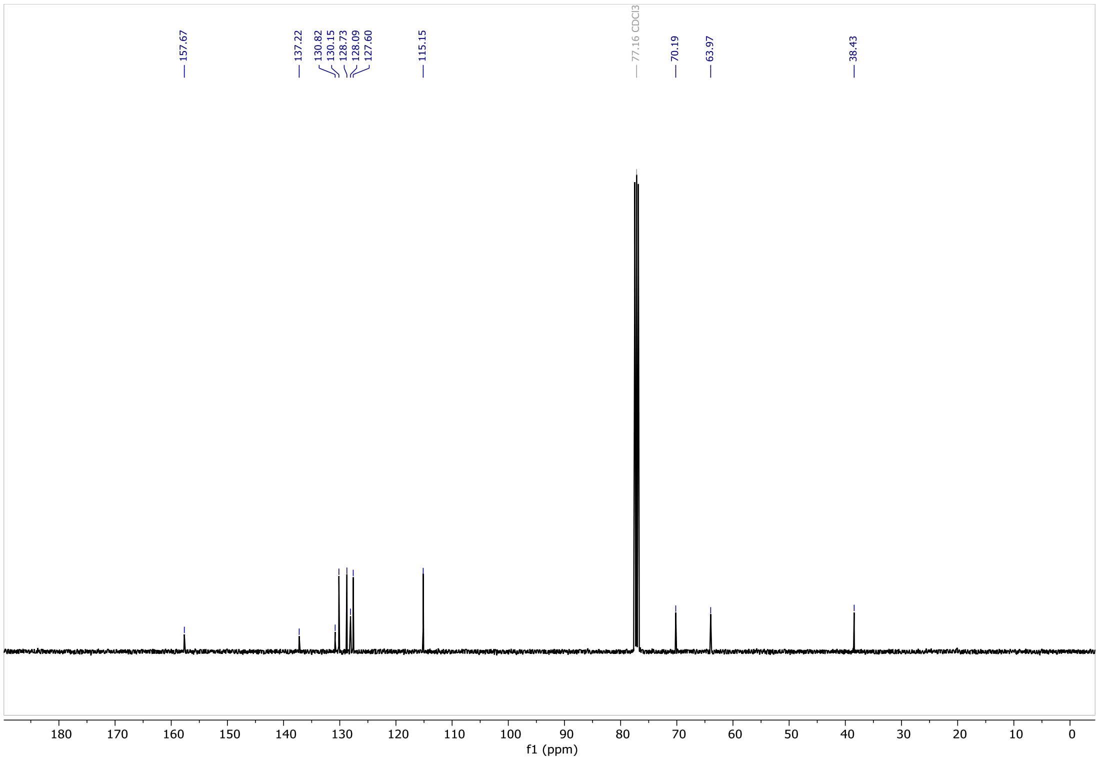

Figure S37 – $^{13}C\{^{1}H\}$ NMR (101 MHz, $CDCl_3$, 296 K) spectrum of compound **14**.

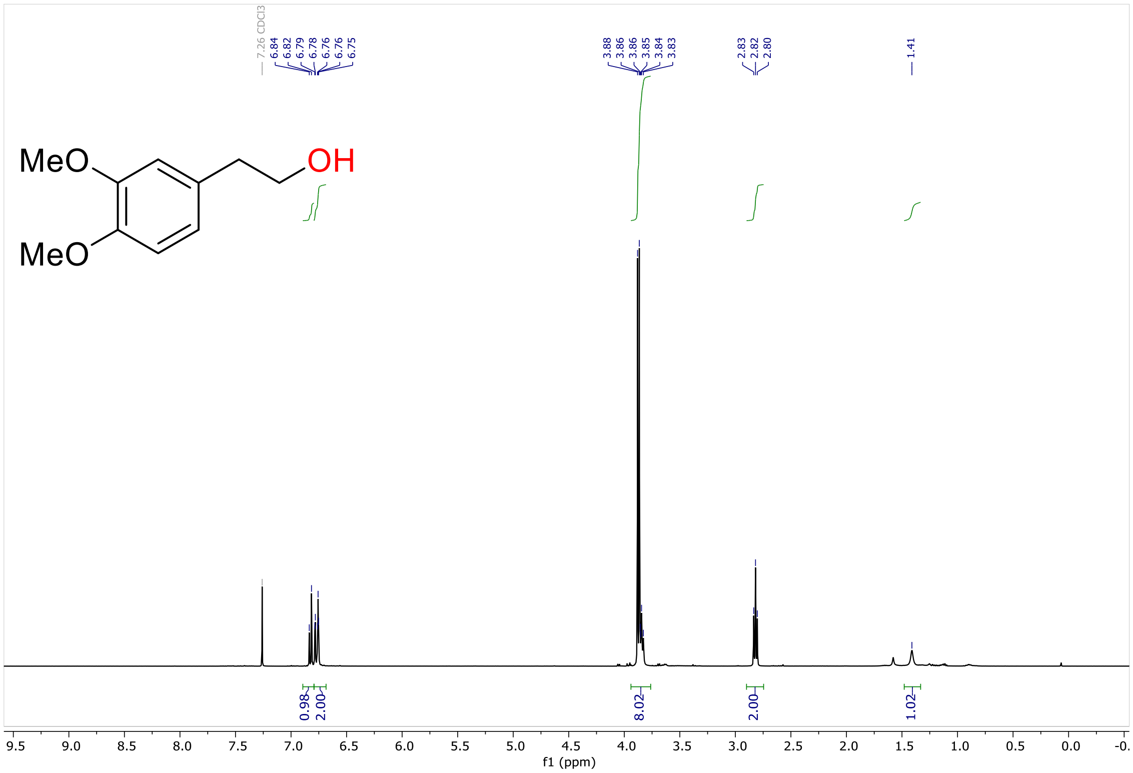

Figure S38 – [1]**H NMR (400 MHz, CDCl₃, 296 K)** spectrum of compound **15**.

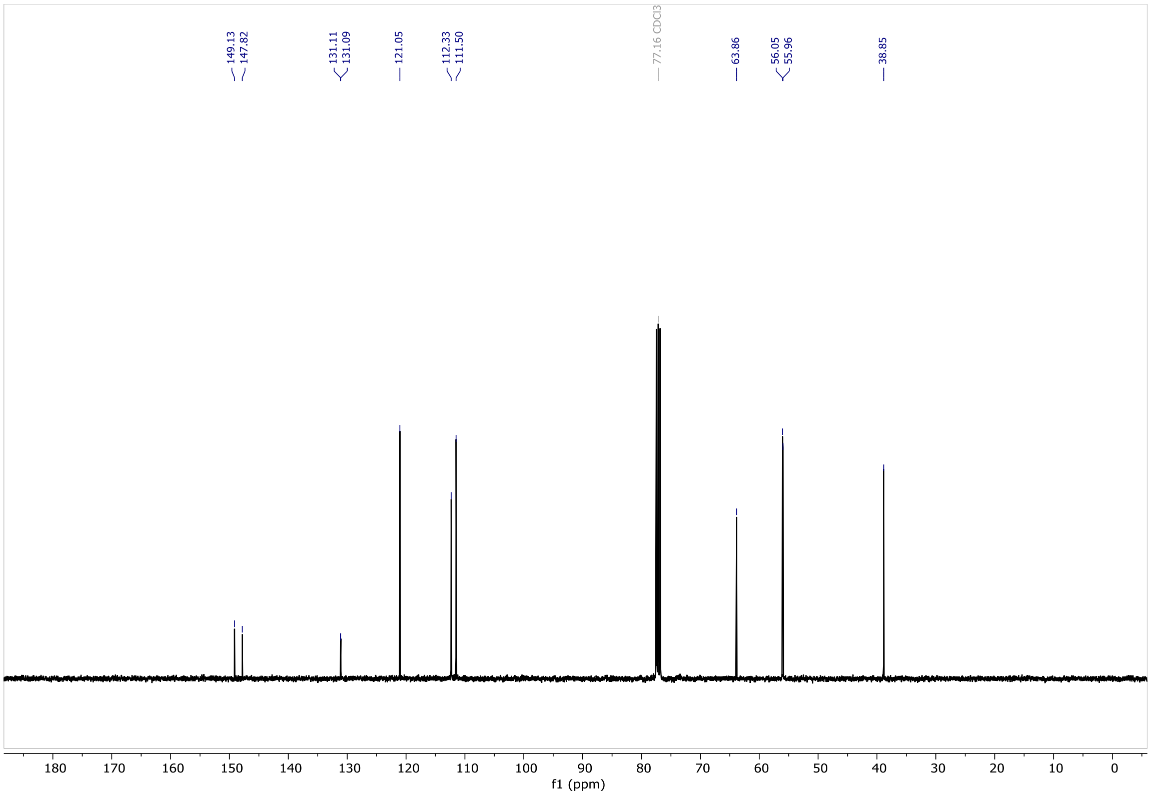

Figure S39 – **^{13}C{^{1}H} NMR (101 MHz, $CDCl_3$, 297 K)** spectrum of compound **15**.

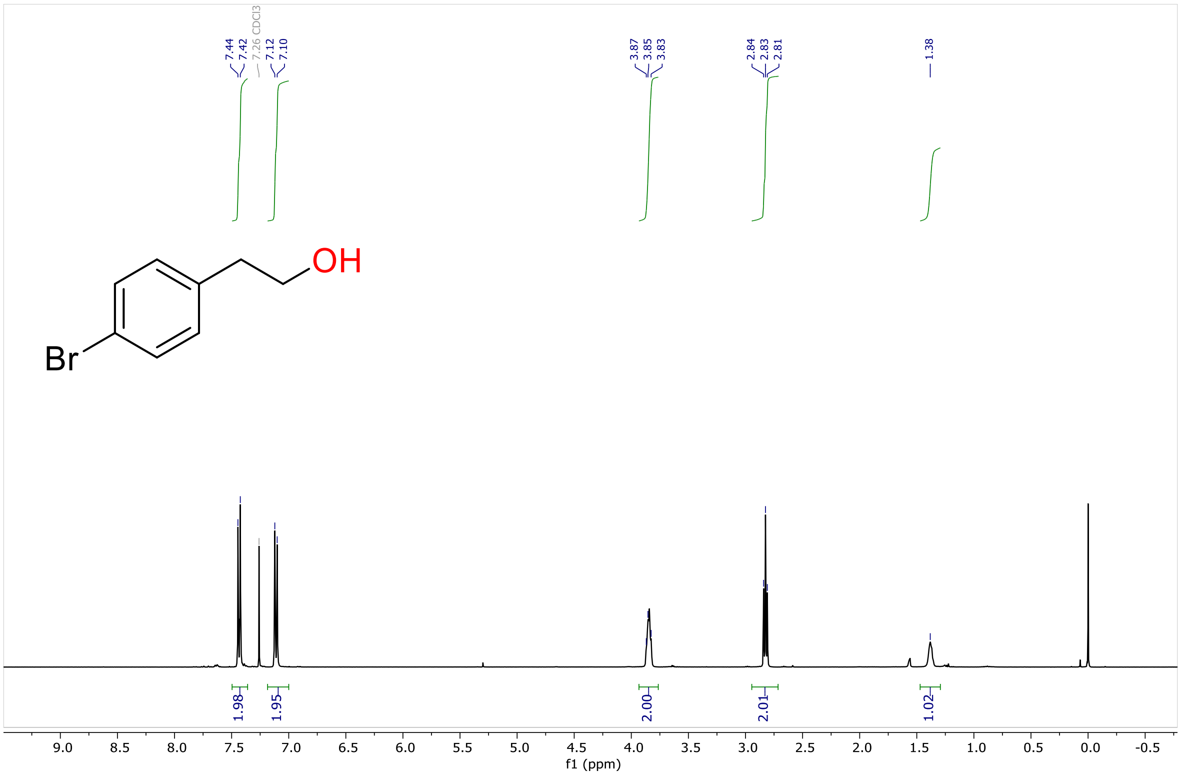

Figure S40 – **^{1}H NMR (400 MHz, $CDCl_3$, 296 K)** spectrum of compound **16**.

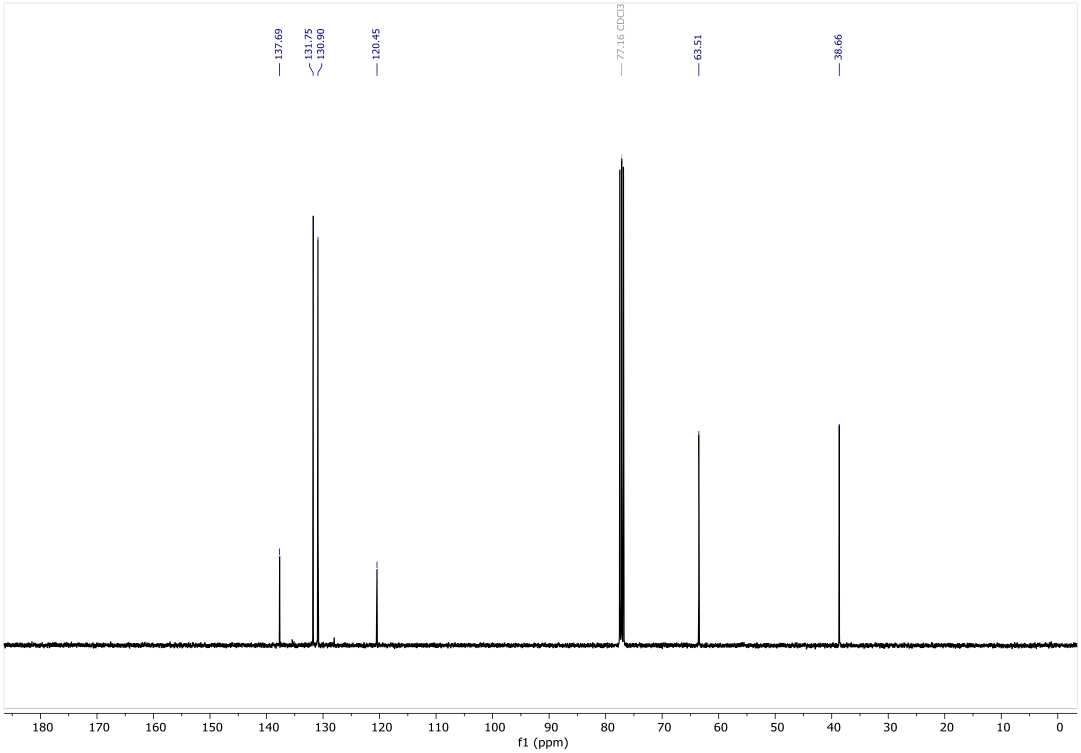

Figure S41 – **^{13}C{^{1}H} NMR (101 MHz, $CDCl_3$, 297 K)** spectrum of compound **16**.

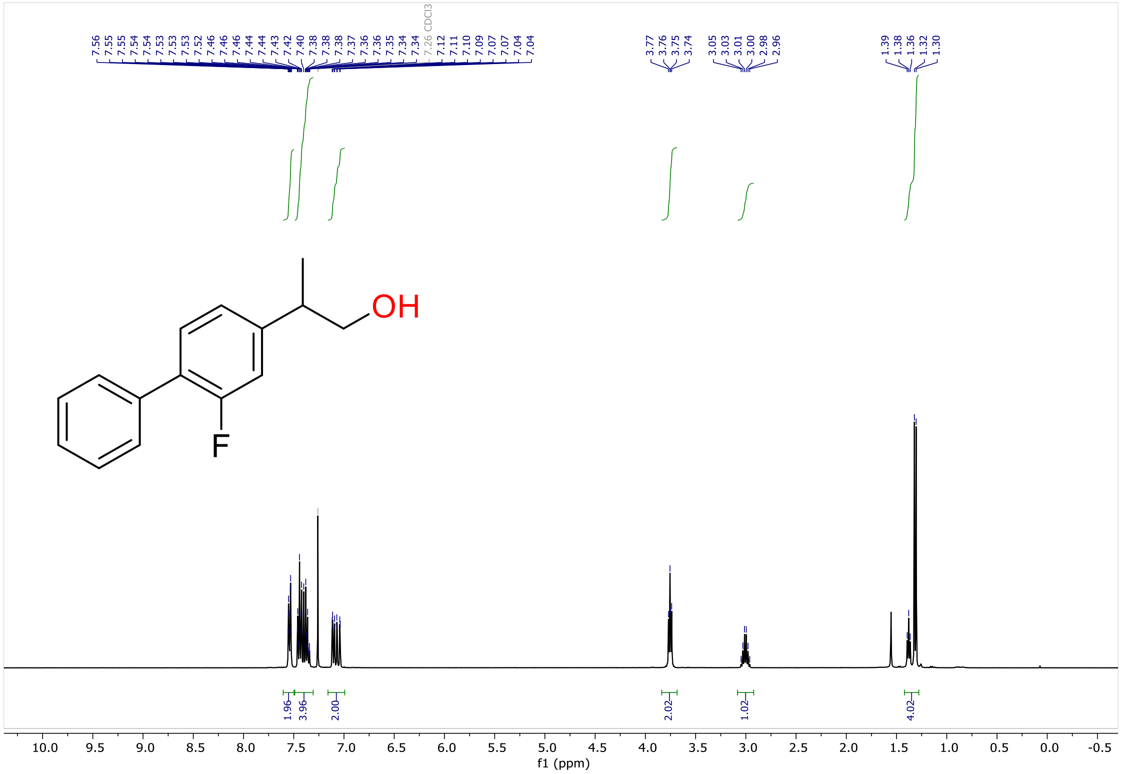

Figure S42 – ^{1}**H NMR (400 MHz, CDCl$_3$, 296 K)** spectrum of compound **17**.

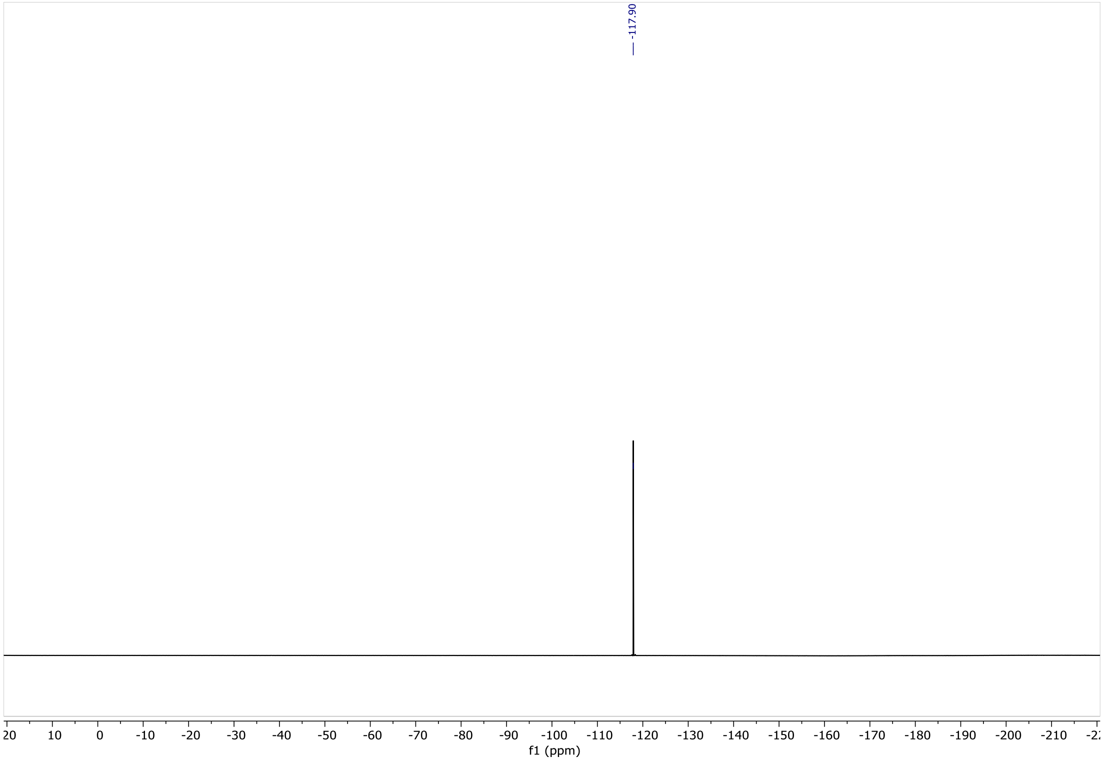

Figure S43 – ^{19}**F{^{1}H} NMR (376 MHz, CDCl$_3$, 296 K)** spectrum of compound **17**.

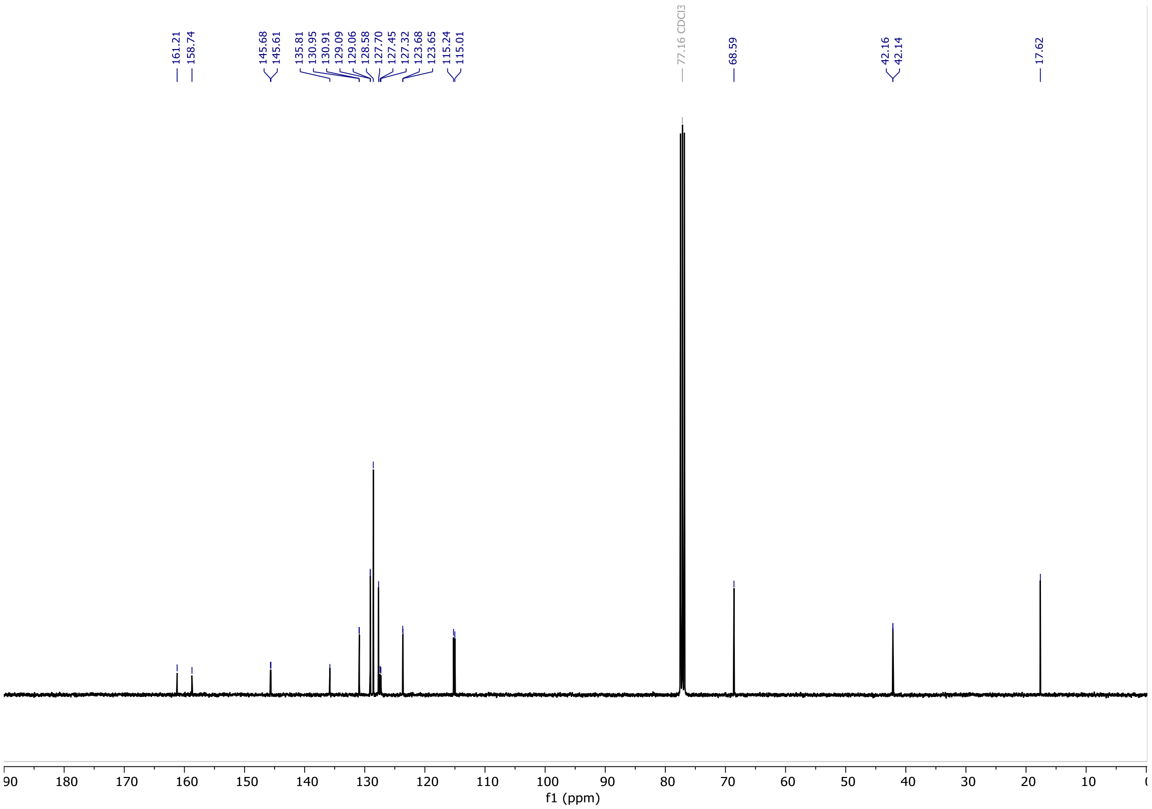

Figure S44 – **$^{13}C\{^{1}H\}$ NMR (101 MHz, $CDCl_3$, 296 K)** spectrum of compound **17**.

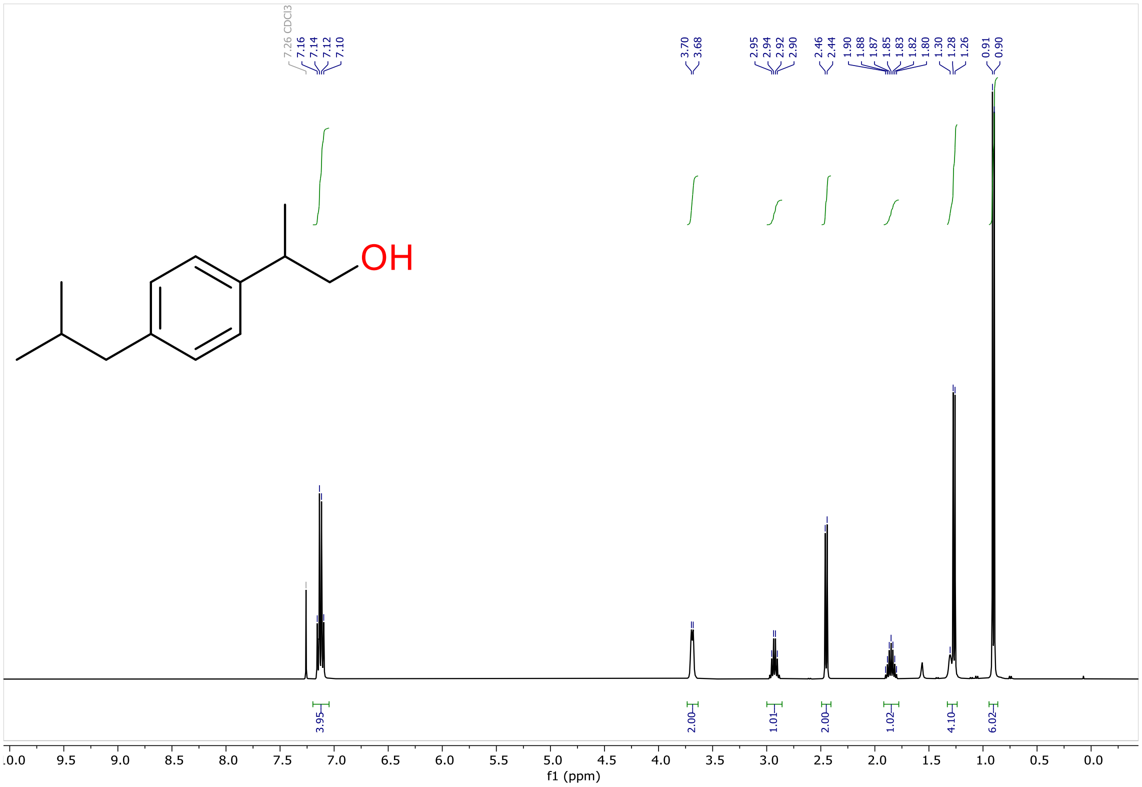

Figure S45 – **^{1}H NMR (400 MHz, $CDCl_3$, 296 K)** spectrum of compound **18**.

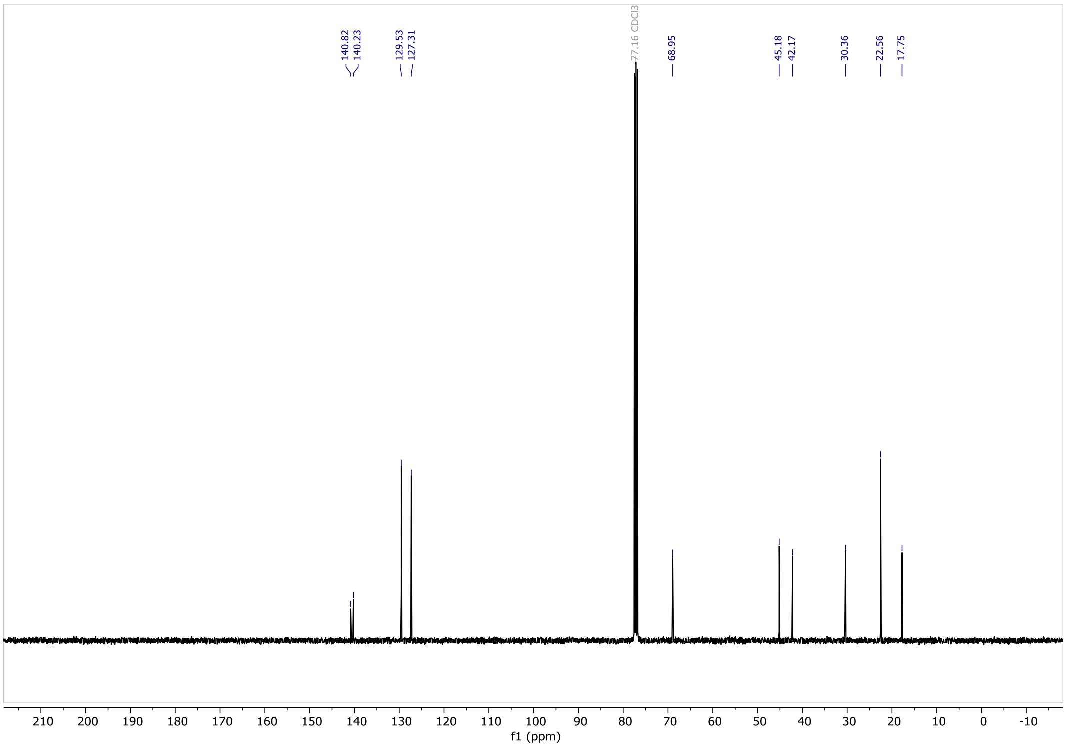

Figure S46 – **^{13}C{^{1}H} NMR (101 MHz, $CDCl_3$, 296 K)** spectrum of compound **18**.

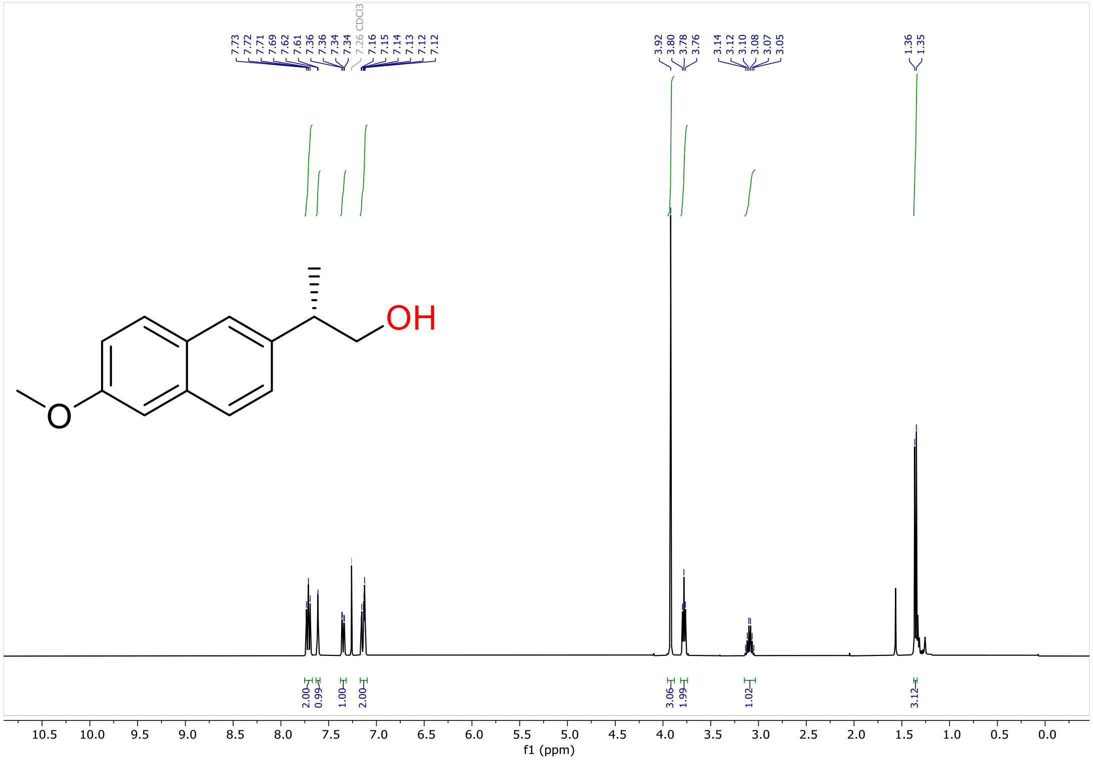

Figure S47 – **^{1}H NMR (400 MHz, $CDCl_3$, 296 K)** spectrum of compound **19**.

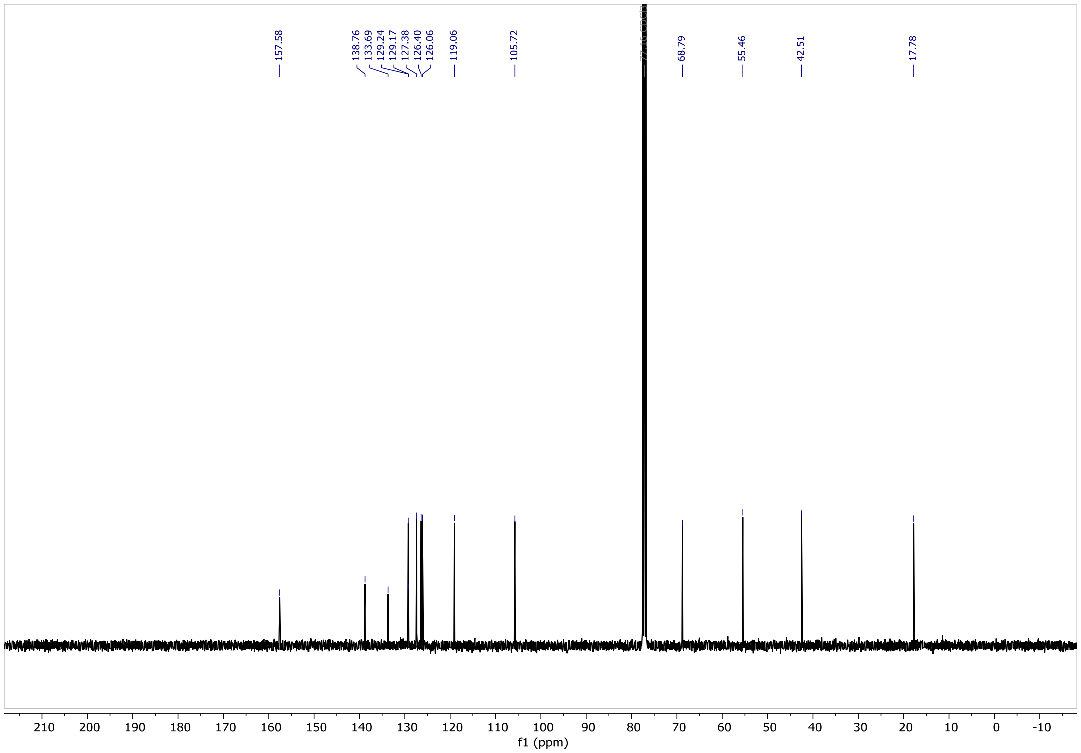

Figure S48 – **$^{13}C\{^{1}H\}$ NMR (101 MHz, $CDCl_3$, 296 K)** spectrum of compound **19**.

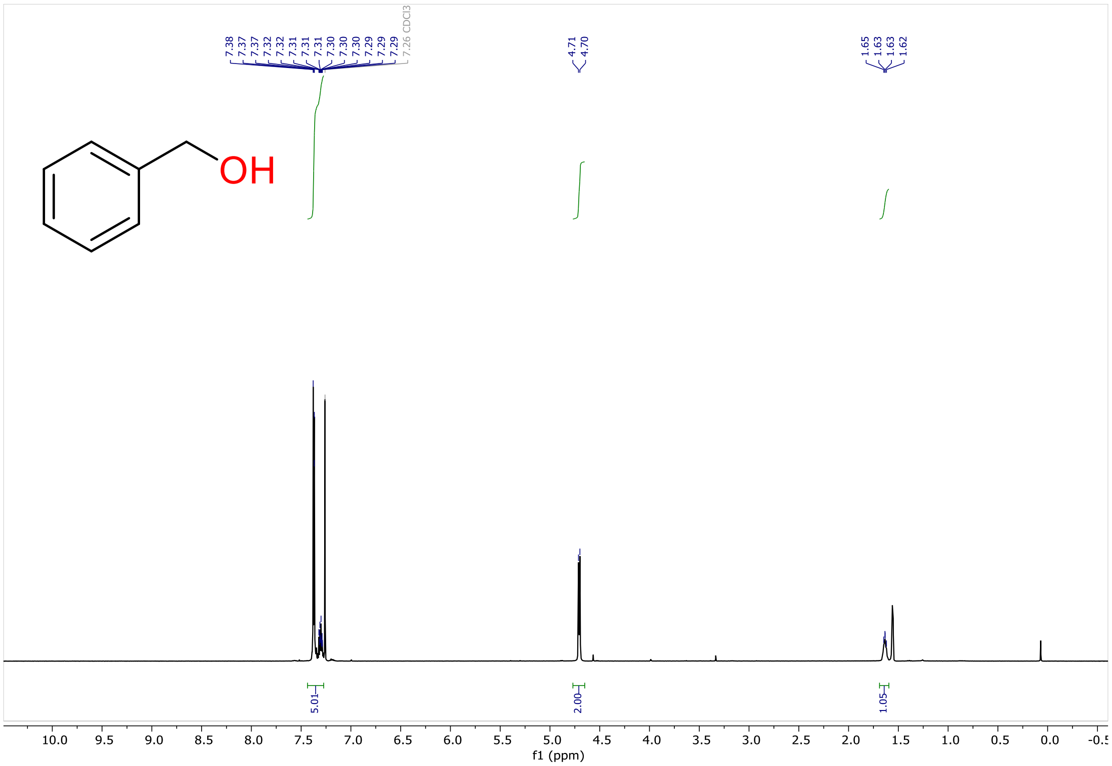

Figure S49 – **^{1}H NMR (400 MHz, $CDCl_3$, 296 K)** spectrum of compound **20**.

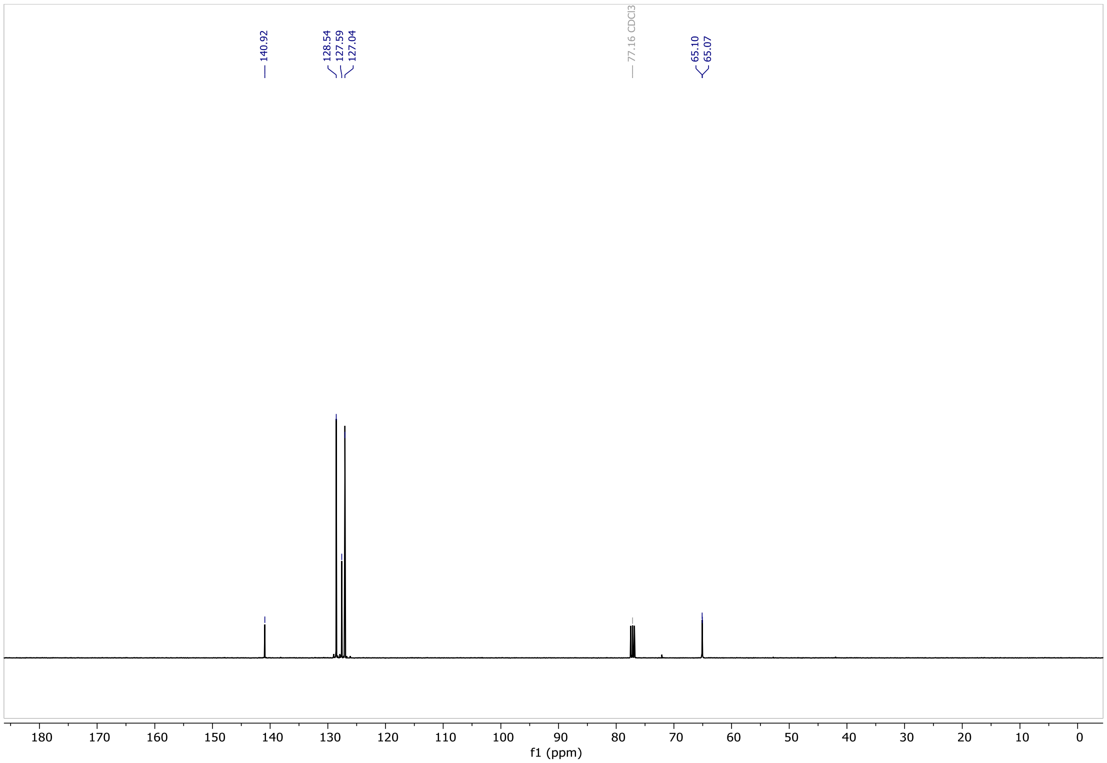

Figure S50 – **$^{13}C\{^{1}H\}$ NMR (101 MHz, $CDCl_3$, 296 K)** spectrum of compound **20**.

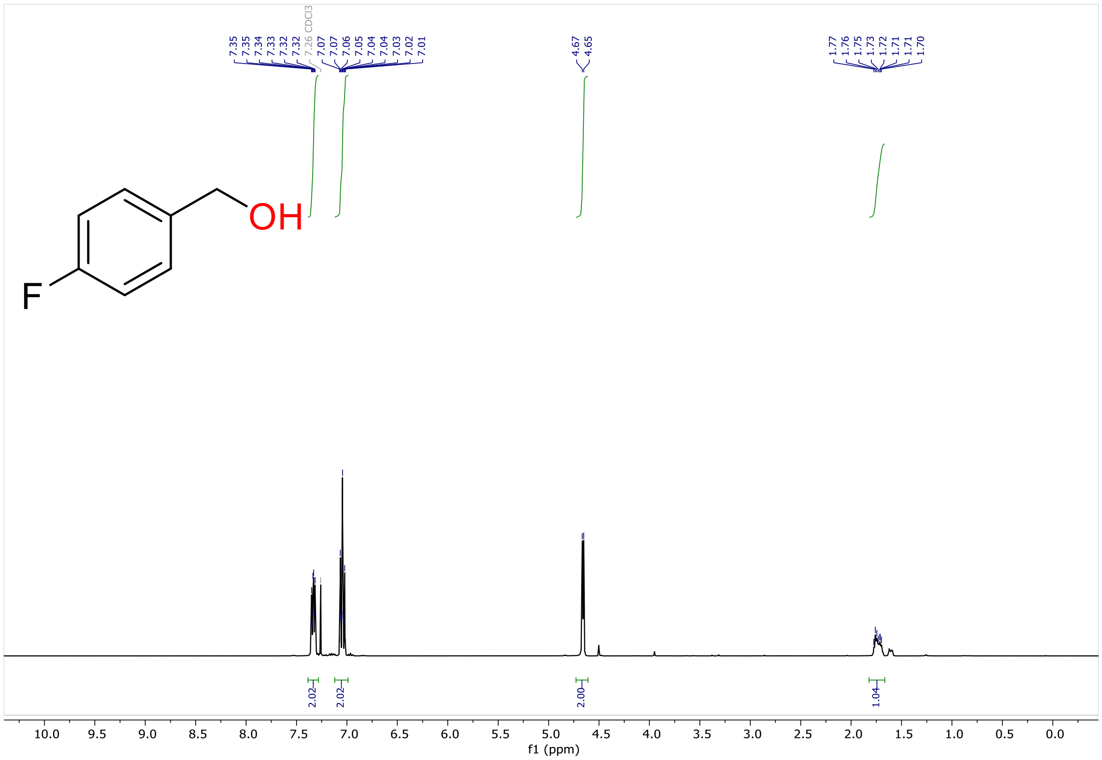

Figure S51 – **^{1}H NMR (400 MHz, $CDCl_3$, 296 K)** spectrum of compound **21**.

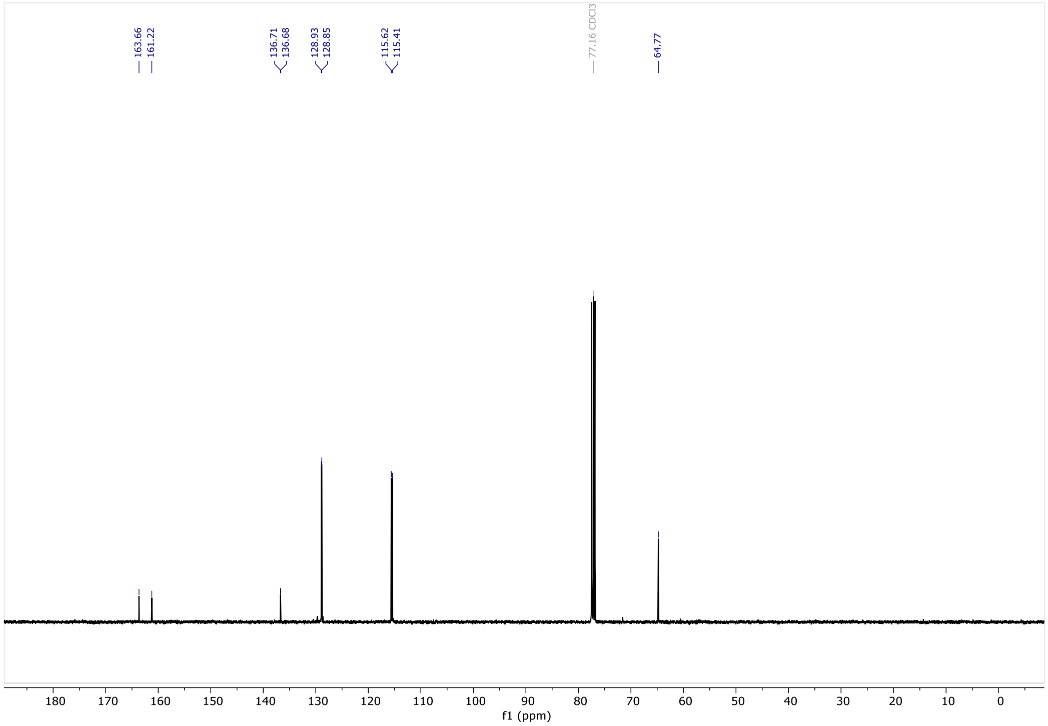

Figure S52 – **^{13}C{^{1}H} NMR (101 MHz, $CDCl_3$, 296 K)** spectrum of compound **21**.

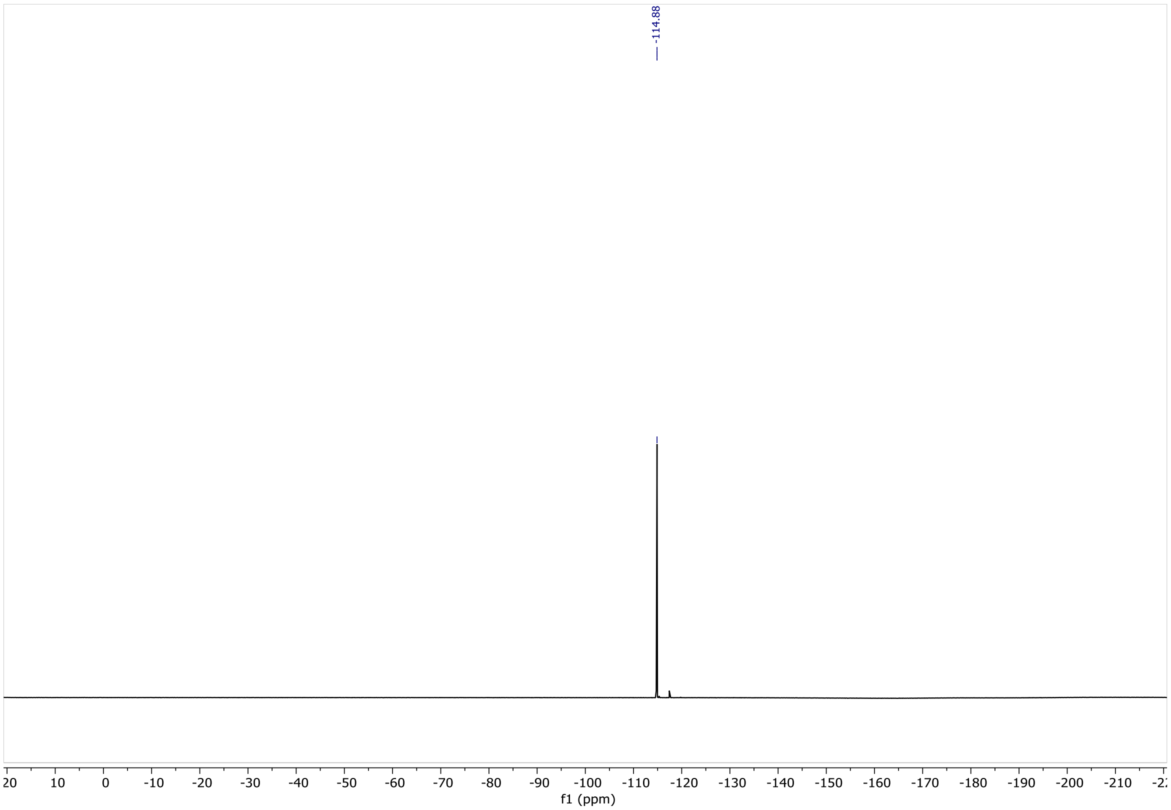

Figure S53 – $^{19}F\{^1H\}$ NMR (376 MHz, $CDCl_3$, 296 K) spectrum of compound **21**.

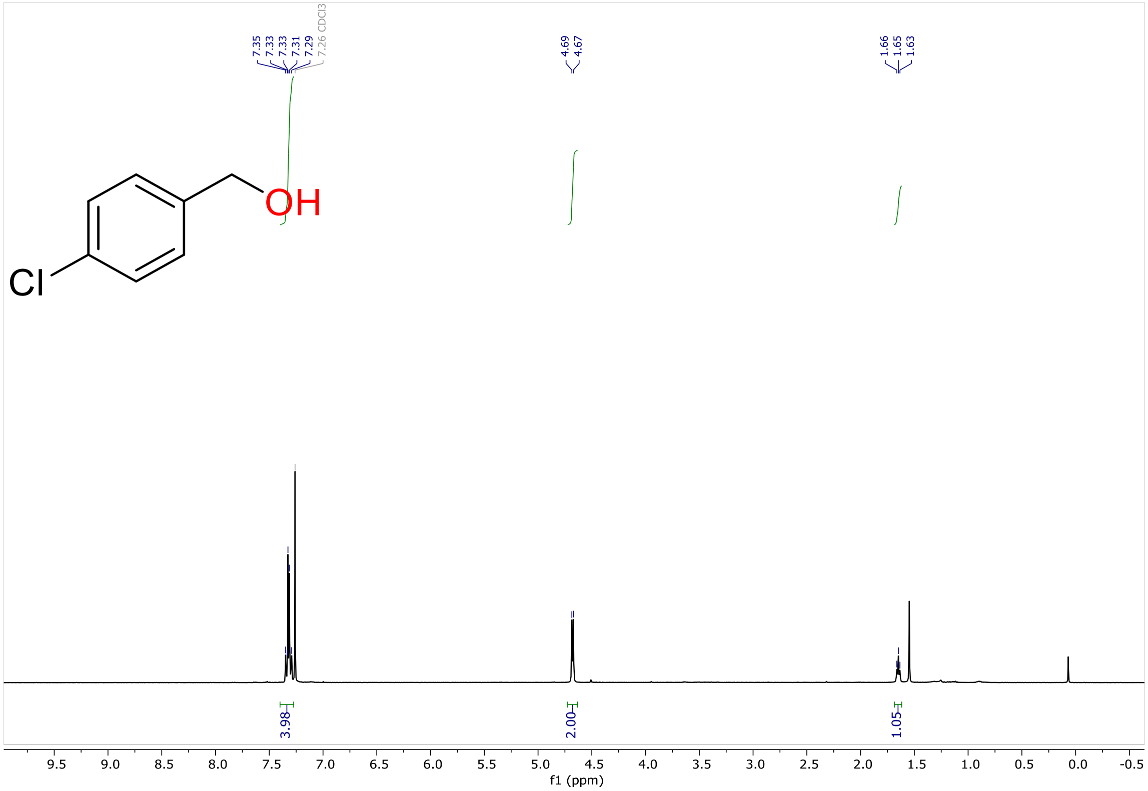

Figure S54 – ¹H NMR (400 MHz, CDCl₃, 296 K) spectrum of compound **22**.

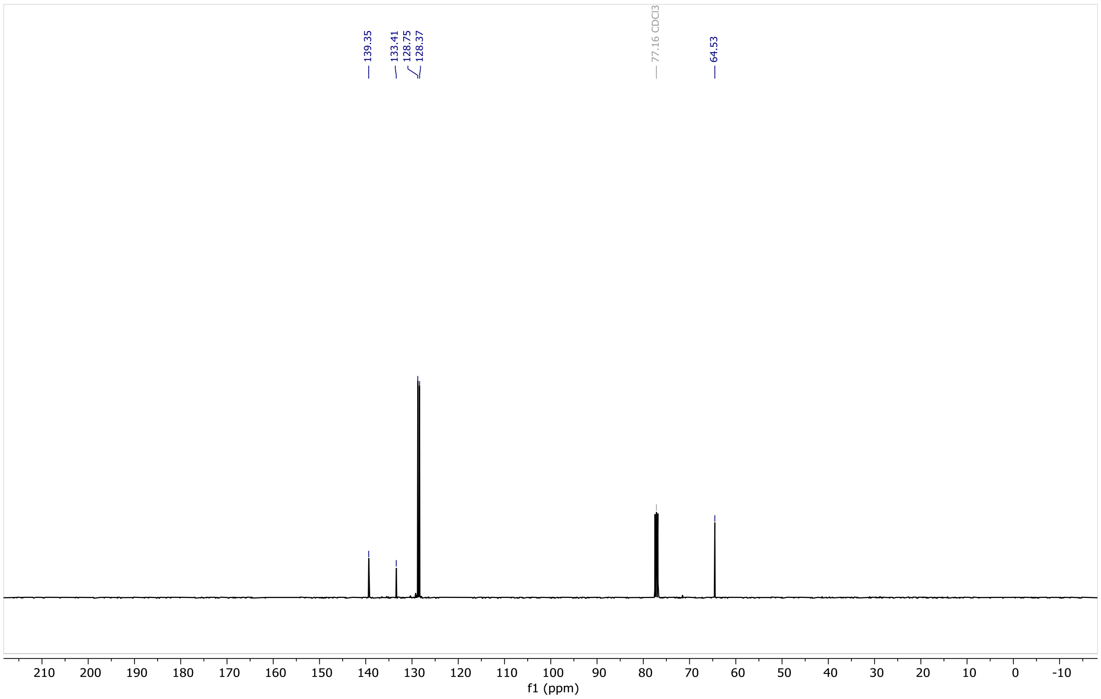

Figure S55 – ¹³C{¹H} NMR (101 MHz, CDCl₃, 297 K) spectrum of compound **22**.

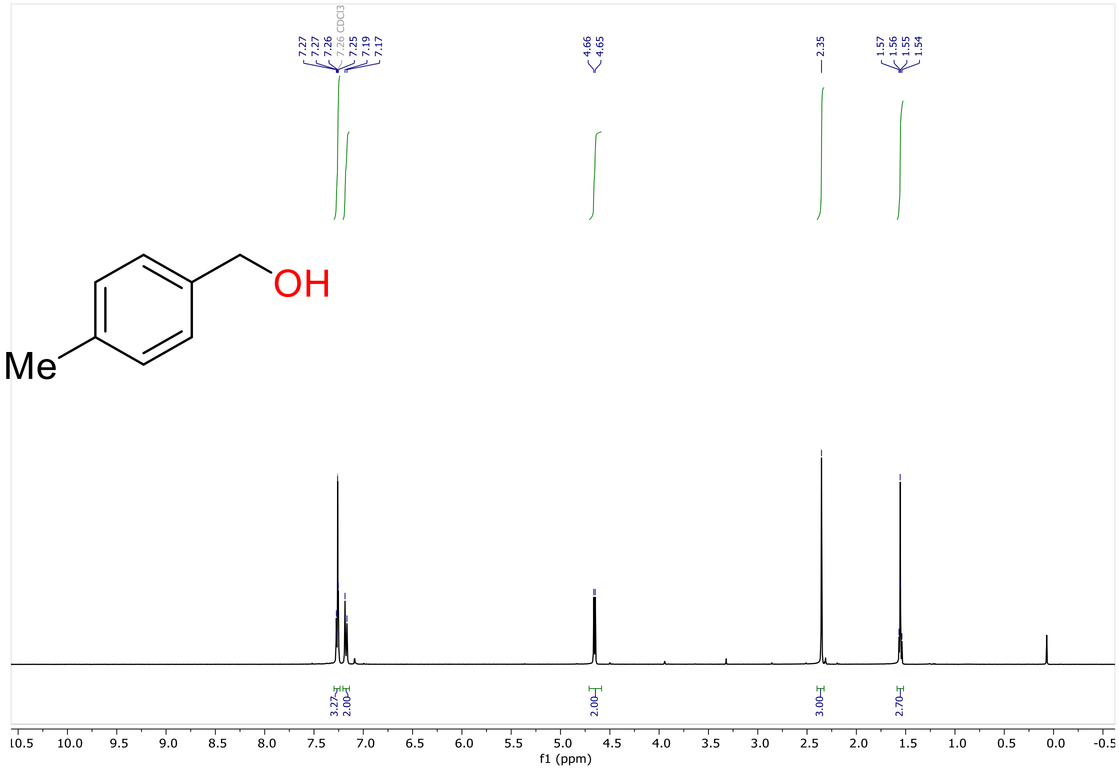

Figure S56 – **^{1}H NMR (400 MHz, $CDCl_3$, 296 K)** spectrum of compound **23**.

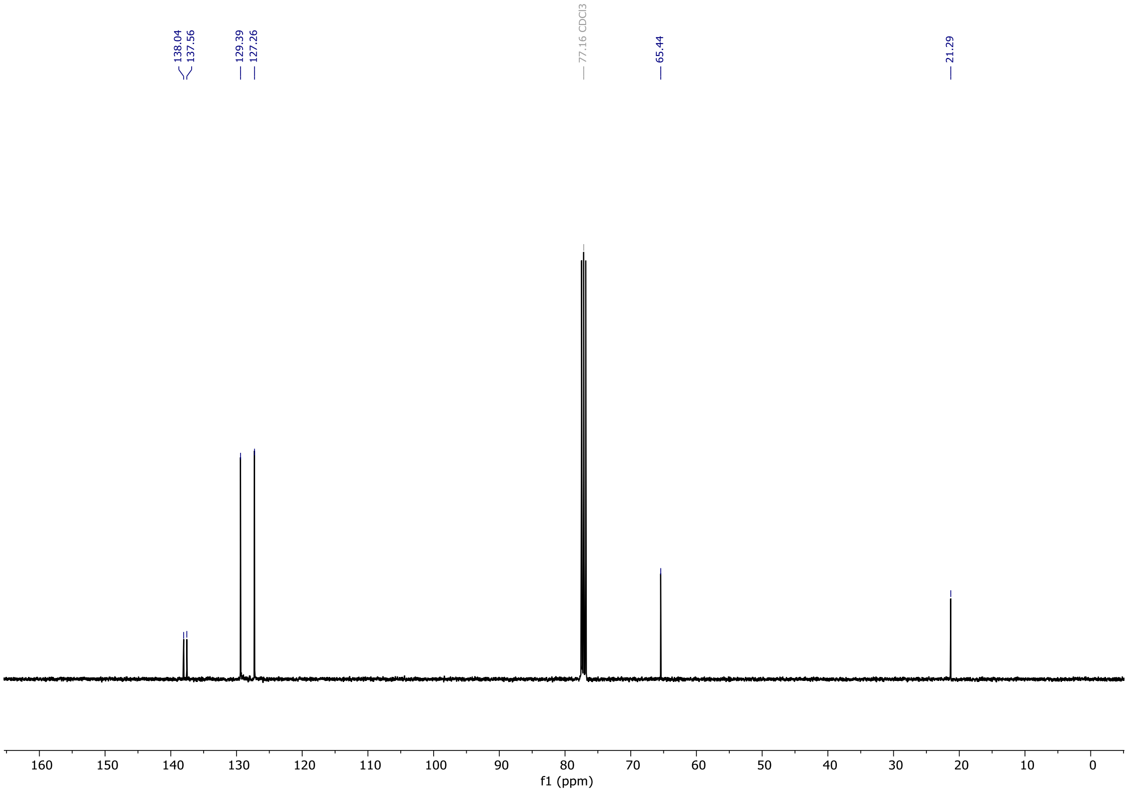

Figure S57 – $^{13}C\{^{1}H\}$ NMR (101 MHz, $CDCl_3$, 296 K) spectrum of compound **23**.

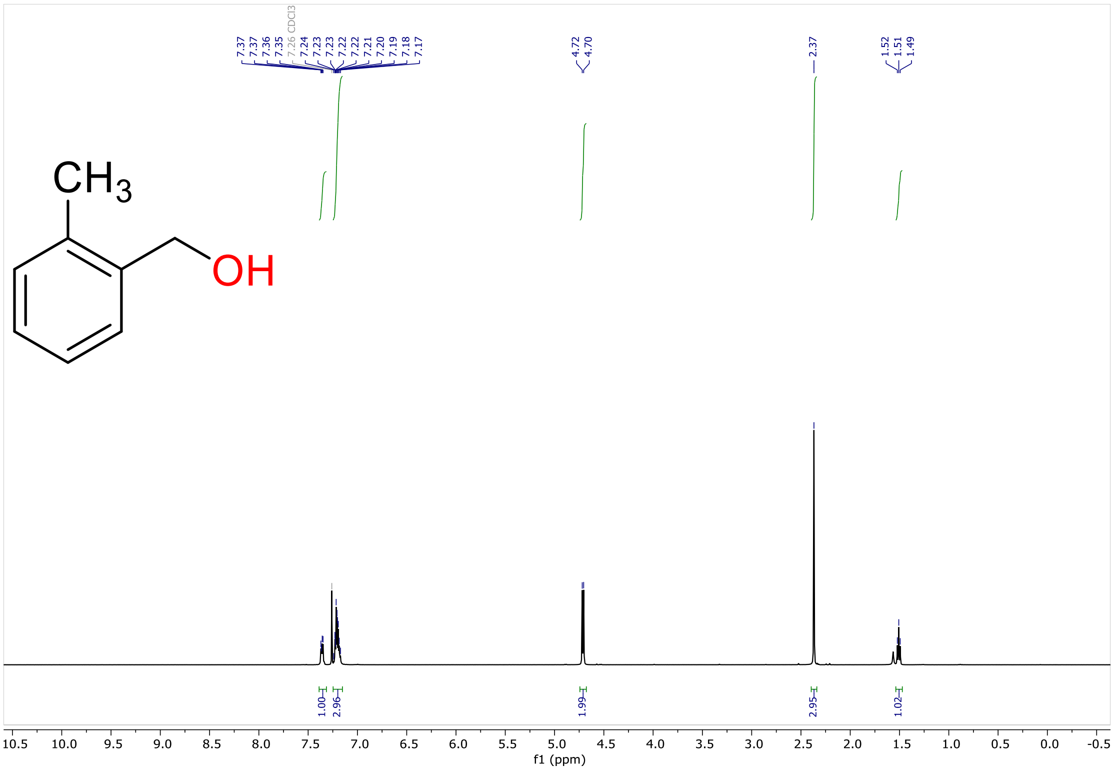

Figure S58 – ¹H NMR (400 MHz, CDCl₃, 296 K) spectrum of compound **24.**

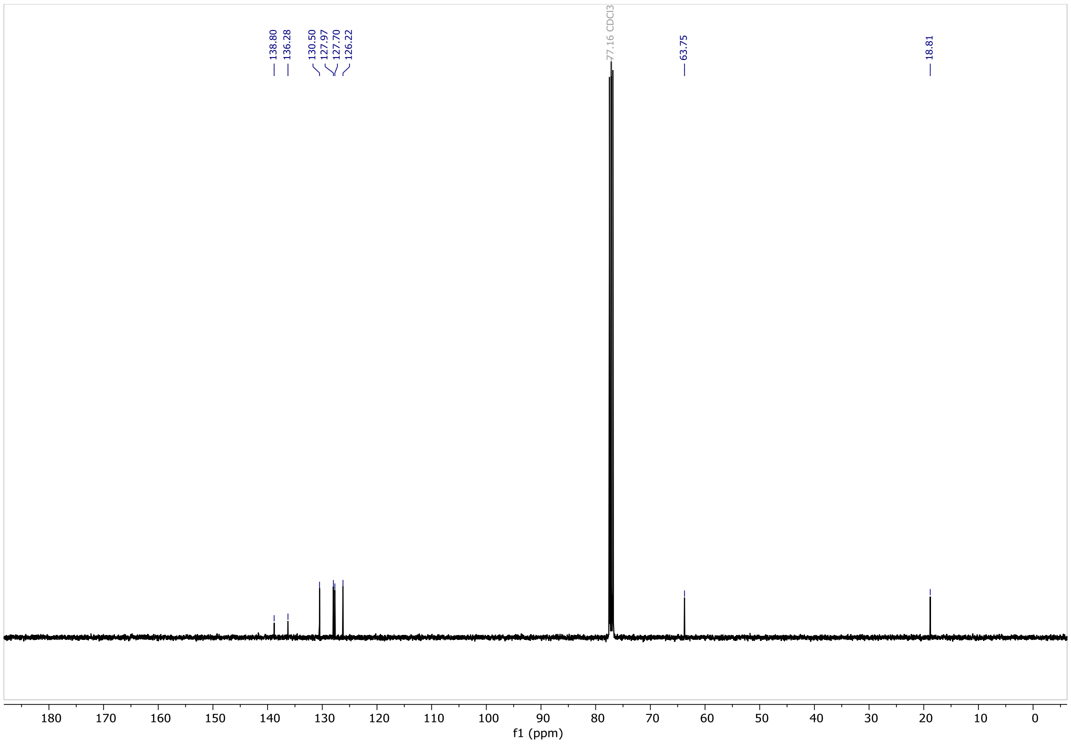

Figure S59 – ¹³C{¹H} NMR (101 MHz, CDCl₃, 296 K) spectrum of compound **24**.

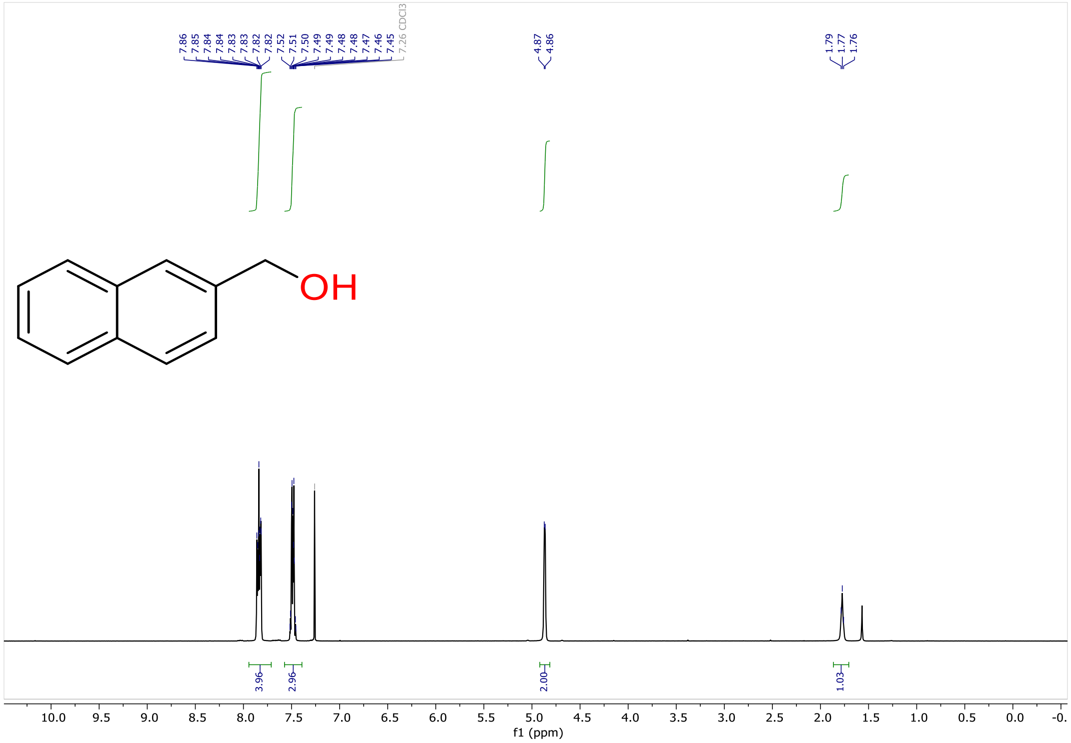

Figure S60 – **^{1}H NMR (400 MHz, $CDCl_3$, 296 K)** spectrum of compound **25**.

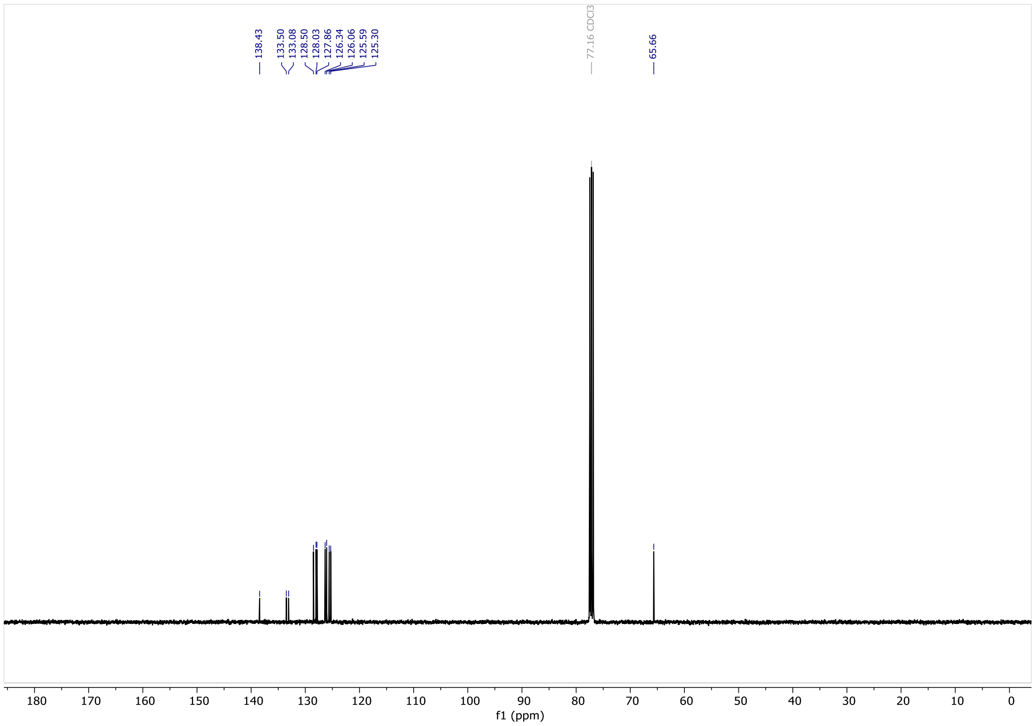

Figure S61 – **^{13}C{^{1}H} NMR (101 MHz, $CDCl_3$, 296 K)** spectrum of compound **25**.

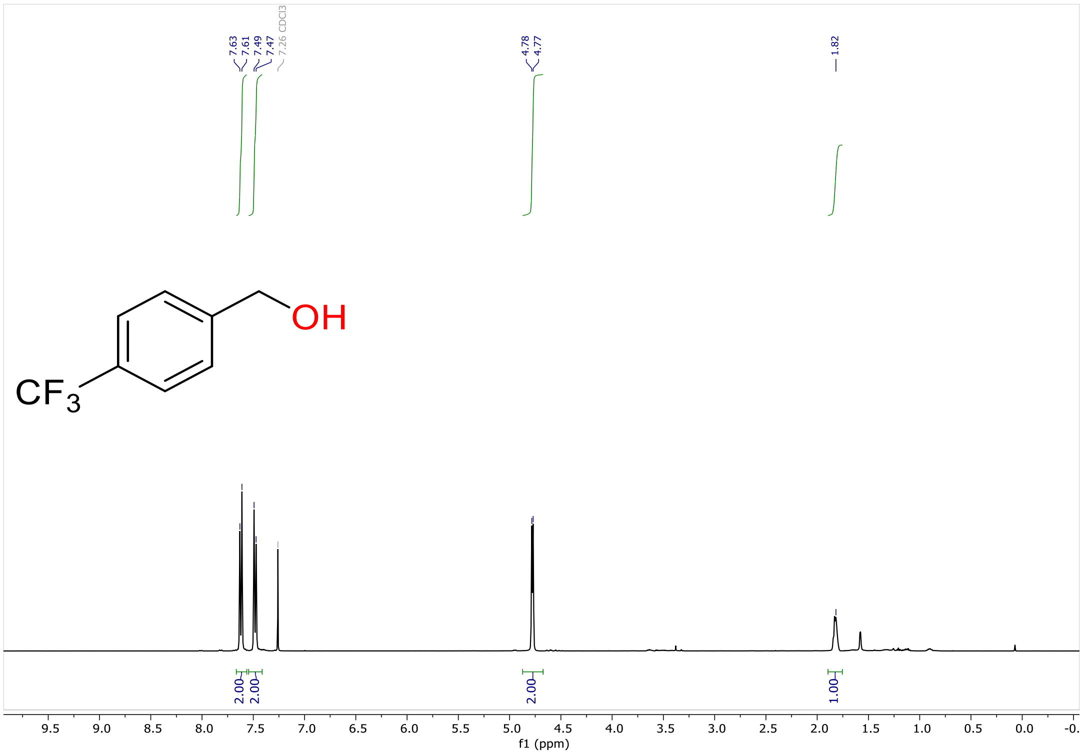

Figure S62 – **^{1}H NMR (400 MHz, $CDCl_3$, 296 K)** spectrum of compound **27**.

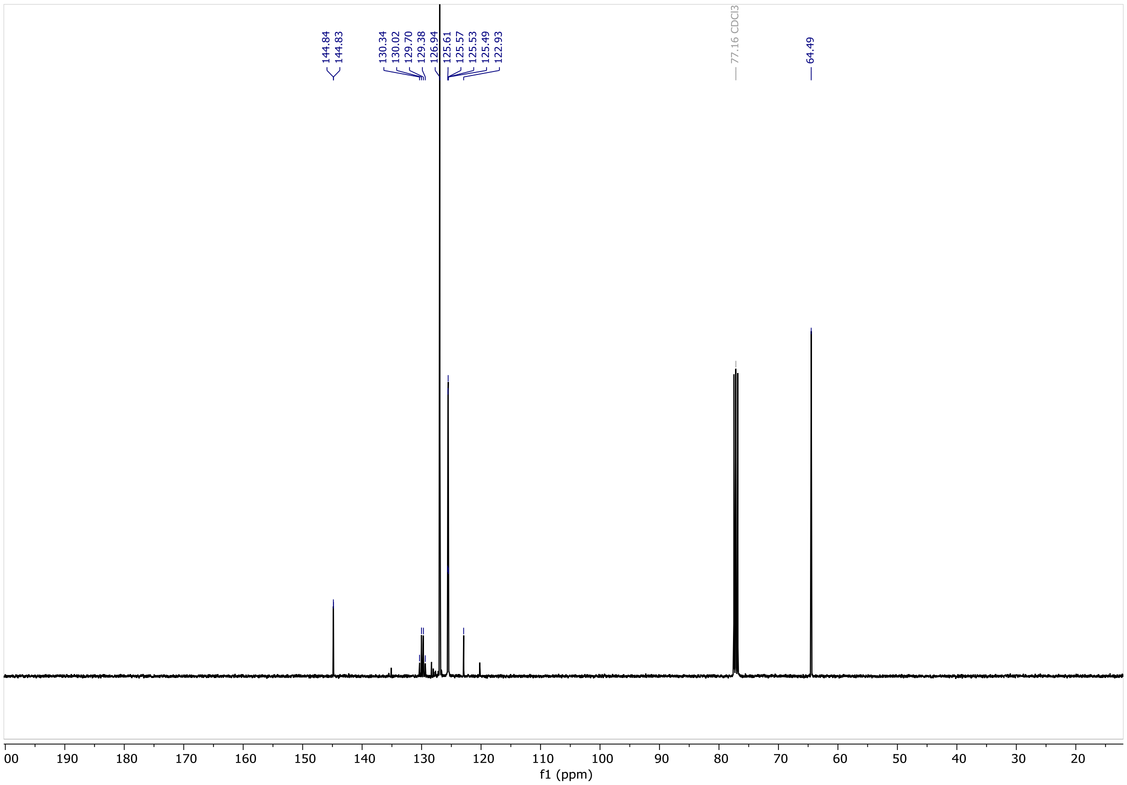

Figure S63 – **$^{13}C\{^{1}H\}$ NMR (101 MHz, $CDCl_3$, 297 K)** spectrum of compound **27**.

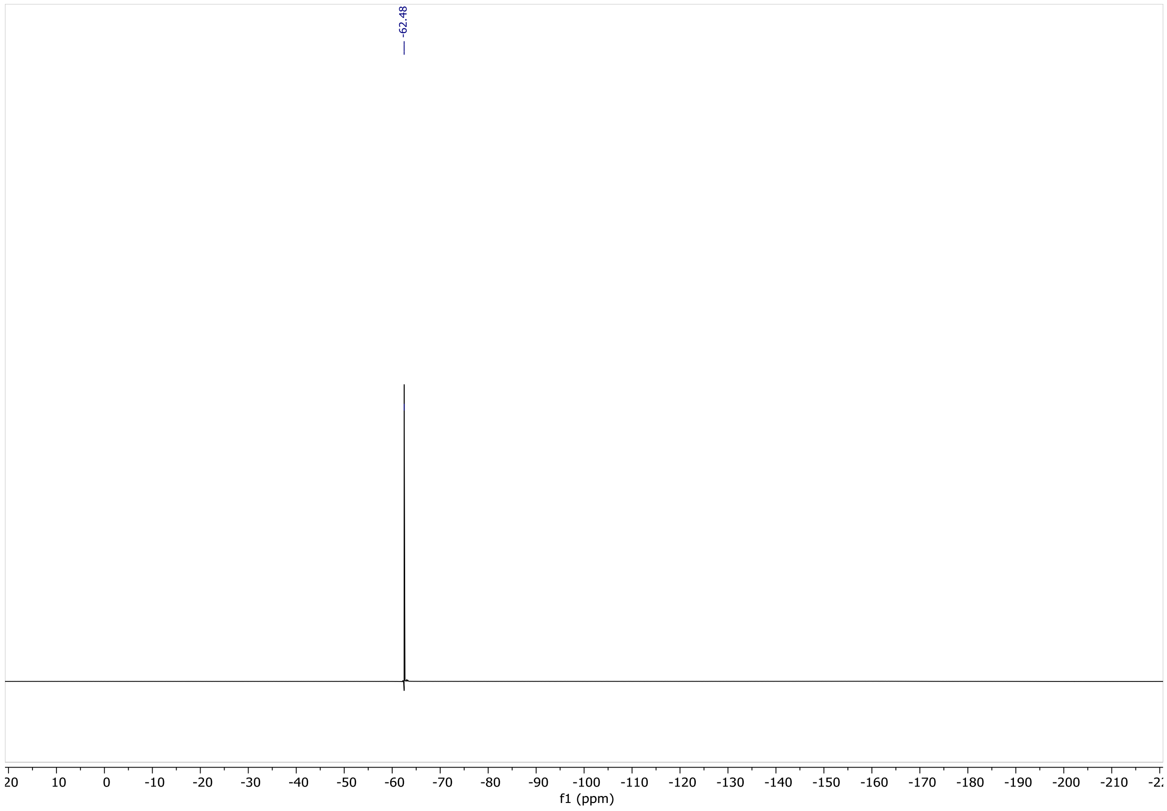

Figure S64 – **$^{19}F\{^{1}H\}$ NMR (376 MHz, $CDCl_3$, 296 K)** spectrum of compound **27**.

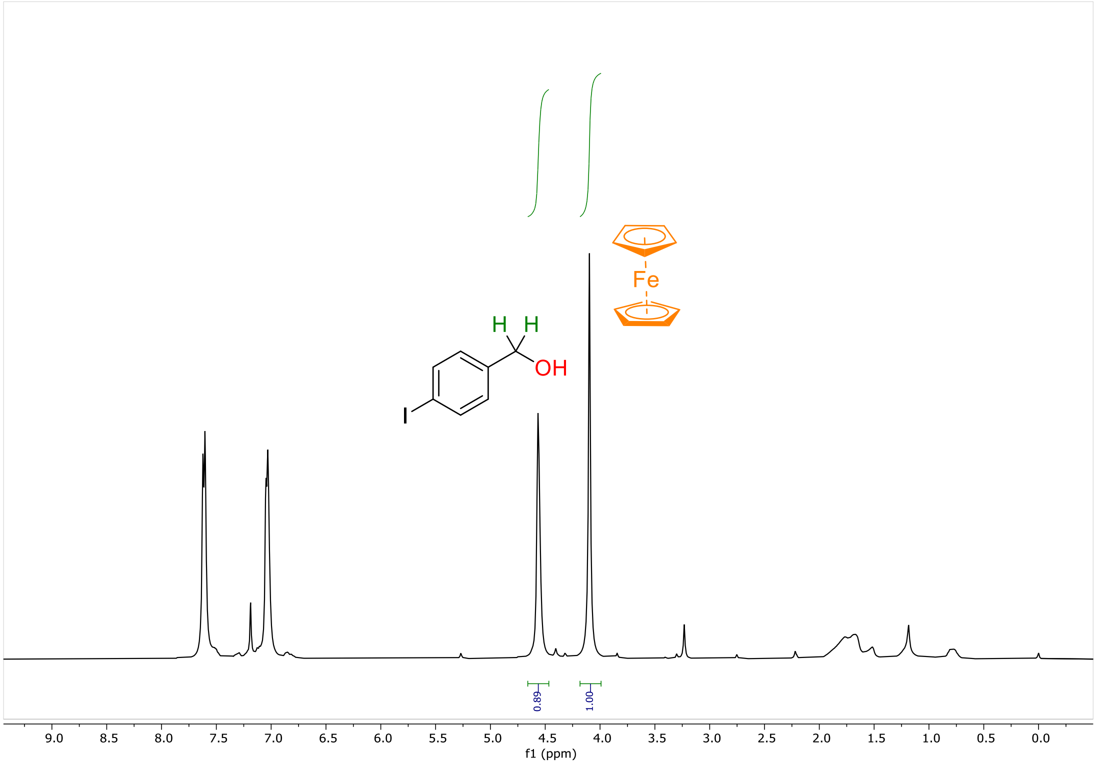

Figure S65 – **^{1}H NMR (400 MHz, $CDCl_3$, 296 K)** spectrum of compound **28.**

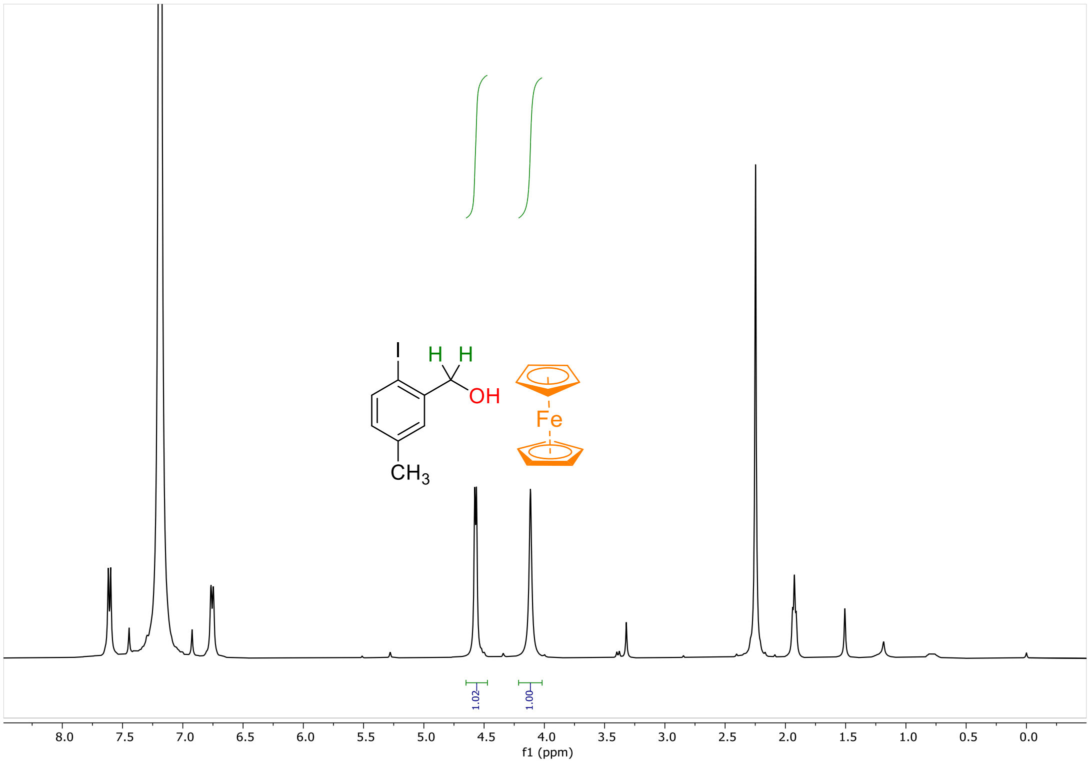

Figure S66 – **^{1}H NMR (400 MHz, $CDCl_3$, 296 K)** spectrum of compound **29**.

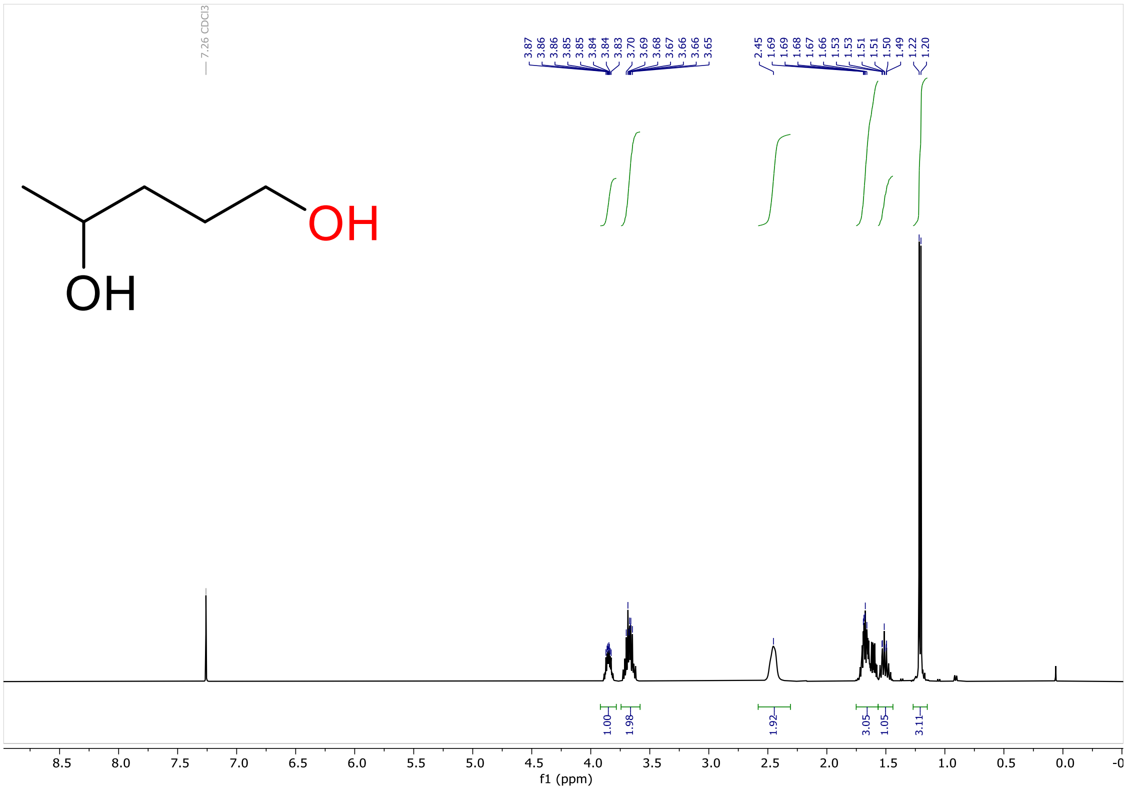

Figure S67 – **^{1}H NMR (400 MHz, $CDCl_3$, 296 K)** spectrum of compound **30.**

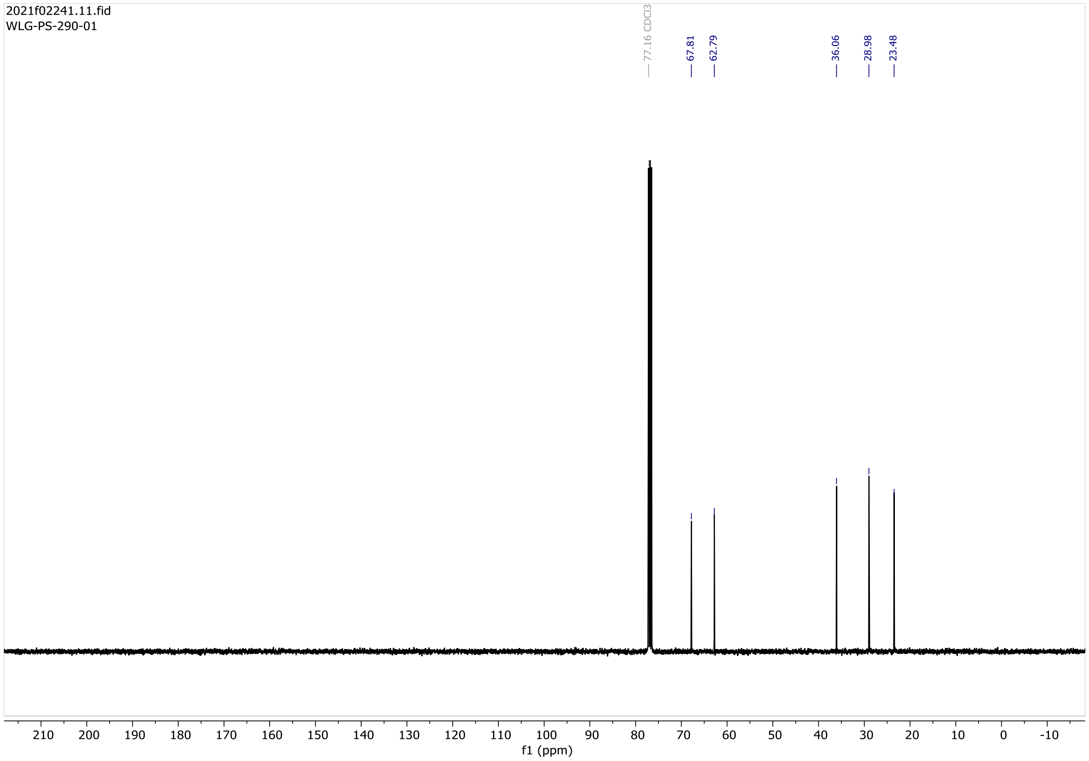

Figure S68 – **$^{13}C\{^{1}H\}$ NMR (101 MHz, $CDCl_3$, 296 K)** spectrum of compound **30**.

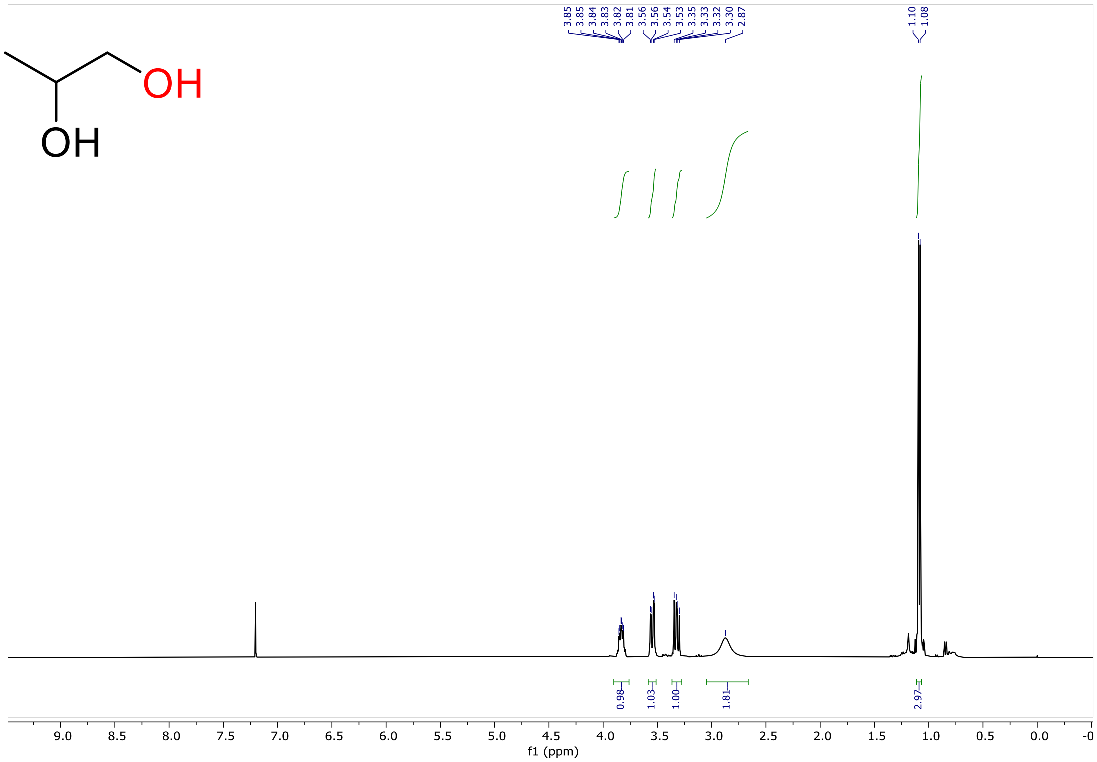

Figure S69 – **^{1}H NMR (400 MHz, $CDCl_3$, 296 K)** spectrum of compound **31.**

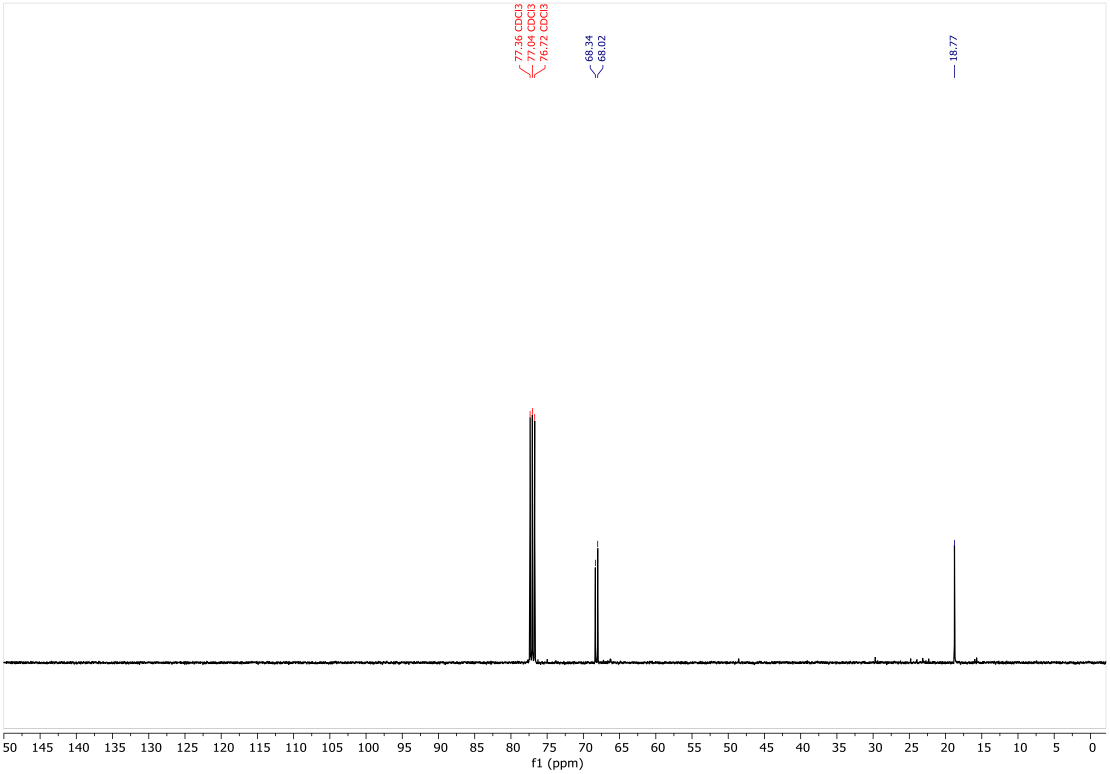

Figure S70 – **$^{13}C\{^{1}H\}$ NMR (101 MHz, $CDCl_3$, 296 K)** spectrum of compound **31**.

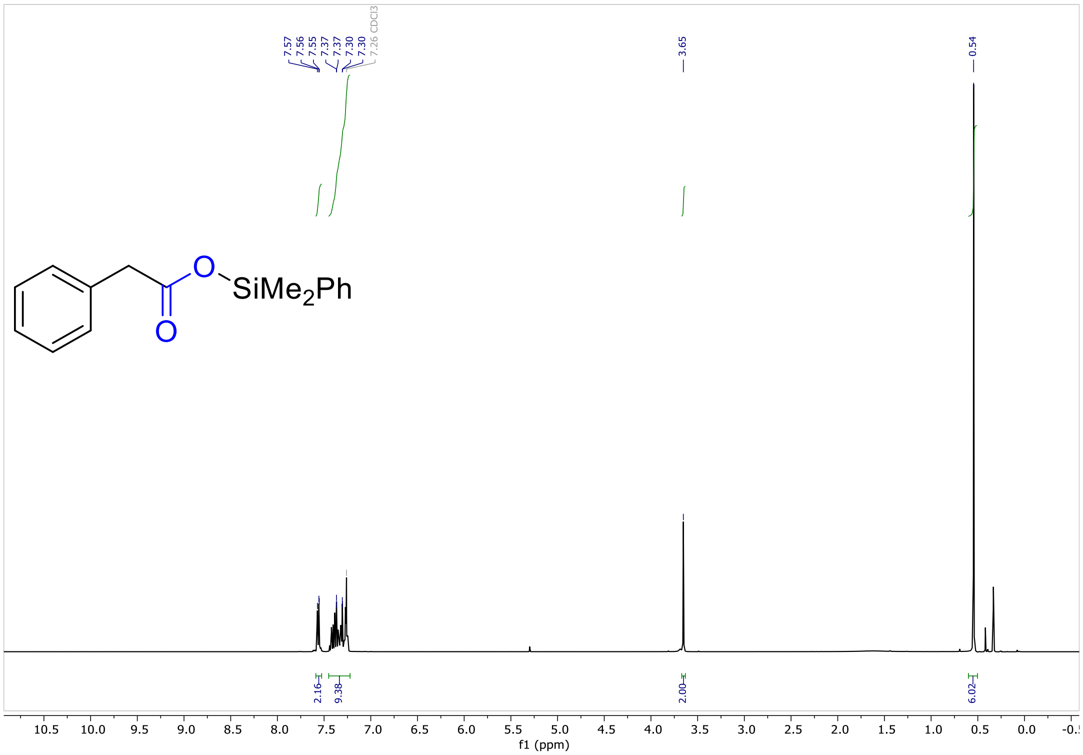

Figure S71 – **^{1}H NMR (400 MHz, CDCl$_3$, 296 K)** spectrum of compound **32**.

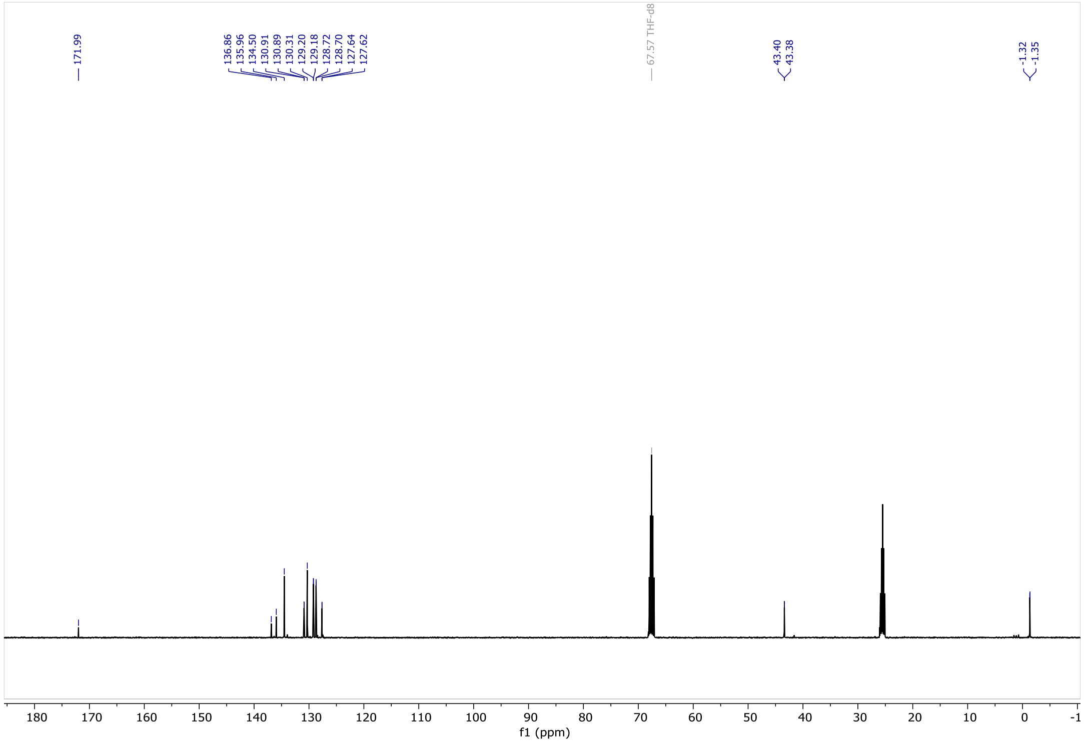

Figure S72 – **^{13}C{^{1}H} NMR (101 MHz, THF-*d*$_8$, 296 K)** spectrum of compound **32**.

14. References

(1) Martínez-Ferraté, O.; Chatterjee, B.; Werlé, C.; Leitner, W., Hydrosilylation of carbonyl and carboxyl groups catalysed by Mn(I) complexes bearing triazole ligands. *Catal. Sci. Technol.* **2019,** *9* (22), 6370-6378.

(2) Bruneau-Voisine, A.; Wang, D.; Roisnel, T.; Darcel, C.; Sortais, J.-B., Hydrogenation of ketones with a manganese PN^3P pincer pre-catalyst. *Catal. Commun.* **2017,** *92*, 1-4.

(3) Gawali, S. S.; Pandia, B. K.; Gunanathan, C., Manganese(I)-Catalyzed alpha-Alkenylation of Ketones Using Primary Alcohols. *Org. Lett.* **2019,** *21* (10), 3842-3847.

(4) Kumar, A.; Goyal, V.; Sarki, N.; Singh, B.; Ray, A.; Bhaskar, T.; Bordoloi, A.; Narani, A.; Natte, K., Biocarbon Supported Nanoscale Ruthenium Oxide-Based Catalyst for Clean Hydrogenation of Arenes and Heteroarenes. *ACS Sustain. Chem. Eng.* **2020,** *8* (41), 15740-15754.

(5) Brunel, J. M., Scope, limitations and mechanistic aspects in the selective homogeneous palladium-catalyzed reduction of alkenes under transfer hydrogen conditions. *Tetrahedron* **2007,** *63* (18), 3899-3906.

(6) Barman, M. K.; Das, K.; Maji, B., Selective Hydroboration of Carboxylic Acids with a Homogeneous Manganese Catalyst. *J. Org. Chem.* **2019,** *84* (3), 1570-1579.

(7) Bender, T. A.; Bergman, R. G.; Raymond, K. N.; Toste, F. D., A Supramolecular Strategy for Selective Catalytic Hydrogenation Independent of Remote Chain Length. *J. Am. Chem. Soc.* **2019,** *141* (30), 11806-11810.

(8) Chakraborty, S.; Dai, H.; Bhattacharya, P.; Fairweather, N. T.; Gibson, M. S.; Krause, J. A.; Guan, H., Iron-based catalysts for the hydrogenation of esters to alcohols. *J. Am. Chem. Soc.* **2014,** *136* (22), 7869-7872.

(9) Bodnar, B. S.; Vogt, P. F., An improved Bouveault-Blanc ester reduction with stabilized alkali metals. *J. Org. Chem.* **2009,** *74* (6), 2598-2600.

(10) Roberts, D. D.; Arant, M. E., Solvolysis Reactions - Relative Abilities of Cyclopentyl/Phenyl Groups to Stabilize an Electron-Deficient Carbon. *J. Org. Chem.* **1994,** *59* (21), 6464-6469.

(11) Sakai, N.; Kawana, K.; Ikeda, R.; Nakaike, Y.; Konakahara, T., $InBr_3$-Catalyzed Deoxygenation of Carboxylic Acids with a Hydrosilane: Reductive Conversion of Aliphatic or Aromatic Carboxylic Acids to Primary Alcohols or Diphenylmethanes. *Eur. J. Org. Chem.* **2011,** *2011* (17), 3178-3183.

(12) Zhou, F. Y.; She, J.; Wang, Y. G., Synthesis of a benzyl-protected analog of arenarioside, a trisaccharide phenylpropanoid glycoside. *Carbohydr. Res.* **2006,** *341* (15), 2469-2477.

(13) Shahane, S.; Louafi, F.; Moreau, J.; Hurvois, J.-P.; Renaud, J.-L.; van de Weghe, P.; Roisnel, T., Synthesis of Alkaloids ofGalipea officinalisby Alkylation of an α-Amino Nitrile. *Eur. J. Org. Chem.* **2008,** *2008* (27), 4622-4631.

(14) Schupbach, B.; Terfort, A., A divergent synthesis of oligoarylalkanethiols with Lewis-basic N-donor termini. *Org. Biomol. Chem.* **2010,** *8* (15), 3552-3562.

(15) Friest, J. A.; Maezato, Y.; Broussy, S.; Blum, P.; Berkowitz, D. B., Use of a robust dehydrogenase from an archael hyperthermophile in asymmetric catalysis-dynamic reductive kinetic resolution entry into (S)-profens. *J. Am. Chem. Soc.* **2010,** *132* (17), 5930-5931.

(16) Koul, S.; Koul, J. L.; Singh, B.; Kapoor, M.; Parshad, R.; Manhas, K. S.; Taneja, S. C.; Qazi, G. N., Trichosporon beigelli esterase (TBE): a versatile esterase for the resolution of economically important racemates. *Tetrahedron-Asymmetry* **2005,** *16* (15), 2575-2591.

(17) Bhattacharya, P.; Krause, J. A.; Guan, H. R., Iron Hydride Complexes Bearing Phosphinite-Based Pincer Ligands: Synthesis, Reactivity, and Catalytic Application in Hydrosilylation Reactions. *Organometallics* **2011,** *30* (17), 4720-4729.

(18) Shao, Z. H.; Zhong, R.; Ferraccioli, R.; Li, Y. B.; Liu, Q., General and Phosphine-Free Cobalt-Catalyzed Hydrogenation of Esters to Alcohols. *Chin. J. Chem.* **2019,** *37* (11), 1125-1130.

(19) Ojima, Y.; Yamaguchi, K.; Mizuno, N., An Efficient Solvent-Free Route to Silyl Esters and Silyl Ethers. *Adv. Synth. Catal.* **2009,** *351* (9), 1405-1411.

(20) Stoll, E. L.; Tongue, T.; Andrews, K. G.; Valette, D.; Hirst, D. J.; Denton, R. M., A practical catalytic reductive amination of carboxylic acids. *Chemical Science* **2020,** *11* (35), 9494-9500.
